# From Mating to Milk Access: A Review of Reproductive Vocal Communication in Mice

**DOI:** 10.3389/fnbeh.2022.833168

**Published:** 2022-03-28

**Authors:** Sara Capas-Peneda, Yolanda Saavedra Torres, Jan-Bas Prins, I. Anna S. Olsson

**Affiliations:** ^1^Biological Research Facility, Francis Crick Institute, London, United Kingdom; ^2^i3S – Instituto de Investigação e Inovação em Saúde, Universidade do Porto, Porto, Portugal; ^3^ICBAS – School of Medicine and Biomedical Sciences, University of Porto, Porto, Portugal; ^4^Leiden University Medical Centre, Leiden, Netherlands

**Keywords:** mouse, vocal communication, ultrasonic vocalisation (USV), courtship, neonatal vocalisation

## Abstract

Vocalisations play a central role in rodent communication, especially in reproduction related behaviours. In adult mice (*Mus musculus*) the emission of ultrasonic vocalisations (USVs) has been observed in courtship and mating behaviour, especially by males. These have been found to have distinctive individual signatures that influence female choice of mating partner. The most recent findings show that vocal communication also has a role in parental cooperation, in that female mice communicate with male partners in ultrasonic frequencies to induce paternal behaviour. Infant vocalisations form the other important part of reproductive vocal communication. Although born deaf, neonatal mice are capable of producing vocalisations since birth. As an altricial species, successful mother-infant communication is essential for survival, and these vocalisations are important modulators of maternal behaviour. Three main types of infant vocalisations have been identified and characterised. Most research has addressed pure USVs, related to stressful situations (e.g., cold, isolation, handling, presence of unfamiliar males or predators), which usually elicit maternal search and retrieval. In addition, broad-band spectrum signals, emitted post-partum during cleaning of foetal membranes, inhibit biting and injury by adults and “wriggling calls,” emitted during suckling, release maternal behaviour (such as licking). Several variables have been identified to modulate vocalisations in mice, including individual characteristics such as strain/genotype, age, sex, and experimental factors such as pharmacological compounds and social context. In recent years, there has been a big increase in the knowledge about the characteristics of vocal communication in rodents due to recent technological advances as well as a growing interest from the neuroscience community. Vocalisation analysis has become an essential tool for phenotyping and evaluating emotional states. In this review, we will (i) provide a comprehensive summary of the current knowledge on mouse reproductive vocal communication and (ii) discuss the most recent findings in order to provide a broad overview on this topic.

## Introduction

Mice live in a complex social environment. Communication between conspecifics occurs by different types of cues that provide distinct information. Recent evidence suggest that body language, such as body posture and facial expressions play a part in social communication (Ebbesen and Froemke, 2021). But, the most well-studied forms of communication are still the odour cues, mainly pheromones, and vocal communication that play a role in informing on the location of animals, presence of food or threat, sexual attraction, courtship and dam-pup interactions (Bind et al., 2013; Portfors and Perkel, 2014).

Vocal communication has been gathering much interest since the first description of ultrasonic vocalisations (USVs) in neonatal mice by Zippelius and Schleidt (1956). USVs (in the range of 30--90 kHz)^1^ are now known to serve important functions in sexual behaviour and pup and dam communication in mice.

Furthermore, although significantly less studied, mice are also able to produce vocalisations in the audible (to the human ear) range that serve different functions which are generally related to negative affective states, such as in response to a predatory attack (Blanchard et al., 1998), human handling (Whitney, 1969), or by females in response to male sexual behaviour when in a non-receptive state (Sugimoto et al., 2011; Neunuebel et al., 2015).

Communication in the ultrasonic range is thought to pose an evolutionary advantage due to the potential predatory evasion since the most common predators of house mice have hearing ranges below the frequencies of USVs (Musolf and Penn, 2012). It seems that USVs appeared first in neonatal mice, and it is theorised that they were later exploited by males. This is due to the fact that neonatal USVs reduce female aggression and most matings occur in the post-partum oestrous, when the female is especially receptive to neonatal USVs (Whitney et al., 1973).

Ultrasonic vocalisations can be grouped according to internal frequency changes, duration and sonographic shape in ten different categories, as proposed by Scattoni et al. (2008) (see Table 1).

**TABLE 1:** Different types of ultrasonic vocalisations (Scattoni et al., 2008).

Denomination	Characteristics
Complex calls	One syllable containing two or more directional changes in pitch, each ≥6.25 kHz
Harmonics	One main call, resembling the complex calls, but with additional calls of different frequencies surrounding the main call
Two-syllable calls	Consist of two components: a main call (flat or downward) with an additional punctuated component toward the end
Upward-modulated calls	Exhibit a continuous increase in pitch that was ≥12.5 kHz, with a terminal dominant frequency at least 6.25 kHz more than the pitch at the beginning of the vocalisation.
Downward-modulated calls	Exhibit a continuous decrease in pitch that was ≥12.5 kHz, with a terminal dominant frequency at least 6.25 kHz less than the pitch at the beginning of the vocalisation
Flat calls	Display a constant beginning and the ending of the pitch frequency remained constant -≤3 kHz of each other.
Chevron calls	Resemble an “inverted-U,” which was identified by a continuous increase in pitch ≥ 12.5 kHz followed by a decrease that was ≤ 6.25 kHz
Short calls	punctuated and shorter than 5 ms
Composite calls	Formed by two harmonically independent components, emitted simultaneous
Frequency steps	Instantaneous frequency changes appearing as a vertically discontinuous “step” on a spectrogram, but with no interruption in time.

Murine vocalisations are produced by airflow through the larynx. The mouse larynx is a tube-shaped musculocartilaginous organ through which the air passes from the pharynx to the trachea and, along with other functions, is the organ of phonation. As in other mammals, it is composed by three unpaired cartilages (epiglottic, thyroid, and cricoid cartilages) and two arythenoid cartilages. The vocal folds are located in the vestibule of the larynx, where there is also a large laryngeal recess. It is when air passes during expiration generating vibration of the vocal folds that sound is emitted (Navarro et al., 2017a).

Different mechanisms are involved when USV versus audible sounds are produced (Roberts, 1975). Audible sounds are produced by the vibration of vocal cords in the larynx, in a similar process to other mammals. In contrast, ultrasonic vocalisations are produced by a whistle like mechanism that is currently not completely understood. Currently, two main theories have been proposed, the planar impinging model by Mahrt et al. (2016) that suggests USVs are produced by an intralaryngeal air jet created by a glottal constriction impinging on the interior surface of the thyroid cartilage, and the edge-tone model proposed by Riede et al. (2017) that suggest USVs are produced by an interaction between the glottal exit and the edge of an intralaryngeal ventral pouch. Although they differ in exact mechanisms, both these theories indicate that glottal constriction and laryngeal muscle contraction are necessary to produce USVs.

Since USVs play an important role in mouse vocal communication, the hearing range of mice needs to be compatible with the frequencies at which these animals vocalise. Hearing range is dependent on the physics of the structures involved in the middle and inner ear and, thus, on the anatomy of the ear. The soundwaves are received by the auricle, in the external ear, and are carried in the external acoustic meatus toward the tympanic membrane that marks the beginning of the middle ear. The vibration is transmitted through the three main auditory ossicles, malleus, incus, and stapes, where the sound waves are amplified. Ultimately, the sound waves will reach the cochlea where they will be converted into mechanical waves *via* the displacement of the perilymph and endolymph. The last step of this process is triggered by the hair cells in the cochlea that translates this into a nerve impulse, ultimately reaching the central nervous system. In the mouse, the high flexibility of the ossicular joints in the middle ear is responsible for the ability to hear higher frequencies, such as the ones that are characteristic of ultrasonic vocalisations (Navarro et al., 2017b).

Vocal communication plays a crucial role in parental care and especially maternal behaviour in laboratory and wild mice. Mice are altricial species and pups are born hairless, with very limited motor capacity and without thermoregulatory abilities, which makes them completely dependent on maternal care for survival (Weber and Olsson, 2008). USVs are an important component of pup and dam communication, playing a role in maternal bonding, along with odour and tactile stimuli (Nagasawa et al., 2012). Although pups are born deaf and only develop hearing abilities by day 4 or 5 (Weber and Olsson, 2008), they are able to emit vocalisations in a wide range of frequencies that females are able to recognise and respond to in a variety of different maternal behaviours such as, nest building and retrieval (Ehret and Haack, 1981). These include ultrasonic vocalisations, usually associated with isolation (between 30 and 90 kHz), broadband spectrum signals inhibit biting and injury by adults (4–40 kHz) (Haack et al., 1983) and low-frequency calls with a major energy below 10 kHz and a frequency range rarely exceeding 20 kHz. The low-frequency calls are often called wriggling calls and release maternal behaviour, such as licking of the pups (Ehret and Bernecker, 1986).

Females are able to discriminate between sounds produced from pups and sounds emitted by other sources, and show a preference for pup isolation calls when given the choice between these and male USVs or artificial tone bursts with the same frequency properties (Hammerschmidt et al., 2009). It is also known that infant USVs promote cerebral cortex plasticity in mothers, by promoting the activation of certain neuron populations in the auditory cortex in response to ultrasonic and low frequency pup calls (Tasaka et al., 2020).

In this review, we will focus on vocal communication in breeding contexts, covering sexual, parental behaviours and neonatal vocalisations in wild-type and wild derived mice.

## Materials and Methods

All the literature reviewed and included in this review is organised in the table provided as Supplementary Material. Information was extracted and organised into four sections—courtship, copulation, neonatal vocalisations, parental cooperation—and each section is organised into six categories:

• Strain, age, number of animals used
• Detection method
• Testing condition
• Variables measured
• Major findings

The literature included in this review was obtained from PubMed, Web of Science, and Google Scholar using the keywords “mouse,” “vocalisation,” “vocalization,” “ultrasonic vocalisation,” “ultrasonic vocalization,” “courtship,” “neonatal,” and “infant.” The data were collected in a period between April 2020 and November 2021.

Only original research in wild-type or wild-derived mice was selected with no restriction on the date of publication. Exceptions were made for original research using genetically modified lines or artificially induced models that were studied to elucidate crucial mechanisms of vocal communication in mice. Only research published in English or with at least an abstract in English was included.

## Courtship

Historically, vocalisations during heterosexual encounters of mice have been attributed to the male (Sales, 1972; Whitney et al., 1973; Warburton et al., 1989; Barthelemy et al., 2004). Several reasons led to this conclusions, including limitations related to the recording equipment that was used to detect vocalisations or to the way some experiments were conducted which prevented the emission of vocalisations by the female either by devocalisation, by anaesthesia (Whitney et al., 1973) or by replacing the female by olfactory stimulation (Whitney et al., 1974). Nevertheless, Sales (1972) had already noted that ultrasound emission was not exclusive of male mice and that “audible” cries were also detected coming from the female. In more recent research it has been established that females ultrasonically interact with males during courtship displays (Neunuebel et al., 2015) and also produce broadband sounds (Finton et al., 2017).

### Characteristics of Male Courtship Vocalisations

Male mice emit ultrasonic vocalisations with frequencies ranging over 30–110 kHz that meet the criteria of song in that they are composed of different syllable types organised in a non-random temporal sequence (Holy and Guo, 2005). Furthermore, they have an individual signature that can distinguish between individual laboratory mice (Holy and Guo, 2005; Ronald et al., 2020; Melotti et al., 2021) and house mice (Marconi et al., 2020). Male USVs are triggered by the presence of a female (Sales, 1972) and several other related odour stimuli such as female-soiled cage shavings (Whitney et al., 1974), female urine (Nyby et al., 1977), female saliva (Byatt and Nyby, 1986), and female vaginal fluids (Nyby et al., 1977). Nevertheless, the specific chemo signal responsible for this effect has not yet been identified and freezing urine has a deleterious effect on its ability to elicit vocalisations, leading to a decrease in the number of USVs emitted when compared to fresh urine (Hoffmann et al., 2009).

When presented with a novel female, males produced short upward and one-jump syllables soon after the introduction of the female and the number of long syllables with frequency jumps increased approximately 1 min after the introduction of females (Matsumoto and Okanoya, 2016).

### Effects of Age, Social Experience, Relatedness, Genetics

Increasing age was found to negatively affect the number of courtship USVs, with 30 weeks old male mice emitting fewer USVs when compared with younger males (Kanno and Kikusui, 2018). However, when taking into account social experience, the number of USVs is directly related to the past sociosexual experience of the male, and the effect of age can be mitigated by the latter (Kanno and Kikusui, 2018).

The genetic relatedness of the mating partner also seems to modulate male USVs, for instance, when presented with an unrelated female partner, male mice emit more, longer and a higher number of complex USVs when compared to the same interaction with a related female (Nicolakis et al., 2020).

F_1_ hybrids of certain strains have also been demonstrated to have higher calling rate than parent strains, for instance, both F_1_ progeny of C57BL/10Bg.D1-Y and DBA/1Bg have higher calling rates than the parental strains but this is not verified in all strains (Maggio and Whitney, 1986). It has been suggested that there is a directional dominance mode of inheritance of high rate of calling (Hahn et al., 1987).

Cross-fostering experiments with BALB/c and C57BL/6 suggest that adult courtship calls are innate, the calls from fostered males kept the characteristics of the parental strain (Kikusui et al., 2011).

### Effects of Female Oestrous State and Hormonal State

Although males will mount females independently of their oestrous state, they seem to modulate syllable parameters such as dominant frequency, duration and bandwidth according to it. The lowest dominant frequency and highest duration and bandwidth were detected when males were exposed to females in proestrus and the opposite with dioestrus females; intermediate parameters were detected with oestrus females (Hanson and Hurley, 2012). Androgens also influence male USVs: in castrated males, the latency to produce USVs is higher and this is surpassed when males are supplemented with testosterone (Dizinno and Whitney, 1977).

### Effects of Social Context

Social status seems to affect the amount of USVs produced by male mice when interacting with a female: dominant males emit more 70-kHz vocalisations when compared to subordinates, in a courtship context (Nyby et al., 1976). Furthermore, an “audience effect” was described by Seagraves et al. (2016), in that male mice modified their call rate, acoustic structure and syllable complexity in response to the presence of male body odour and an anaesthetised male audience. Interestingly, this effect was only observed in the simultaneous presence of male odour and an anaesthetised male and not when the experimental subject was exposed to only male odour or only male USVs.

### Characteristics of Female Vocalisations During Courtship Displays

Females produce two types of vocalisations during interaction with males, USVs, and audible squeaks or broadband vocalisations (BBVs) (Lupanova and Egorova, 2015; Neunuebel et al., 2015; Ronald et al., 2020). They appear to have two distinctive functions: USVs are mainly produced during pursuit by males and when in close proximity with males (Neunuebel et al., 2015) and BBVs are often produced in accompanying behaviours such as kicking or lunging at males, often signalling rejection (Sugimoto et al., 2011). Ronald et al. (2020) investigated this further by exposing male mice to female urine with USVs or BBVs playback and found that male mice adjust their vocal courtship according to the type of vocalisation emitted by females: male USVs production was highest when exposed to female urine and USVs and lowest when they were exposed only to female urine, BBVs or the combination of BBVs and female urine, with no differences between all these experimental conditions. BBVs were used in this case because they are associated with rejection from the female. This also shows that olfactory stimuli provide context for female vocalisations.

### Female Preferences and Reproductive Success

When exposed to male ultrasonic vocalisations, females seem to be attracted to them independently of their oestrous status as shown by Hammerschmidt et al. (2009). Furthermore, females prefer to interact with vocally intact males or surgically devocalised males with a playback of artificial 70 kHz tones when compared to devocalised males (Pomerantz et al., 1983). When given the choice between a compartment with a playback of male USVs against a silent compartment, females spent more time in the first. Such a preference was not found when the USVs were obtained from mouse pups (isolation calls) or artificial tone bursts of constant frequency between 70 and 80 kHz (Hammerschmidt et al., 2009).

Females also seem to have a preference for complex versus simple male vocalisations, as shown by Chabout et al. (2015) in a Y-maze choice test paradigm, this indicates that the female response to male courtship USVs is multidimensional, depending on the characteristics of male USVs.

Furthermore, female mice prefer male USV playback from unfamiliar non-kin compared to that from familiar siblings which indicates that females are able to discriminate between familiar siblings and unfamiliar non-siblings by their USVs. This occurs in both laboratory mice (Asaba et al., 2014) and wild-derived mice (Musolf et al., 2010). One interesting point to note is that this preference is not genetically determined. This was shown in cross-fostering experiments, where C57BL/6 females raised by BALB/c foster mice showed a preference for male USVs produced by C57BL/6 males. In contrast, C57BL/6 females that were raised by C57BL/6 parents showed a preference for male BALB/c USVs. Furthermore, song preference disappeared when females were raised in fatherless conditions. This demonstrates that the environment during development plays an important role in the subsequent reproductive life of females (Asaba et al., 2014).

Less is known about the relationship between male USVs and subsequent reproductive success, nevertheless, Nicolakis et al. (2020) described a negative correlation between the mean number and length of vocalisations with the latency of the pairs first litter.

## Copulation

In seminal work, Sales (1972) first described the occurrence of ultrasonic calls during mating behaviour in laboratory mice in three different strains, specifically during investigative behaviour of the female by the male (sniffing) and during mounting and intromission. This work detected 40 kHz vocalisations during mating (Sales, 1972); however subsequent investigations focused on 70 kHz vocalisations. It was not until over two decades later that White et al. (1998) first documented the occurrence of both 40 and 70 kHz vocalisations during copulation. These two different vocalisations occur at different times, 70 kHz are emitted throughout the precopulatory and copulatory periods and are interspersed with 40 kHz calls that are seen most often in the later phases of a copulatory sequence (Sales, 1972; White et al., 1998). In addition, spectrographic analysis of USVs emitted during mounting revealed that males produced longer and more complex syllables with harmonics during mounting behaviour (Matsumoto and Okanoya, 2016).

## Neonatal Vocalisations

Neonatal mice are capable of producing vocalisations in a wide range of frequencies. Three different call types have been described in infant mice: distress calls in ultrasonic range, broadband or “pain calls” and low-frequency or “wriggling calls” (Zippelius and Schleidt, 1956; Ehret and Bernecker, 1986). Each of these are involved in mother-offspring communication and recognition and play different roles in eliciting maternal behaviour (Ehret and Haack, 1981; Ehret and Bernecker, 1986).

Due to the altricial nature of mouse pups (Weber and Olsson, 2008), mother-infant communication plays a crucial role in survival, and neonatal vocalisations are central in infant-mother communication, as vocalisation is virtually the only mean to convey information from the pup to the dam.

### Ultrasonic Calls of Infant Mice

Isolation ultrasonic calls in neonatal mice were first described as “Pfeifen des Verlassenseins” (“whistles of abandonment”) by Zippelius and Schleidt (1956). USVs emitted by neonatal mice are whistle-like sounds characterised by frequencies ranging between 30 and 90 kHz, duration of 10–200 ms, and sound pressures of 60–100 dB (Branchi et al., 2001).

#### Development of Ultrasonic Vocalisations in Infant Mice

The temporal organisation is closely related to landmark developmental stages of neonates (Elwood and Keeling, 1982). The variations in calling rate mirror the stages of development of homoeothermy in mice which can be divided into three different phases: from 2 to 5 days of age, there is near poikilothermy, this is followed by a second phase, from 8 to 15 days of age, with an increasing ability to produce body heat and a final stage from 18 days until 30 days of age where homeothermy is achieved (Nagy, 1993). Call rate increases during the first 6–7 days of age, reaches a peak at 8 days and then decreases gradually until the end of the second week after birth (Hahn et al., 1998; Thornton et al., 2005). Other developmental milestones have been associated with changes in calling rate, Noirot (1966) described that calling rate increased until D4 which coincided with the opening of the ears and decreased almost to zero on the day which the opening of eyes was recorded.

During this period, call length and frequency range characteristics also vary, declining linearly over time; on the other hand, other frequency measures (beginning and ending frequency, highest and lowest frequency of call) increased with age (Hahn et al., 1998). In addition, regarding the spectrographic characteristics of USVs, there is an increased proportion of harmonic calls over the initial 13 days (Grimsley et al., 2011).

#### Influence of Environmental Factors

The intensity and calling rate of ultrasonic vocalisations is inversely related to the environmental temperature at which the pups are exposed (Okon, 1970a; Sales and Skinner, 1979). Furthermore, in experimental conditions, the day that pups cease to call is related to the environmental temperature, for instance, at 33°C isolated pups ceased to call at 11 days of age and, at 2–3°C, calls ceased at 19 days of age.

Other factors that elicit ultrasounds in neonatal mice are tactile stimuli (handling, loss of balance, retrieval by the mother) (Okon, 1970b; Henessy et al., 1980; Hahn and Schanz, 2002), and odour cues (Marchlewska-Koj et al., 1999).

Cross-fostering experiments also reveal that call features are primarily dependent on the genotype of the pups and not on early environment, with the exception of call amplitude that seems to be dependent on the mother genotype (Wohr et al., 2008).

Social context also influences the characteristics of infant USVs. Odour seems to play an important role on how mouse neonates perceive the external environment in the early neonatal period, olfaction is one of the best developed senses in neonatal mice (Walz et al., 2006) and this becomes even more evident due to the fact that odour is the main cue for nipple grasping and, thus, successful suckling (Al Ain et al., 2013) which is crucial for neonatal survival.

Pups on post-natal day 8 cease to call when exposed to the odours of unfamiliar males (Branchi et al., 1998). But, an increase calling rate was reported on pups aged between 2–10 days old when exposed to odours from non-infanticidal males, defined as unfamiliar males that either ignored or showed parental toward the pup and a clear increase in call rate when the same pup was exposed to urine of non-infanticial male followed by urine of an infanticidal male (Santucci et al., 1994). Furthermore, infant mice seem to be able to distinguish genotype, Marchlewska-Koj et al. (1999) reported an increase in the calling rate of CBA pups when exposed to bedding from lactating females of a different genetic background (C57BL/6J), and this occurred even when CBA pups were fostered by C57BL/6J females. Furthermore, Kapusta and Szentgyorgyi (2004) observed an increase in calling rate when CBA pups were exposed to bedding from two unrelated strains (C57BL and DBA).

Environmental conditions have also been found to influence the spectrographic characteristics of ultrasonic vocalisations in neonatal mice, as described by Branchi et al. (1998). Eight days old CD-1 pups of both sexes exposed to five different conditions (odour from the nest, social isolation, low-temperature isolation, tactile stimuli, or odour from conspecific unfamiliar adult mice) emitted differently shaped USVs in each of the situations. More frequency steps were observed in low frequency range calls by animals exposed to low temperature isolation or to male odour. Isolated pups produced more frequency steps calls in medium frequency range calls. Pups exposed to low temperature isolation emitted a higher number of modulated frequency signals in high frequency range calls.

#### Strain and Genetic Influences

Pups of different strains of mice present different characteristics of distress calls. For instance, C57BL produces less number of ultrasounds than BALB/c and SEC strains and the peak of ultrasound emission also differs between this strains with C57 reaching a peak sooner (D2–D4) than SEC strain (D4-8) with BALB/c reaching a peak in an intermediate period (D3–D7), all presenting a fundamental frequency of 60–70 kHz (Robinson and D’Udine, 1982). It is interesting to note that SEC strain carries the short ear mutation (Bmp5*^se^*) which is related to the occurrence of short, slightly ruffled external ears due to defective cartilage framework and an abnormal skeleton with numerous local defects in homozygotes and this can potentially have an impact on the evolution of vocalisations in this strain. Bell et al. (1972) also reported rates of calling higher in the BALB/cJ strain compared to C57BL/6J and C3H/HeJ at 3 days of age but this pattern changes across time, with C3H/HeJ calling more frequently at 9 days of age when compared to the other two strains, frequency and call duration also differ among the three strains, all with a modal peak frequency situated between 70 and 80 kHz.

Furthermore, between post-natal days 2–12, C57BL/6J (B6), 129 × 1 and FVB/NJ produced a wide repertoire of calls, which included high numbers of frequency steps and complex USVs. In more detail, B6 pups emitted more downward, chevron and short calls and, less two-syllable calls than the C57BL/6J, 129 × 1 and FVB/NJ. When comparing the calls emitted by C57BL/6J, 129 × 1 and FVB/NJ with the ones emitted by outbred CD-1 pups at the same age—8 days old—the latter produced a higher percentage of frequency steps and complex calls but low numbers of flat, short and complex calls (Branchi et al., 1998; Scattoni et al., 2008).

Sex can also have an influence on call characteristics (call rate, duration, and frequency) of mouse pups, female pups emit fewer calls with a lower frequency and length when compared to males (Hahn et al., 1998).

#### Effects on Maternal Behaviour

Ultrasonic vocalisations of infant mice affect maternal behaviour in several ways. USVs emitted by mouse pups, stimulate maternal retrieval and nest building (Zippelius and Schleidt, 1956; Noirot, 1964, 1966; Smotherman et al., 1974). And, as Ehret and Haack (1984) suggests they seem to have two major functions, to release maternal behaviour, such as searching for a lost pup, and to serve as a cue for the pup’s location.

Infant USVs seem to play a part in offspring recognition (Mogi et al., 2017) but, interestingly, its role and importance seem to differ between strains. In experiments performed in ICR mice, infant USVs seem to be enough stimulus for maternal recognition (Uematsu et al., 2007; Okabe et al., 2010) but C57BL/6 dams require simultaneous presentation with pup odour (Okabe et al., 2013).

Furthermore, ICR females are able to discriminate between own and alien pups through their USVs (Mogi et al., 2017).

Interestingly, maternal responsiveness in C57BL/6 mice is negatively correlated with pup USV calling rate (D’Amato et al., 2005).

Further studies were performed in genetically deaf mice to elucidate the importance of vocal communication in the mother-offspring interaction, D’Amato and Populin (1987) performed observations in deaf females rearing deaf pups and cross-foster experiments with normal hearing mothers and deaf pups and vice-versa. The results indicate that maternal behaviour was not affected by the deafness of the mother when taking care of her own pups. Females seemed to compensate deafness by increasing activity levels but neither nest building or pup retrieval were affected. In contrast, cross-fostering of deaf pups that emitted less calls with normal hearing mothers seemed to decrease maternal care, possibly due to the fact that the lesser number of calls was interpreted as no need for maternal care.

### Broadband and Low Frequency Calls of Infant Mice

Two other types of vocalisations have been described in infant mice: broadband spectrum and low frequency calls. Broadband spectrum signals inhibit biting and injury by adults. These can be emitted post-partum, when the dam is cleaning away foetal membranes, and are characterised by a frequency between 4 and 40 kHz (Haack et al., 1983). Low-frequency wriggling calls are characterised by having a major energy below 10 kHz and a frequency range rarely exceeding 20 kHz. These stimulate maternal behaviour, such as licking of the pups, and can be emitted when pups press to reach their mother’s teats, when the litter is alone inside the nest or when pups older than 5 days crawl over each other (Ehret and Bernecker, 1986).

## Parental Cooperation

A new function for ultrasonic vocalisations in parental cooperation has been described. A 38 kHz USV emitted by female ICR mice was detected when dams were separated from pups, defined as pairmate-dependent retrieval as it seems to elicit retrieval of pups by the father-(Liu et al., 2013). This vocalisation was only detected in ICR mice and not in C57BL/6J or BALB/c (Liang et al., 2014), and seems to be associated with the activation of aromatase which synthetises oestrogen from androgen in several brain regions of male mice (Akther et al., 2015).

## Conclusion and Future Perspectives

In recent years, research into mouse vocal communication has gained a lot of interest. Neonatal USVs are very well-characterised in the most common mouse strains and have been an invaluable tool in neuroscience research (Premoli et al., 2021), for instance, in neurodevelopmental disorders such as autism (Scattoni et al., 2008, 2011; Wohr et al., 2011). But some aspects of vocal communication in breeding contexts are still poorly understood. Literature on neonatal low-frequency vocalisations is scarce and information about vocal communication in established social groups is lacking.

Most research in vocal communication has been performed in laboratory conditions due to technical constraints. Detection of sounds from these animals is usually done in highly controlled environments to avoid contamination by other sounds and requires highly specialised equipment. Social interactions are usually brief and there is a lack of data on vocal communication between established groups. So, although there is published data on laboratory and wild-derived mice, no studies were performed in naturalistic conditions. Nevertheless, efforts have been made to surpass this, and de Chaumont et al. (2021) have recently developed a sophisticated apparatus that allows the recording of video and sounds during long periods of time which can provide novel insights into vocal communication and its correlation with behaviour.

In laboratory animal facilities, breeding is mainly performed in trios where two females are kept with the male continuously. This allows females to nest communally and also permits the occurrence of post-partum mating. Communal nesting occurs in wild mice and poses several advantages such as sharing of rearing tasks and, even, communal nursing (Weber and Olsson, 2008). As such, it would be expected that females communicate vocally in these contexts and that this would have an impact on maternal care and pup survival. But the current literature only addresses vocal communication in same sex or heterosexual encounters (Warren et al., 2018, 2020; Sasaki et al., 2020; de Chaumont et al., 2021). It is know that females vocalise in same-sex encounters and produce more complex USVs in these situations (Matsumoto and Okanoya, 2018) but it is still unknown how this would be affected by the presence of a male.

Further studies are needed to understand the social dynamics between females in a breeding trio and the potential impact on pup survival. The development of home cage monitoring systems with the ability to detect vocalisations will be a useful tool to fill this gap of knowledge.

Laboratory mouse strains have different profiles with regards to their hearing range and susceptibility for hearing loss (Davis et al., 2001). The most prominent example is the well-characterised age-related hearing loss in C57BL/6J mice which is caused by a mutation in cadherin 23 (Noben-Trauth et al., 2003). Hearing capabilities of this strain start to decline as soon as 3 months old; higher frequencies are initially affected, and by 1 year of age, these mice present a severe-to-profound high frequency hearing loss (Ison et al., 2007). Furthermore, sex differences have been reported with females presenting a more severe degree of hearing loss when compared to males of the same age (Henry, 2002). On the other hand, CBA/CaJ mice maintain relatively stable hearing over age, making them a preferred strain to use in vocal communication studies (Erway et al., 1993). It is not clear how gradual hearing loss can impact mouse vocal communication, and especially, communication between dam and pup since most studies were performed in genetically modified strains which are deaf since birth (D’Amato et al., 2005) or using artificially induced deafness (Ehret and Bernecker, 1986). More research is needed to elucidate on the impact of hearing loss in vocal communication in a breeding context in laboratory strains.

The spectrographic analysis of USVs in mouse communication is also one of the aspects that has gained a lot of research interest in the last decade. Mice produce different shapes of USVs (see Table 1) that can potentially deliver different messages to the receiver. Spectographic analysis has been performed to gain insight into the social deficits of autism models, such as the BTBR strain (Scattoni et al., 2008, 2011). Several authors hypothesised about the meaning of these different types of USVs that were first described by Scattoni et al. (2008), Branchi et al. (1998) suggested that, since high frequency sounds travel for a short distance and low frequency sounds for a longer distance, the first could serve to be used in intraspecific communication (i.e., intralitter, nest area range) while the latter would be used in other types of intraspecific communication, such as out of the nest range. Grimsley et al. (2011) also suggested that the changing proportions of different syllable types could be used as cues for mothers to distinguish different aged pups. Matsumoto and Okanoya (2018) hypothesised that since females interacting in same-sex groups produce more complex calls and males tend to produce simple calls, these could be useful to maintaining group structure. Further research is needed to elucidate the context and behavioural meaning of different syllable types.

## Author Contributions

SC-P performed the literature research and wrote the first draft. SC-P and IO designed the manuscript. All authors discussed, provided input, contributed to the article, and approved the final manuscript.

## Conflict of Interest

The authors declare that the research was conducted in the absence of any commercial or financial relationships that could be construed as a potential conflict of interest.

## Publisher’s Note

All claims expressed in this article are solely those of the authors and do not necessarily represent those of their affiliated organizations, or those of the publisher, the editors and the reviewers. Any product that may be evaluated in this article, or claim that may be made by its manufacturer, is not guaranteed or endorsed by the publisher.

## Footnotes

1 We will adhere to the established terminology and refer to vocalisations beyond the human hearing range as ultrasonic; however this anthropocentric terminology is questionable when referring to vocalisations in species of non-human animals with a different hearing range.
