# Supplementary material for: From Mating to Milk Access: A Review of Reproductive Vocal Communication in Mice
Source: Front Behav Neurosci. 2022 Mar 28;16:833168. doi: 10.3389/fnbeh.2022.833168 (PMC8995852; doi:10.3389/fnbeh.2022.833168)
Supplement: Supplementary file 1 [file Table_1.PDF]

Supplementary Table 1 – Literature reviewed

Index

COURTSHIP 2

COPULATION 56

NEONATAL VOCALIZATIONS 59

PARENTAL COOPERATION 105

REFERENCES 109

Supplementary Table 1 – Literature reviewed

Courtship

Author	Strain, Age, Number	Detection Method	Testing Condition	Housing conditions	Variables measured	Major findings
(Asaba et al., 2014)	C57BL/6 and BALB/ females 7-35 weeks old<br><br>B6, BALB, KJR and ICR Males 20-30 weeks old	Ultrasound recording was performed using a condenser microphone (UltraSoundGate CM16/CMPA, Avisoft Bioacoustics, Berlin, Germany) as previously reported. When recording songs from adult males, the microphone was placed beside a cage (12x20x11 cm) with 6-cm diameter holes and covered with a 0.5- cm wide mesh.	**Cross-fostering and fatherless condition**<br>A male and female of the same strain were pair-housed in a cage for breeding.<br>Litters were cross-fostered to parents of other mouse strains (B6 or BALB) when pups were born at the same examined time 6 to 8h time period in both parental strains.<br>Fatherless females were raised by a pregnant female singly-housed.<br><br>**Two-choice preference test**<br>Females were exposed to male songs from B6 and BALB mice simultaneously (for a 5 min period) in a test cage (inside a soundproof chamber) divided in 4 zones:<br>- Neutral zone (the undivided section of the test cage)<br>- Mesh zone (mice searching	Same sex groups (2-5 mice).<br>Cage dimensions: (17.5624.5612 .5 cm)	**Two choice preference test**<br>- Number of entries into the sound compartment<br>- Duration of stay in each speaker zone and middle zone<br>Duration of searching on the mesh	**Strain-specific character of male songs**<br>The B6 song contained more ‘‘One jump’’ and ‘‘More jump’’ syllables, whereas the BALB song contained more ‘‘Harmonics’’ compared to the other strains. In addition, the KJR song contained more ‘‘Flat’’ , and the ICR song had more ‘‘Downward’’ syllables. The songs produced by males of these 4 strains were different from one another in song profile: peak frequency, syllable interval and syllable duration and were categorized in two different groups (ICBR + BALB and KJR +B6).<br><br>**Female mice prefer the songs of different strains when stimulated by male sexual chemosignals**<br>B6 and BALB females showed preferences for songs produced by males of other strains in the dioestrus stage, when stimulated by male sexual cues (soiled bedding).<br>Following exposure to ESP1, females from both strains of mice significantly preferred songs produced by males of a different strain.<br><br>**Song preference is based on dissimilarity of song character**<br>B6 females preferred ICR songs over B6 songs, but not KJR songs. BALB females preferred KJR songs over BALB songs, but not ICR songs.<br><br>**Song preference was sharpened by the father’s presence during development**<br>BALB fosterers preferred BALB song and B6 fosterers preferred B6 song. In addition, song preference disappeared when females were raised in a fatherless condition.

			and in contact with the wire mesh)<br>- Speaker zone (an area 0-10 cm from the mesh)<br>- Middle zone (an area 10-20 cm from the mesh.<br><br>To present mouse chemical signal cues, male-soiled bedding or pheromone (ESP1) was placed into the Neutral zone to increase the female's sexual arousal before starting the 5-min test. Female-soiled bedding was used as a control stimulus. The male- and female-soiled bedding was a mixture of 2 g each from adult B6 and BALB/c males and females respectively (3 mice per strain). Recombinant ESP1 (20 mg) in Tris buffer was transfused onto a piece of cotton (30 mg) and dried for 1 h before exposure. Each female was exposed to the soiled bedding for 15 min, or to ESP1 for 30 min, before initiation of the test.			

			**Estrus cycle and hormonal treatments**<br>Estrus cycle was determined by examination of vaginal smears.			
**Author**	**Strain, Age, Number**	**Detection Method**	**Testing Condition**	**Housing conditions**	**Variables measured**	**Major findings**
(Barthelemy et al., 2004)	BALB/c<br>8-9 weeks old<br><br>6 females<br>8 males	Mice calls were recorded using a D940 ultrasound detector from Pettersson Elektronik (Uppsala, Sweden) positioned 10 cm above the cage. The setting of the detector was frequency division, i.e. mice vocalizations are changed with a constant factor of 10 so that the frequency is in the human auditory range and any activity over the entire frequency range 1– 110 kHz (ultrasonic and	Each of the six females was paired three times with different males: during a dioestrus phase, an oestrus phase and a proestrus phase. Two tests involving the same female were separated by at least one complete sexual cycle. Each test was performed under dim red light at the beginning of the dark phase by introducing the female into the male's cage (transparent polypropylene cage - 250x160x136 mm - with a metal wire lid) The animals' sexual behaviour was observed and vocalizations were recorded for 5 min.	Single housed for 7 days prior to the experiment and housed in small propylene cages (250x160x136 mm).	Recorded behaviours<br>1. Approach: one mouse approaches the other from at least 10 cm away.<br>2. Break-off; one mouse orients away from the partner.<br>3. Sniff: one mouse sniffs or licks any part of its partner's body.<br>4. Trail: the male runs closely behind a running female. When the female stops, a trailing male will usually mount.<br>5. Mount: the male approaches the female from the rear, grasps the female's sides with its forepaws,	<u>**Description of vocalizations**</u><br>3 types of vocalizations were recorded:<br>- A: partially audible to humans as high-pitched soft whistles. Frequency ranged from 1 to 40 (±8) kHZ and duration averaged 50 (±15 ms). Emission rate was 4-5 calls/s in 10-15 call sequences.<br>- B: Frequency range from 50 (±5) to 70 (±) kHz and average duration 70 (±20) ms.<br>- C: Frequency modulation restrained to two 20kHz intervals. The first group consisted of a modulation between 30 and 50(±6) kHz.The second group had a rapid modulation of frequency between 50 and 70(±10) kHz. Call duration averaged 100(±20) ms. Rate of emission was 8 calls/s.<br><br>"A" vocalizations were mainly associated with approach and break-off behaviours. Also emitted during trailing and mounting behaviours along with "B" calls.<br>"B" calls were emitted during pre-ejaculation and during mounting.<br>"C" calls were emitted during coitus.<br><br>During trailing, "A" and "B" calls represented 70 and 30 (±2)% of the vocalizations and were emitted at a rate of 6-8 calls/s in 10-20 call sequences.<br><br><u>**Vocalization emission pattern**</u><br>- Females paired in dioestrus: low receptivity to male courtship and all mounting attempts were rejected. The

		audible) could be continuously monitored.			and palpates the female flanks.<br>6. Intromission: an intromission is suspected when the mount lasts more than 5 s. An ejaculation is suspected when a long-lasting intromission ends with a slow dismount.<br><br>USVs parameters:<br>- Frequency (minimum, maximum)<br>- Call rate<br>- Call duration	amount of pre-ejaculatory call emissions remained stable for the 5 min of recording but decreased slightly during the last minute.<br>- Females paired in estrus: High receptivity to courtship but never accepted intromission. The quantity of pre-ejaculatory calls emitted reached a mean of 17 calls/5 s during the first minute of recording, and then rapidly decreased twofold for the last 4 min.<br>- Females paired in proestrus: Highly receptive to courtship. The quantity of pre-ejaculatory call emissions remained stable for the 5 min of recording and most of the calls were of the B-type.
**Author**	**Strain, Age, Number**	**Detection Method**	**Testing Condition**	**Housing conditions**	**Variables measured**	**Major findings**
(Byatt and Nyby, 1986)	B6AKF1/J<br>CD-1<br>Swiss Webster<br><br>121 male B6AKF1/J (156-319 days old)<br>70 female B6AKF1/J (59-319 days old)<br>8 female Swiss Webster (50 days old)	Ultrasonic vocalizations were detected with a QMC S100 bat detector tuned to 70 kHz with the microphone centered 25 cm above the floor of the test chamber	Experiment I<br>- Subject males were exposed to facial, vaginal and salivary odors of females to elicit ultrasonic vocalizations. Stimulus donor females were either ovariectomized or hypophysectomised.	Animals were housed in wire-topped, translucent plastic cages (13 x 17 x 28 cm) with wood chip bedding. The subject's home cage also served as the test chamber. All animals were maintained on a 12: 12light:	Call rate	Experiment I<br>- The stimuli from the ovariectomized females generally being better for eliciting vocalizations than the stimuli from the hypophysectomized females.<br>- Neither the effects of body site nor the interaction of body site with type of surgery were<br>- significant. Further analysis indicated that stimuli obtained from ovariectomized females were significantly better at eliciting vocalizations than those from hypophysectomized females for the facial stimuli (<br>- and for the salivary stimuli but not for the vaginal stimuli.<br><br>Experiment II<br>- Long-term ovariectomy (9 months) had significantly different effects upon the three stimuli depicted. Urinary stimuli elicited more vocalizations than did the two

	14 female CD-1 (58 days old)		Experiment II<br>- Subject males were exposed to female saliva either 8 months or 9 months after ovariectomy. Or urine of females 9 months after ovariectomy. In both conditions, male vocalizations in response to the stimulus were assessed.	dark cycle with ad libitum food and water.		salivary stimuli while the two salivary stimuli did not differ.
**Author**	**Strain, Age, Number**	**Detection Method**	**Testing Condition**	**Housing conditions**	**Variables measured**	**Major findings**
(Chabout et al., 2015)	Males (n=12; 8 weeks old) and females (n=22; 7-15 weeks old) of the B6D2F1/J<br><br>8 weeks old	Sounds were recorded with UltraSoundGate CM16/CMPA ultrasound microphones suspended over the centre of each cage in the recording box, high enough so that the receiving angle of the microphone covered the whole area of the test cage. The microphones	<u>Behavioural paradigm</u><br>Sexually experienced males were exposed to one of the following different stimuli: (1) fresh urine collected from at least two different females (UR) or males (URM) from two distinct cages (and mixed) within minutes of exposure on a urine-dipped cotton tip placed inside the male's cage; (2) awake and behaving adult female (FE); (3) an anesthetized adult female (AF); and (4) an anesthetized adult male (AM).	Before experiments, all mice were group housed (4–5 per cage) and kept on a 12-h light/dark cycle, and received ad-libitum food and water.	Male USVs:<br>- Amplitude<br>- Syllable duration (ms)<br>- Pitch (frequency mean)<br>- Bandwidth<br>- Spectral purity<br>- Sequence length<br>- Number of syllables /min	- Fresh female urine (UR) collected within 2 min of presentation to a male elicited the highest number of syllables and reliable singing from all males. By contrast, when males were presented with fresh male urine (URM) collected within 2 min of presentation, there was very little to no singing, depending on animal<br>- All male mice sang in response to a live sexually experienced female (FE) and at comparable levels with fresh female urine.<br>- Males still sang considerable amounts, although some less, with an anesthetized female, demonstrating that reciprocal social behavior was not necessary for them to sing robust amounts of song.<br>- In all contexts, male mice produced the simpler syllable type without pitch jumps, "s," more often than all other types. However, in the presence of fresh female urine they produced significantly less "s" type, and more down "d" and multiple "m" pitch jump types. The relative proportion of the up "u" pitch jump syllable was similar across contexts.

		were connected to a multichannel ultrasound recording interface Ultrasound Gate 416H, which was itself plugged into a computer equipped with Avisoft Recorder USG software v4.2.18 (Sampling frequency: 250 kHz; FFT-length: 1024 points; 16-bits). All recording hardware and software were from Avisoft Bioacoustics(Berlin, Germany).	Each male was exposed to the same stimuli for three consecutive days and this was repeated for four weeks with different stimuli.<br><br>Playback Behavioral Experiment<br>Oestrus was induced in females by 3 days exposure to male mice in a cage that allowed for odour and visual stimulus but no physical contact.<br>Females were tested in a Y-maze with two speakers placed in the extremity of two arms of the Y-maze. one speaker on one side played a male song previously recorded during the UR context and the other speaker simultaneously played a song from of the same male from the FE context, for 5 min.		Playback Behavioural Experiment:<br>- Time spent in each arm	- Males produced syllables up to four times louder in the urine condition than in all the other conditions.<br>- Males produced their syllables with longer duration and higher in the urine and awake female context relative to other context, with the shortest duration in the anesthetized male context.<br>- Songs emitted in the awake female condition are sharper than in all the other contexts.<br>- Consistent with the amount of singing, males emitted longer sequences when exposed to an awake female, followed by an anesthetized female and urine conditions.<br>- In each of the FE, AF, and AM contexts, the males produced successively fewer sequences with 2 or more "m" syllables.<br>- The female urine stimulated songs (from UR) from three males contained a majority of "d," "u," and "m" syllables, whereas their awake and behaving or anes- thetized female-stimulated songs (from FE and AF) contained mostly simple "s" syllables.<br>- Nearly all females spent more time (on average ∼30% more) in the arm which had the complex stimu- lated urine song from all three males than in the arm with the simple song that was played simultaneously
**Author**	**Strain, Age, Number**	**Detection Method**	**Testing Condition**	**Housing conditions**	**Variables measured**	**Major findings**
(Dizinno and Whitney, 1977)	B6DBAF1/J (24 males)<br>DBAB6F1/J (8 males)	A Holgate ultrasonic receiver mk IV, set to a centre frequency of 70 kHz, converted ultrasounds to audible sounds	Sixteen males were assigned to a castration condition and sixteen to a sham condition. Within these two conditions, one half were assigned to a testosterone treatment condition (TP)	Animals were housed with same-sex littermates in 29x18x13 cm transparent plastic cages. After surgery,	Latency to initial ultrasound	- Castration clearly increased the latency to production of ultrasounds from adult males, and a subsequent testosterone propionate injection decreased the latency.<br>- During the preinjection phase, ultrasounds were produced with a median latency of 19.09 set by the sham males. The corresponding latency of 183.17 set for the castrated males was significantly greater.

		which could be monitored by the experimenter. Timing was done with two electric timers accurate to the nearest 0.01 sec	and one half to a peanut oil control condition (OIL), resulting in eight males in each of the four groups (castration-TP, castration-OIL, sham-TP and sham-OIL).<br>Testing was conducted in two phases: preinjection phase and postinjection phase.<br>The stimulus female was then taken from her home cage, and the test began by placing her in the test cage as far from the male as possible.<br>Latency to initial ultrasound was recorded, and the test was terminated after 5 min. A latency of 300 set was recorded for subjects who failed to emit ul- trasounds during the test.	they were single-housed in the same cages.<br>Food and water was provided *ad libitum* and a 12-12 light-dark cycle was maintained throughout the experiment.<br><br>Females were housed with same-sex littermates in 10x24x13 cm stainless steel cages.		- No significant differences in latency were detected between the castrate-TP group and the castrate-OIL group, or between the sham-TP and sham-OIL groups for the preinjection phase.<br>- Following injections, both sham-TP and castrate-TP males significantly reduced their latencies to first ultrasonic emission.
**Author**	**Strain, Age, Number**	**Detection Method**	**Testing Condition**	**Housing conditions**	**Variables measured**	**Major findings**
(Finton et al., 2017)	CBA/J<br><br>Number of females=13<br>Number of males= 13+13<br><br>Aged 7-8 weeks	Both audio and video recordings were collected via a micro- phone and a video camera positioned above the cage, in the case of females in their	Each female subject and male partner mouse participated in three unique social interactions, with one interaction per day and a novel partner for each interaction.<br>Before the interaction, fe- males in their home cages were placed in the	Mice were housed in standard plastic caging for laboratory mice (28.5 x 17.5 cm and 12.5 cm tall), with pine bedding and supplemental	Nonvocal behaviours:<br>- Rejection<br>- Mounting<br>- Escape<br><br>Analysis of vocalizations<br><br>BBVS:<br>- Number	- 10 female CBA/J mice produces a total of 6325 human-audible broadband vocalizations (BBVs) during opposite-sex social encounters with males in the female home cage.<br>- The mean duration of all BBVs was 75.95+- 0.3254ms and the mean fundamental frequency was 3804.14+-0.324Hz. 51.8% incorporated at least one nonlinear segment, characterized by abrupt transitions into either (1) a doubling or tripling of the number of harmonics (subharmonics) or of (2) noise-like structure (deterministic chaos).

		home cages. Vocalizations were recorded with 16- bit resolution with a condenser microphone (CM16/CMPA; Avisoft Bioacoustics, Berlin, Germany; 200kHz maximum range) and sound card (250 kHz sample rate, UltraSoundGate 116 Hb, Avisoft Bioacoustics). Nonvocal behaviours were recorded with a CCD video camera (30 frames/s), Q-see 4-channel DVR PCI video capture card and SuperDVR software (Q-see, Digital Peripheral Solutions, Inc., Anaheim, CA, U.S.A.).	recording chamber for 5 min. After habituation, a male of 7e8 weeks of age was added to the female's home cage and the interaction began. At the end of a 20 min interaction, the male was removed from the female's cage and returned to his home cage. From the 10 females and 10 males interacting in the home cages of females, a total of 30 unique male-female pairings were recorded.	nesting material	- Duration<br>- Harmonic-to-noise ratio (HNR)<br>- Fundamental frequency<br><br>USVS:<br>- Number<br>- Categorization – 50Hz harmonic calls and other calls	- The number of BBVs emitted during encounters with males was significantly correlated with the number of male-directed rejection behaviours in the same encounter.<br>- BBV timing corresponded to female climbing, as demonstrated in event-triggered averages anchored to incidences of female climbing. In the 40 s before females escaped up the cage sides, BBV number steadily increased, culminating in female escape.<br>- The duration of BBVs overall was different among females.<br>- There was no significant effect of oestrous state on BBV rate or fundamental frequency.<br>- The absolute duration of linear segments of BBVs also did not differ significantly between oestrus and dioestrus.<br>- Females produced BBVs with nonlinear segments of significantly longer duration during oestrus (28.43 ± 4.47 ms) than during dioestrus (14.65 ± 2.01 ms).<br>- Patterns of female BBVs over time varied with a key event, whether mounting of females by males occurred.<br>- More BBVs were emitted by females that were not mounted, and at early time points.<br>- For interactions in which mounting occurred, there was a close correspondence in time between BBVs and 50 kHz harmonic USVs.
**Author**	**Strain, Age, Number**	**Detection Method**	**Testing Condition**	**Housing conditions**	**Variables measured**	**Major findings**

(Hammerschmidt et al., 2009)	32 female C57BL/6NCrl 12-15 wks at the beginning of testing	The microphone (UltraSoundGate CM16) and interface (UltraSoundGate 116) with pre-amplifier and A/D converter were connected to a notebook (ultrasound hardware and software: Avisoft Bioacoustics, Berlin, Germany). The microphone was placed above the middle compartment of the place preference box.	**<u>Experimental apparatus</u>**<br>Mice were tested for place preference in a rectangular, 3 chambered box. Each chamber was 20 cm x 40 cm x 22 cm in size. Dividing walls were made of clear Plexiglass with openings (10cm) in the middle allowing access into each chamber and proper propagation of sound. Loudspeakers were situated in front of the outer walls of the peripheral chambers, which had corresponding circular openings with stainless-steel mesh inserts for free propagation of sound.<br><br>**<u>Behavioural testing</u>**<br>Females were tested during the oestrus or dioestrus phase (each female was tested twice with 2-3 days between trials).<br>3 different types of playback sounds were broadcast for 3 separate groups of females:<br>1) male mouse song (n=10)	No information provided	Time in sound chamber	In the first presentation, females spent significantly more time at the side from which male mouse songs were broadcast than in the control conditions.<br>No preference was observed in the second presentation.<br>Responses to other playback sounds remained independent of presentation order.<br>No significant differences were found whether females were in oestrus phase or not.

			2) pup isolation calls (n=11)<br>3) whistle-like control sounds (n=11)<br><br>Each female was placed in the middle compartment of the preference box. 3 minutes later, sound was broadcast for 60s.			
**Author**	**Strain, Age, Number**	**Detection Method**	**Testing Condition**	**Housing conditions**	**Variables measured**	**Major findings**
(Hanson and Hurley, 2012)	CBA/J<br>2-7 months<br>9 vasectomised males<br>14 females	The video camera and microphone were positioned above the recording cage. Mouse vocalizations were recorded with a condenser microphone (CM16/CMPA, Avisoft Bioacoustics) and sound card (250 kHz sample rate, UltraSoundGate 116 Hb, Avisoft Bioacustics). Video was recorded with a CCD video camera (30 fps),	Prior to experimentation, all animals participated in 5-10 female interactions. All pairs used in the experiments had interacted before.<br><br>Observations took place in the male's home cage inside a recording chamber. A period of 30 minutes for acclimation was allowed.<br>Each male was monitored (audio and video recordings) for 5 minutes prior to the female being added to the cage. Male-female interactions took place over 5 minutes, after which the female was removed.	All mice were housed individually on a 14:10 hour light:dark cycle. Food was provided ad libitum.	<u>Behaviour</u><br>- Female and male behaviour during the interaction<br>- Only male behaviour after the interaction<br>- Nonsocial male behaviours: locomotion, rearing, digging<br>- Other male behaviours: investigation of the female anogenital region and mounting.<br>- Female behaviours:	<u>Characterization of Adult Male CBA/j Mouse USVs and Individual variation in Behaviour</u><br>The most common syllable produced was "up" (23,81% of all syllables produced).<br>USVs ranged 26.300 to 124.000 kHz, with a mean dominant frequency of 74.66260.055 kHz.<br>Individual males varied significantly in the vocalizations they produced.<br>The total number of vocalizations ranged widely among interactions but was not significantly different across males.<br>Duration, dominant frequency and bandwidth parameters of total USVs varied across males. And, also, parameters of different syllable types.<br><br>Males significantly differed in the amount of anogenital investigation they performed and received.<br><br><u>USVs are correlated with other courtship behaviours</u><br>Calls increased in the 10 seconds prior to mounting. Across trials, the total number of syllables in the 10s before mounting was significantly higher than the number of syllables in the 10 sec after mounting.<br>However, there was no difference between the number of syllables in the 10 seconds before mounting and the 20–10 seconds before mounting or between the 10 seconds after

		Q-see 4 channel DVR PCI video capture card, and SuperDVR software (Q-See, Digital Peripheral Solutions Inc.).	Males and females were recorded up to 3 times in experimental days but never the same pair on the same day.<br><br>Females were assessed for estrous phase daily throughout the experiment.		investigation of male anogenital region and rejection of the male (kicking or darting away).<br>- Amount of time that the male and female spent face to face.<br><br><u>USVs</u><br>- Sorted into 9 types based on length, bandwidth and overall shape<br>- Short syllables: less than 10ms in duration<br>- Flat syllables: less than 5 kHz of modulation<br>- Harmonic syllables: contained at least one segment with at least one harmonic<br>- Jump syllables: contained at	mounting and the 10 to 20 seconds after mounting, showing a consistent decrease in vocalizations after mounting rather than an increase before.<br>During the 10 seconds before mounting, ‘‘harmonic’’ syllables made up an average per mount of 31% of the USVs, while ‘‘harmonic’’ syllables only made up an average of 19% per mount of the syllables from the remaining time from the same trials.<br><br><u>Female Oestrous State Influences USVs</u><br>Males mounted females indifferently of their oestrous state.<br>- Number of USVs<br>USVs did not differ in the number of syllables produced nor percent use of different syllables types in relation to the oestrous state of the females.<br><br>- Syllable parameters<br>Syllable parameters differed according to the female oestrus state.<br>Proestrus - syllables lower in dominant frequency and highest in duration and bandwidth<br>Dioestrus - Syllables averaged highest in dominant frequency and lowest in duration.<br>Oestrus - intermediate parameters.<br><br>- Parameters within syllable types<br>Dioestrus females received syllables with a higher average frequency than proestrus females.<br><br><u>Female presence influences USVs</u><br>After removal of the female from the male’s cage, the average number of syllables increased for the following 5 minutes.<br>The differences in average duration, dominant frequency, and bandwidth before versus after female removal did not correspond to female identity, but did correspond to male identity.

					least one break in frequency with no break in intensity (and no harmonics)<br>- Up syllables: increased in frequency (sweep > 5 kHz)<br>- Down syllables: decreased in frequency (sweep > 5 kHz)<br>- Arc syllables: increased and then decreased in frequency, with the highest frequency reaching > 5 kHz above the beginning and end frequencies<br>- U syllables: decreased and then increased in frequency, with the lowest frequency	

					reaching > 5kHz below the beginning and end frequencies<br>Complex syllables: contained two or more directional changes in frequency and > 5 kHz modulation of frequency.	
**Author**	**Strain, Age, Number**	**Detection Method**	**Testing Condition**	**Housing conditions**	**Variables measured**	**Major findings**
(Hoffmann et al., 2009)	F1 generation of wild house mice (*Mus musculus musculus)*<br>20 males<br>12 females<br><br>319 ± 157 days of age	A condenser microphone (UltraSoundGate CM16/CMPA, 15–180 kHz, flat frequency response (± 6 dB) between 25 and 140 kHz) was fixed 20 cm above a hole [Ø=20 cm] in the middle of the lid of the box.	Each male subject was presented with 5 different stimuli (Fresh urine from familiar female, frozen urine from familiar female, fresh urine from familiar female, frozen urine from familiar female and distilled water) in separate trials, with the order of stimulus presentation balanced across subjects, and we used a within-subjects experimental design. Trials were separated by 48 h, so that each subject was tested once every other day with one of the 5 stimulus types.	After weaning, males were housed individually whereas females were kept as sister pairs in type II cages (size: 26.5 × 20.5 × 18 cm, plus high stainless steel covers, mesh width 1 cm) with bedding and nesting material (Abedd). Home cages were kept in an air-conditioned animal room	Total number of USV syllables	- Freezing female urine reduced the males' USV responses to urine samples: males showed a reduction in USVs when presented with frozen versus fresh urine samples and males emitted significantly fewer syllables to frozen than to fresh samples of female urine.<br>- USV responses of males to frozen female urine did not differ from their responses to a neutral stimulus.<br>- Both, familiar and unfamiliar frozen urine did not evoke different responses compared to water.<br>- Males were able to distinguish between urine from unfamiliar versus familiar females, and they perform more USVs for unfamiliar females. But this was only observed when presented with fresh urine.

				with a mean temperature of 20 ± 1 °C and a 12:12 h light:dark cycle (lights on at 04:30 a.m.). Food (Altromin, Germany) and water was provided ad libitum		
**Author**	**Strain, Age, Number**	**Detection Method**	**Testing Condition**	**Housing conditions**	**Variables measured**	**Major findings**
(Kanno and Kikusui, 2018)	C57BL/6J n=63 males<br><br>3 age groups:<br>- Young (8-13 wks; n=27<br>- Middle-aged (20-30 wks; n=17)<br>- Old group (> 30 wks; n=19)	The microphone was set 16 cm above the floor with a sampling rate of 400 kHz (to measure 10-–200 kHz).	Mice were divided into three groups: a young group, a middle-aged group, and an old group. Initially, USVs of the mice in these three groups were recorded during the pretest period (Pre), after which the mice in the young group were randomly divided into three subgroups: Post1, Post2 and Post3.<br><br>Male mice are co-housed for 2 weeks with either intact female, ovariectomized female. Or single housed for two weeks (Post1). Subsequently, male mice are single housed until the age of 30 weeks (Post2) and, finally,	After weaning, mice were housed with same-sex littermates (2–4 mice per cage) in a standard cage (182 × 260 × 128 mm, CREA Japan) until experiments began. During co-housing and recordings (see below), mice were housed in a small test cage (136 ×208 × 115 mm, CREA Japan). Food	Number of calls/min<br>Number of delivered pups	- The number of USVs in the old group was significantly lower than those in the young and middle-aged groups.<br>- There was a significant negative correlation between age and number of USVs.<br>- A significant effect of co-housing with female on USVs was detected in the old group, as the number of calls increased significantly, even though only two and nine of the 19 mice exhibited USVs in the Pre and Post1 tests, respectively.<br>- Co-housing with normal females was conducted again for the young groups after they had become older. No significant effect was observed in the Female group. However, there was a significant increase in the number of USVs in the OVX group and the Single group<br>- Females whose male partners did not vocalize delivered fewer pups when compared to the females whose male partners did vocalize.

			housed again with female for two weeks followed by one week single-housed.	and water were supplied ad libitum, and the animals were kept under a standard 12-h:12-h light-dark cycle. The environment was maintained at a constant temperature (24 ± 1°C) and humidity (50 ± 5%).		
**Author**	**Strain, Age, Number**	**Detection Method**	**Testing Condition**	**Housing conditions**	**Variables measured**	**Major findings**
(Kikusui et al., 2011)	BALB/cAJcl (BALB) and C57BL/6JJc (B6)<br><br>Adult mice:<br>- 6 adult C57BL/6JJc<br>- 7 BALB/cAJcl<br><br>Animals/Litters:<br>- B6-son (5/4)<br>- B6-foster(5/3)	Ultrasonic sounds were detected using a condenser microphone (UltraSoundGate CM16/CMPA, Avisoft Bioacoustics, Berlin, Germany) designed for recordings between 10 and 200 kHz. The microphone was connected to an A/D converter	Breeding pairs were formed with female and male mice from the same strain. When newly born pups were found at the same time in both strains of parents, a part of the litter was reciprocally cross-fostered to parents of the other strain of mice (B6-foster and BALB-foster). The control mice were handled in the same manner as fostered pups but returned to their own parents (B6-son and BALB-son). All litters	Female and male mice were pair-housed in a cage (17.5 cm 6 24.5 cm 6 12.5 cm) for breeding. Food and water were given ad libitum, and all the animals were kept at a constant temperature (23±1 °C) and humidity (40%±10%)	Number of syllables<br>Duration of syllables<br>Frequency	<u>Strain differences in ultrasonic songs</u><br>- B6 males showed a peak at 70–80 kHz, and BALB males at 50–60 kHz.<br>- Average peak frequency of syllables was lower in BALB males and the average interval between syllables was longer in BALB males.<br><br><u>Comparison between the fostered groups and naturally- reared sons</u><br>- B6-sons and B6-foster males showed a peak at 70–80 kHz, whereas BALB mice showed a peak at 50–60 kHz.<br>- Fostering didn't affect song parameters.

	- BALB-son (5/4)<br>- BALB-foster (5/5)	(Ultra-SoundGate 116, Avisoft Bioacoustics, Berlin, Germany) with a sampling rate of 300 kHz and acoustic signals were transmitted to a sound analysis system (SASLab Pro, Avisoft Bioacoustics, Berlin, Germany)	were left undisturbed until weaning (postnatal day (PD) 21). After PD21, they were housed with males of the non-cross fostered controls of the different strain until ultrasound recording at 10–20 weeks of age.	under a 12-h light:dark cycle (light on at 0600).		
**Author**	**Strain, Age, Number**	**Detection Method**	**Testing Condition**	**Housing conditions**	**Variables measured**	**Major findings**
(Lupanova and Egorova, 2015)	House mice (*Mus musculus)*<br><br>6 adult females + 5 adult males	**VOCALIZATIONS**<br>Vocalizations were recorded using a 6.5 mm condenser microphone 4135, preamplifier 2633, measuring amplifier 2606 (all three: Bruel & Kjaer) and sound card (Roland UA-55 Quad-Capture). Recording microphone was fixed on the	A pair of mice was placed into a glass box divided into two parts by a partition that was removed at the beginning of recordings (audio and video). The recordings took place in a soundproof and shaded experimental chamber.<br>For recording of male vocalizations elicited by female traces, males were placed into a cage with soiled bedding from females.	Males were kept individually in cages measuring 15×30×20 cm with plastic faeces tray and metal floor grids. Cages were placed in a separate laboratory room with a temperature of 18–20°C.	Vocalizations characteristics:<br>- Fundamental frequency at the beginning and end of the signal<br>- Maximum and minimum frequency values<br>- Signal duration<br>- Depth and direction of frequency modulation<br>- Presence of noise components	**Behaviour**<br>- Pure components of sexual behaviour (mounting attempts, mountings) took 2.5% of the total contact time and animals displayed aggressive behaviour (chasing, repulsion of the partner, jumps, defensive side and upright standing postures, submission postures) on 28% of the contact time.<br>- Aggressive behaviour was more frequent in females than males.<br><br>**Vocalizations and behaviour**<br>- Audible calls were always accompanied by defensive behaviour components in females.<br>- The defensive call consisted of several fundamental harmonics exceeding the noise level no less than by 8 dB and typically by 15–20 dB. The number of fundamental harmonics varied from three up to eleven, although the signals with three–five harmonics prevailed.

		lid of the box, 25 cm above the bottom. Spectral analysis of audible vocalizations was based on the fast Fourier transformation. The data were estimated by a Hamming window with 2048 points per sample (“Waterfall” and “Cool Edit Pro 2.1” softwares). The tape-recorded calls were digitalized by a 16- bit interface CED1401-plus (Cambridge, “Waterfall” software) at the 125 kHz conversion rate. Noises in the frequency range up to 1 kHz were filtered out. Spectra-temporal analysis of the ultrasound vocalizations			- Subharmonics<br>- Frequency jumps<br>- Spectrum ruptures<br><br>Behaviour:<br>- Position of animals inside the cage<br>- Naso-nasal contact<br>- Naso-anal contact<br>- Sniffing various parts of the body<br>- Chasing<br>- Escaping<br>- Defensive side<br>- Upright postures<br>- Repulsion<br>- Grooming<br>- Mounting<br>- Submission postures<br>Territory exploration	**<u>Male ultrasonic vocalizations</u>**<br>- Spectro-temporal analysis of male ultrasonic vocalizations indicated that the fundamental frequency of calls recorded directly upon male–female encounter imitation varied in a wide frequency range from 38.8 to 88 kHz.<br>- The presence of females stimulated males to emit ultrasonic signals with a lower fundamental frequency, i.e. 38.8–55 kHz.<br>- Male ultrasonic vocalizations were shorter than female defensive calls. Their duration varied from 6 to 218 ms.

		was conducted using the professional Avisoft SASLab Pro 5.2.07 software (Germany) in a semi-automatic mode. Vocalizations were digitalized by an AC-converter Roland UA-55 Quad- Capture at the 192 kHz conversion rate. Noises in the frequency range up to 1 kHz were also filtered out. Spectral analysis of the ultrasound vocalizations was based on the fast Fourier transformation. The data were estimated by a Hamming window with 512 points per sample. Fundamental frequency was measured at 16 points taken at				

		regular time intervals.<br><br>**BEHAVIOUR**<br>Acoustic behaviour of mice was recorded using a webcam Logitech Pro 9000 (resolution 1600×1200, 30 frames per second) and PC stored. Acoustic behaviour was analyzed by the method of temporal slices at intervals of 1 s [19] using the frame-by-frame analysis of video recordings in Virtual Dub 1.10.4 computer program.				
**Author**	**Strain, Age, Number**	**Detection Method**	**Testing Condition**	**Housing conditions**	**Variables measured**	**Major findings**
(Maggio and Whitney, 1986)	340 adult male and 628 adult female mice<br><br>Age range: 75 – 136 days old	Ultrasonic vocalizations were monitored with a QMC bat detector (Model S100, QMC Instruments, London) maintained in	Mice were derived from 15 genotypically based groups of mating pairs such that they resulted in four sets of test animals. Each set was composed of four groups: (1) two inbred progenitor strains and	All animals were born, raised, and maintained in a temperature- and humidity-controlled environment,	- Latency to initial ultrasonic vocalization<br>- Number of 70kHz USVs in	- More ultrasonic vocalization occurred among male-female dyads than among female-female dyads and with shorter latencies.<br>- Dyads containing F_1 hybrid mice were also found to emit significantly more ultrasound than dyads comprised solely of inbreds.<br>- Mounting behaviour was displayed among a significantly greater proportion of male-female dyads than female-female dyads and among a significantly greather

	Genotypes:<br>• BALB/cByJ<br>• BALB/cByJ X C57BL/10Srd<br>• C57BL/10Srd x BALB/cByJ<br>• C57BL/10Srd<br>• DBA/2J<br>• DBA/2J x C57BL/6J<br>• C57BL/6J x DBA/2J<br>• C57BL/6J<br>• DBA/1Bg<br>• DBA/1Bg x C57BL/10Bg<br>• C57BL/10Bg x DBA/1Bg<br>• C57BL/10Bg DBA/1Bg<br>• DBA/1Bg x C57BL/10Bg.D1-Y<br>• C57BL/10Bg.D1-Y x DBA/1Bg	the tuned mode, broadband trim fully off, and set at 70 kHz. Volume was kept fully on during all monitoring. Ultrasonic signals were received via a microphone positioned 13 cm above the wire top of a 29 x 18 x 13-cm transparent plastic test chamber.	(2) two F1 hybrid groups (derived for each set from the reciprocal inbred crosses).<br><br>**Experimental Procedure:**<br><br>One subject was placed in a clean test chamber, transported to the testing room, and placed under the microphone. The introduction of a second mouse (always a female) into the centre of the test chamber initiated the start of that dyad's 3-min test. Ultrasonic vocalizations (70 kHz) were then monitored and quantified by counting the number of 5-sec blocks of time in the 3-min period in which at least one ultrasonic vocalization occurred. No ultrasonic vocalizing resulted in a score of zero, while ultrasonic vocalizing during every 5-sec block resulted in a maximum score of 36. For each test, the latency (-+ 2.5 sec) to the initial ultrasonic vocalization was also recorded. If no ultra-	on a 12:12 L:D cycle (lights on at 0800), with food (Purina Rodent Lab Chow No. 5001) and water always provided ad libitum. The mice were weaned from their parents at 23 days of age and group housed with like-sex littermates in transparent 29 x 18 x 13-cm plastic cages (with wood shavings for bedding and a wire-mesh top supporting a water bottle and food). At 50 days of age, the mice were individually housed in cages identical to those described above.	a 5-sec block of time during the 3min period of testing.	proportion of dyads containing hybrids than among those comprised solely of inbreds.<br>- Mounting was significantly more prevalent among male-female dyads containing hybrids than among those comprised solely of inbreds.<br>- The ultrasonic vocalizing of both F~ hybrid groups typically exceeded that obtained for either within-dyad type inbred progenitor group; this was true for female-female dyads of all four sets and male-female dyads of three of four sets.<br>- The mean amount of ultrasonic vocalizing by F1 hybrids always significantly exceeded that respective set's midparent value.<br>- For both male-female and female-female dyads, across all four sets of mice, the midprogeny mean ultrasound latency of the F~ hybrids was always significantly shorter than the obtained midparent inbred mean.

	C57BL/10 Bg.D1-Y		sonic vocalizing occurred, a latency of 180 sec (i.e., equivalent to the 3- rain trial length) was assigned. Record was also made of those dyads in which mounting behaviour was displayed. For all four sets of mice, and for all groups within each set, both male-female and female-female dyads were tested. In Sets 1and 2, male- female dyads consisted of a male of the specified genotype paired with a standard female of the C57BL/6J strain. Female-female dyads of Sets 1 and 2, and all dyads of Sets 3 and 4, always consisted of within-group pairs of mice. Except for the 28 standard females paired with males of Sets 1 and 2, all animals were tested once.			
**Author**	**Strain, Age, Number**	**Detection Method**	**Testing Condition**	**Housing conditions**	**Variables measured**	**Major findings**
(Marconi et al., 2020)	F3 generation of wild-caught house mice<br><br>22 male adults aged	Mice were video recorded with an IP camera (D-Link DCS-3710) and audio recorded using an ultrasound	Male vocalizations were recorded first without and then during presentation of a female urine stimulus over 3 recording weeks.	Individuals were kept in mixed-sex family groups (standard Type IIL cages, 36.5 x 20 cm and 14 cm	- USV count (total number of USVs emitted during the recording time)	- During the prestimulation phase, the males emitted very few, if any, USVs (e.g. in week 1, 10/22 of the males did not vocalize at all, and the rest on average emitted 2 ± 4 USVs and 1 ± 2 syllable types/ 5 min during this phase).<br>- After the presentation of female urine, half of the males began vocalizing within 1 min and they significantly increased both the number and types of USVs emitted.

	369 ± 40 days	microphone (USG Electret Ultrasound Microphone, Avisoft Bioacoustics/ Knowles FG, Brandenburg, Germany) positioned 10 cm over the centre of the cage. The audio recording set-up included the microphone, an A/D converter (UltraSoundGate 416Hb, Avisoft Bioacoustics) and a laptop (Lenovo T540p, Windows 7) with RECORDER USGH software (Avisoft-RECORDER Version 4.2). Recording settings included a 300 kHz sampling rate and 16-bit format.		high, stainless steel cover, 1 cm mesh width, Tecniplast, Hohen-peißenberg, Germany) until weaning (21 days). At weaning, mice were housed in mixed-sex groups (maximum of four animals per cage) until 5 weeks of age when females were housed in sister pairs and males were singly housed. Each cage contained wood shavings (ABEDD, Vienna, Austria), nesting material (Nestlet, Ehret, Austria), a nestbox (Tecniplast, Buguggiate,	- Repertoire size (number of different syllable types uttered during each recording, 1-15 USV categories,) and<br>- Repertoire composition (number of USVs emitted for each syllable type).	- 95% of the males started vocalizing within ca. 30 s after first sniffing the urine, whereas in only three of 57 of the recordings over 3 weeks did males vocalize before sniffing urine. Males showed an 89 times increase in the USV count and a seven times increase in the repertoire size during the stimulation period compared to prestimulation.<br>- USV count and repertoire size were highly correlated before and during the stimulation.<br>- The males also increased the spectral complexity of their USVs upon sexual stimulation: before stimulus presentation males mainly emitted simple USVs (short duration and without frequency jumps), whereas during odour stimulation, they emitted USVs with greater spectral complexity (long duration, more than one element, harmonic elements). During stimulation (first 5min), 54% of the USVs emitted were ‘up’ syllables, whereas the other syllables were < 10% of the total USVs.<br>- There was high interindividual variation in male USV emission, but mainly during sexual stimulation. Before sexual stimulation, males produced few if any USVs, as previously mentioned, and USV count and repertoire size showed little individual variation (even though the mean and variation in USVs and syllable types emitted during the prestimulation phase tended to increase over the 3 weeks).<br>- During sexual stimulation, a very high interindividual variation in USV was observed.<br>- There was little consistency in nonvocalizing behaviour over time, whereas among vocalizers, low vocalizers tended to remain low and high vocalizers remained high vocalizers.

				Italy) and a cardboard paper roll for environmental enrichment. Mice were provided with food (rodent diet 1324, Altromin, Lage, Ger-many) and water ad libitum. Colony rooms were kept at standard conditions (room temperature: mean ± SD 1⁄4 22 ± 2 °C, in a 12:12 h light:dark cycle with red light on at 1500).		
**Author**	**Strain, Age, Number**	**Detection Method**	**Testing Condition**	**Housing conditions**	**Variables measured**	**Major findings**
(Melotti et al., 2021	Males:<br>C57BL/6J (n=6)<br>BALB/c (n=18)<br>DBA/2 (n=18)<br>B6D2F1 (n=6)	A high-quality condenser microphone (Avisoft UltraSoundGate CM16/CMPA, Avisoft Bioacoustics) was placed inside the box	Experiment I: C57BL/6J, BALB/c, DBA/2 and B6D2F1 were used. Each mouse experienced a 20min social encounter with an adult female mouse (C57BL/6J). One week later, the same mice were presented with fresh urine from	Individually housed in transparent Makrolon type III cages (37 x 21 cm and 15 cm high). Cages were provided with wood chips	Total number of syllables<br>Percentage of syllable types<br>12 types of syllables	<u>**Exp. I**</u><br>- The four laboratory strains (C57BL/6J, BALB/ c, DBA/2 and B6D2F1) differed not only in the composition but also in the complexity (entropy) of their syllabic sequences.<br><br><u>**Exp. II**</u><br>- Sequences of mice from the same strain (either BALB/c or DBA/2) showed some level of individuality in their song syntax.

	Age: 132-136 days old (EXP.I) 188-231 days old (Exp.II)<br><br>Females: Exp. I - 5-HTT +/+ mice with a genetic C57BL/6J genetic background (n=16 65-221 days old)<br><br>Exp. II - 5-HTT +/+ and 5-HTT +/- mice with a C57BL/ 6J genetic background (N= 21 and 14, 75-350 days old).	over the centre of the home cage, pointing downwards and 16 cm from the cage floor. The microphone was connected to a recording device (Avisoft Ultra-SoundGate 416Hb, Avisoft Bioacoustics) controlled by the software Avisoft-RECORDER (version 4.2.27).	unfamiliar and unrelated females.<br><br>Experiment II: BALB/c and DBA/2 adult male mice which had no previous exposure to female urine were exposed to female urine In three weekly test sessions.	(TierWohl Super, J. RETTENMAIER & SO€HNE GmbH, Rosenberg, Germany) as bedding material, a nestlet (BIOSCAPE GmbH, Castrop-Rauxel, Germany; experiment 1) or a paper towel (experiment 2) as nesting material, and a transparent red plastic mouse house (Mouse House, Tecni-plast Deutschland GmbH, Hohenpeißenberg, Germany) and a wooden stick (approximately 1.5 x 1.5 cm and 10 cm long) as		- The set of syllable types that showed high repeatability appeared to vary depending on the strain. However, a descriptive hierarchical cluster analysis of the song sequences also highlighted a remark- able variability in how similar the sequences were within each individual, and this appeared to be the case for both strains.<br><br>**Effect of Genetic Background**<br>- Strain strongly affected the syllabic composition of courtship songs in both experiments.<br>- The courtship songs of B6D2F1 mice showed an overall higher similarity to the maternal strain (DBA/2) indicating a dominant, rather than intermediate, inheritance pattern.<br>- The expression of syllables with frequency jumps varying in complexity (2, 3 and >3 Steps syllables) suggested the presence of a rather intermediate inheritance, since B6D2F1 mice produced more 3 Steps syllables, while the (parental) DBA/2 and C57BL/6J strains produced more 2 Steps and >3 Steps syllables, respectively.<br>- The complexity of the syllabic sequences, measured as sequence entropy, varied across strains and was affected by sequence length (syllable production rate). C57BL/6J mice produced syllabic sequences at a lower rate but with higher complexity, while BALB/c mice vocalized at a higher rate but with less complex syllabic sequences, and the DBA/2 and B6D2F1 strains showed intermediate patterns.<br><br>**Individuality in Courtship Songs**<br>- The overall complexity of the song sequence, was repeatable across test sessions. Repeatability, however, seemed to differ depending on the strain considered.

				environmental enrichment.		
**Author**	**Strain, Age, Number**	**Detection Method**	**Testing Condition**	**Housing conditions**	**Variables measured**	**Major findings**
(Musolf et al., 2010)	F1 offspring of wild adult house mice<br><br>Exp. I<br>N=15 males (319 ± 156 days old)<br><br>Exp. II<br>32 female mice (306 ± 169 days of age	A condenser microphone (UltraSoundGate CM16/CMPA, 15–180 kHz, flat frequency response (±6 dB) between 25 and 140 kHz) was fixed 20 cm above a hole (diameter 1⁄4 20 cm) in the middle of the lid of the box. For monitoring USVs, we used an UltraSoundGate 116 Avisoft Bioacoustics, Berlin, Germany) and an external soundcard (Edirol UA-101, 24 bit/192 kHz 10-in/10-out Hi-SPEED USB (USB 2.0) audio interface for multitrack computer recording). Settings	**<u>Exp. I – male USV production</u>**<br>Each male was presented with seven different stimuli (Familiar female fresh urine or soiled bedding, Unfamiliar female fresh urine or soiled bedding, unfamiliar male fresh urine and none - distilled water or clean bedding) in separate trials, with the order of stimulus presentation balanced across subjects.<br><br>**<u>Exp. II – Female responses to USVs</u>**<br>Oestrus females were given the choice between USV playback versus background noise (Exp.IIa) or between two simultaneous USV playbacks from a littermate brother (henceforth: familiar kin) versus an unfamiliar nonkin male.	All subjects were raised in mixed-sex family groups until weaning at 21 days of age. At weaning, males were housed individually to prevent fighting, whereas females were kept as sister pairs in type II cages (26.5 x 20.5 cm and 18 cm high, plus high stainless steel covers, mesh width 1 cm) with bedding and nesting material (Abedd: aspen wood chips and shavings). Home cages were kept in standard conditions (mean	Exp. I<br>- Number of USV syllables per 30min recording<br>- Number of complex syllables<br><br>Exp. II<br>- Initial preference, latency to enter Y-maze arm<br>- Number of visits<br>- Times spent in designated areas	**<u>Exp. I – male USV production</u>**<br>- Bedding from females, both familiar and unfamiliar, elicited more USVs from males than the clean bedding.<br>- Males produced only a few USVs to water and male urine in comparison to the number of USVs uttered during exposure to female urinary cues.<br>- Male urine elicited no more USVs than distilled water, but males vocalized significantly more to both fresh female urine stimuli than to the corresponding water control.<br>- The proportion of complex syllables uttered did not differ between stimuli except for a significantly higher ratio in response to unfamiliar female than male urine.<br>- Urine from immature unfamiliar females elicited no more USVs than male urine or water and differed significantly from adult unfamiliar female urine.<br>- Adult female urine elicited more USVs from male mice than male urine.<br>- Urine from unfamiliar females elicited more USVs from males than that from familiar females<br>- With soiled bedding as a stimulus, however, no significant difference was found in the USV responses of males to novel versus familiar females.<br>- No significant effect of social experience on the production rate of syllables.<br><br>**<u>Exp. II – Female responses to USVs</u>**<br>- Females spent more time at the fence in front of the USV playback speaker, and more time in the zones on the sides with USV playback, than on the control side playing background noise.<br>- In experiment 2b social experience had no impact on females' behaviour.

Author	Strain, Age, Number	Detection Method	Testing Condition	Housing conditions	Variables measured	Major findings
		included sampling rate at 250 kHz and a format of 16 bit.		temperature 20±1 °C and 12:12 h light:dark cycle; lights on at 0430 hours). Food (Altromin, Lage, Germany) and water were provided ad libitum.		- Females spent significantly more time on the side playing USVs from unfamiliar non-kin compared to familiar kin in the first trial.
**Author**	**Strain, Age, Number**	**Detection Method**	**Testing Condition**	**Housing conditions**	**Variables measured**	**Major findings**
(Nicolakis et al., 2020)	Wild-derived (F3) house mice (Mus musculus musculus)<br><br>N= 52 (26 males + 26 females)<br><br>Age: 249 ± 36 d old (mean ± SD	An ultrasound microphone (USG Electret Ultrasound Microphone, Avisoft Bioacoustics / Knowles FG) was placed in a fixed position 10 cm above the center of the male compartment or the middle of the cage. The microphone was connected to an A/D-converter (UltraSoundGate 416Hb, Avisoft Bioacoustics). Recordings	USV recordings were conducted during the mice active period under red light, i.e. after the onset of the dark phase (15:00–18:00 h) in a separate, closed room. Mice were recorded in a plexiglass cage (modified from a Type III cage, Tecniplast, Germany; floor measurements: 36.5 × 21 × 15 cm, top measurements: 42.5 × 27 × 15 cm) equally divided into two compartments by a perforated plexiglass divider.<br>Before each recording, a male mouse was gently transferred into one of the two compartments, which was covered with	Mice were weaned at 21d and kept in mixed-sex groups with ≤4 siblings per cage until the age of 5 weeks (35d). After this time, adult males were housed individually to pre- vent fighting and females were housed in sister-pairs whenever possible. Mice were housed in standard Type IIL cages	USVs<br>- Total number of USVs<br>- Length of USVs<br>- Frequency of USVs<br>- Number of syllables (short, simple and complex)<br>- Mean frequency<br><br>Reproductive parameters	**<u>Phases of courtship</u>**<br>- Male mice emitted 5x more USVs and produced more types of syllables during than before direct interactions.<br>- In both phases there was a positive correlation between vocal performance and vocal repertoire, so that the mice that emitted more USVs also emitted more syllable types.<br>- The vocal repertoire first increased with the number of vocalizations but then plateaued after circa 10 syllable types.<br>- During direct interactions the mice also emitted longer syllables compared to the introduction phase.<br><br>**<u>Female sexual receptivity</u>**<br>- During the introduction phase, vocal performance did not differ when males were exposed to females of any of the four estrous states.<br>- Female receptivity had no significant effect on the mean length of USVs during either phase.<br>- Mice produced a larger vocal repertoire when presented with an unreceptive female (vs. a receptive female) during the introduction phase but not during the direct interactions.

		were conducted on a computer (Lenovo T540p, Windows 7) using the RE-CORDER USGH software (Avisoft-RECORDER Version 4.2) with a sampling rate of 300 kHz and 16 bit format.	a standard cage cover (1 cm mesh width). A female was then transferred into the other compartment of the cage, which allowed both olfactory and visual cues through the perforated divider, but restricted physical contact. Recordings were conducted in two consecutive phases, lasting 10min each.<br>- Phase 1 (introduction phase): only male vocalizations were recorded, while exposed to the female on the other side of the divider, i.e. with visual, and chemical communication.<br>- Phase 2 (interaction phase): The divider was removed to allow direct, physical interaction.	(36.5 × 20 × 14 cm cages, Tecniplast, Germany), with food (rodent diet 1324, Altromin, Germany) and water provided ad libitum. Cages were covered with stainless-steel covers (1cm mesh width) and provided with bedding (ABEDD, Austria) and nesting material (Nestlet, Ehret, Austria). A nest box (Tecniplast, Germany) and a cardboard paper roll were provided for environmental enrichment. Home cages were kept at standard conditions	- Latency to first litter<br>- Number of litters<br>- Number of offspring<br>- Number of offspring in first litter<br>- Number of offspring /litter	- Female receptivity also influenced the grand mean frequency of USVs emitted during introduction but not during direct interaction.<br>- USVs emitted in the presence of receptive females had a lower grand mean frequency compared to unreceptive females.<br><br>**<u>Genetic relatedness</u>**<br>- During the introduction phase, males tended to have a higher vocal performance when presented with an unrelated female compared to a related female though not during direct interactions.<br>- Males emitted more syllables when presented with unrelated compared to related females in the introduction phase.<br>- The vocal repertoire did not differ between unrelated and related pairs in any phase, however, unrelated mice always emitted longer USVs than related mice in both phases.<br>- The parameters with the greatest discriminatory ability between related and unrelated pairs were number of short syllables, grand mean frequency and mean USV length in the introduction phase and number of simple syllables, mean USV length and number of short syllables in the interaction phase.<br>- in the introduction phase males emitted a larger number of simple syllables with a longer duration and higher frequency to unrelated females, whereas they emitted a larger number of short syllables at lower frequencies to related females.<br>- During direct interactions, unrelated mice emitted USVs with a longer duration and used a larger number of complex syllables, while related mice emitted a larger number of short and simple syllables.<br>- The number of syllables used per syllable type tended to differ between related and unrelated mice during the introduction phase but not during direct interactions.<br>- The types of syllables differed between the males that were presented with a related vs unrelated female.

				(mean ± SD room temperature: 22 ± 2 °C) under a 12:12 h light-red light cycle (red lights on at 15:00).		**Reproductive success**<br>- Unrelated pairs sired significantly more offspring than related pairs during the entire breeding period.<br>- Unrelated pairs gave birth to more litters, while the litter size did not significantly differ between unrelated and related pairs.<br>- Unrelated pairs tended to have a shorter latency to the first litter, however, the effect of relatedness depended on female receptivity.<br>- Among pairs with females that were initially receptive, un- related pairs had a significantly shorter latency to first litter than related pairs. When females were initially unreceptive, there was no difference in latency to first litter between related and unrelated pairs.<br><br>**USV emission and reproductive success**<br>- The results for unrelated and related pairs depended upon the experimental phase.<br>- Related mice emitting USVs at a higher grand mean frequency and with a larger vocal repertoire in the introduction phase had a shorter latency to the first litter.<br>- Unrelated pairs' USV emission during the introduction phase did not correlate with latency to first litter.<br>- The mean length of USVs negatively correlated with LFL but only in unrelated pairs during the interaction phase.<br>- unrelated pairs that had a higher vocal performance, tended to have a shorter latency to first litter.<br>- There was a significant negative correlation between the number of simple syllables and latency to first litter.<br>- Unrelated mice emitting longer USVs and with a higher number of simple syllables during direct interactions had a shorter latency to the first litter.<br>- USV emission and reproductive success were not affected by male age or age differences. However, there was a negative correlation between female age and the reproductive success in unrelated but not in related pairs.

						- The age of females in unrelated pairs was correlated with the vocal performance, vocal repertoire and grand mean frequency of USVs emitted during direct interactions.<br>- Syllable type usage in both phases did not differ between pairs with a short or long latency to the first litter
**Author**	**Strain, Age, Number**	**Detection Method**	**Testing Condition**	**Housing conditions**	**Variables measured**	**Major findings**
(Nyby et al., 1977)	Exp.I<br>Male DBA/2J (n=28)<br>Female C57BL/6J (n=4)<br><br>Exp. II<br>Subjects DBA/2J males (n=20; 80 ± 3 days of age)<br><br>Stimulus Female C57BL/6J mice (n=13;110-240 days of age)<br>Male C57BL/6J (n=20; 110-240 days of age)<br><br>F_1 C57BL/6J x DBA/2J hybrid females (n=7; 135-	Ultrasounds were monitored using a Holgate ultrasonic Mk IV receiver. At the settings used the receiver transforms ultrasounds between approximately 60 and 80 kHz and louder than 60 dB (re 0.0002 dynes per CM2) into audible sounds	Exp. I<br>Two similar replications were performed.<br>1) During the first phase each subject was presented with an anaesthetized female enclosed in a plastic bag for one 3-min trial. After placing the female in the bag, the opening was twisted shut and secured with a paper clip. A clean bag and paper clip were used for each trial in this and subsequent phases.<br>2) The second phase consisted of five 3-min trials during which half the males were	No information provided	Exp. I<br>- Number of USVs emitted in each 30s block<br>- Latency to first ultrasound emission	<u>**Exp. I**</u><br>- During the 1st phase no ultrasounds were emitted by any of the males to the completely enclosed female.<br>- During the 2nd phase, no statistically significant differences were found between the rear and front exposed conditions for number of 30s blocks containing ultrasound or for latency to first ultrasound.<br>- During the 3rd phase, responses to the two stimulus conditions did not differ statistically for 30s blocks of for latency to first ultrasonic emission.<br>- In the 4th phase, a reduction in ultrasound occurred when the female was again completely enclosed in a plastic bag.<br><br><u>**Exp. II**</u><br>- Higher level of ultrasonic emissions to the female swabs and lower level of ultrasonic emission to the other two conditions (male and control facial swabs).<br>- The male swabs and control swabs were not statistically different in eliciting ultrasounds.<br>- Latency to first ultrasonic emission was lower in the female swab group and lower in the remaining conditions.<br><br><u>**Exp. III**</u><br>- Vaginal swabs elicited significantly more 5-s blocks containing ultrasound than control swabs.<br>- Latency to first ultrasound in seconds to the vaginal swabs did not differ significantly from that for control swabs.<br><br><u>**Exp. IV**</u>

	237 days of age).<br><br>Exp. III Subject - DBA/2J males (n=14; 95 days of age)<br><br>Stimulus – Female C57BL/6J mice (n=13;110-240 days of age)<br><br>F_1 C57BL/6J x DBA/2J hybrid females (n=7; 135-237 days of age).<br><br>Exp. IV DBA/2J males (n=17; 98 ± 3 days of age)<br><br>Urine donours – 17 females and 17 males which the subjects had been		tested with an anaesthetized female whose rostral half anterior to the hind legs had been covered with a plastic bag. The remaining males were tested with a female whose caudal half posterior to the front legs was covered with a plastic bag.<br>3) During the third phase the stimulus conditions were reversed so that males previously tested with an exposed front were now exposed to the rear and vice versa.<br>4) In the first replication only, a fourth phase occurred in which all animals were again tested for one 3-min trial			- Female urine elicited much more ultrasounds than male urine.<br>- Number of USVs emitted in response to male urine did not differ from the control swab condition.<br>- Latency to ultrasound emission was shorter for female urine.<br><br>**<u>Exp. V</u>**<br>- Social experience in adulthood had no effect upon the hybrid males.<br>- Experienced and inexperienced animals did not differ in number of 5-s blocks containing ultrasound or latency to first ultrasound.<br>- Experience did not interact with urine condition for either 5-s blocks containing ultrasound or latency to first ultrasound.<br>- Female urine elicited more ultrasounds than male urine and there was a shorter latency to first ultrasound emission in the female urine group.

	previously paired in Exp. II.<br><br>Exp. V<br>C57BL/6J x AKR/J males (n=28; 114-213 days of age)<br><br>Urine donours<br>C57BL/6J x DBA/2J females (n=4; 75 days of age)		with a completely enclosed stimulus female.<br><br>Exp. II<br>Male subjects were given daily 3-min exposures in their home cages to male and female stimulus mice for eight consecutive days. The order of presentation of males and females was counterbalanced within days and reversed each day.<br>Three different classes of olfactory stimuli were used in this experiment: male facial swabs, female facial swabs and control swabs.<br>Over a period of 7 days, each subject was tested for ultrasonic emissions in response to each of the three stimulus conditions<br><br>Exp. III<br>Two different stimuli were used: female vaginal swabs and control swabs. No monitoring of the females oestrous cycle.			

			Exp. IV<br>Three different stimuli were used: ale urine on a cotton swab, female urine on a cotton swab, and a control swab immersed in distilled water to control for moisture.<br><br>Exp. V<br>he subject males were randomly divided into two groups of 14 animals and only one group was given social experience. The 8-day social experience regime was identical to that described in Exp. II.. One week after completing the social experience regime, all subjects were given one additional 3-min exposure to a female in the test room. Two olfactory stimuli were used: male urine and female urine.			
**Author**	**Strain, Age, Number**	**Detection Method**	**Testing Condition**	**Housing conditions**	**Variables measured**	**Major findings**
(Pomerantz et al., 1983)	Male and female Swiss Webster<br>3 – 4 months old	Ultrasonic vocalizations were monitored using a Holgate (Model Mark V) ultrasonic	<u>Surgery</u><br>Stimulus males received either unilateral inferior laryngeal nerve transections (n=10) or	The females were housed in groups of 3 or 4 in plastic cages (24×18×13	Behavioural measures:<br>- percentage of test time spent visiting, average visit	<u>**Exp. Ia**</u><br>- Females m both vaginal conditions spent a significantly greater percentage of time during the test session visiting the vocalizing male than visiting the devocalized male.

	Exp. Ia<br>9 females (experimental animals)<br>Males used as stimuli (no information provided on the number of animals used)<br><br>Exp. Ib<br>16 adult females<br>18 males (10 devocalized + 8 vocalizing males)<br><br>Exp. II<br>Female house mice (n=28)<br>8 devocalized males	detector and a QMC Mini-Bat Detector tuned to a centre frequency of 70 kHz. The receivers for the ultrasound detectors were mounted 15 cm from the floor directly above each tethered male. The Holgate ultrasonic detector was used for all the 3 min pre-tests, both detectors were used during the preference tests. In Exp.II a QMC (Model GTI) ultrasound generator was used.	were sham operated (n=6).<br>Females were ovariectomized.<br><br><u>Apparatus</u><br>The testing chamber consisted of a plastic cage (24×18×13 cm) with an opaque partition (13 cm height) mounted at the centre of one end of the cage and running one-half the length (12 cm) of the cage. A neutral zone was defined from the front of the cage to the beginning of the partition, and on either side of the partition, two equal size compartments were defined. In each compartment, a stimulus male was tethered by anchoring one end of a 12 cm cord at the back of the compartment (10 cm above the floor) and attaching the other end of the cord to the safety pin located in the male's neck. When tethered, the two stimulus males were unable to see or interact with one an-other.<br><br><u>Preference Test</u>	cm), the males were individually housed. All animals were provided with food and water ad lib and maintained on a 12:12 light-dark cycle.	duration, and number of visits.	- The percentage of time spent with the vocalizing male did not differ significantly between estrous and diestrus females. Although, the average visit duration by both oestrous and diestrous females was significantly longer with the vocalizing male than with the devocalized male.<br>- Females m vaginal estrus and in vaginal diestrus dad not differ significantly in the number of vocalizations they elicited from intact stimulus males.<br>- Estrous females were present in the compartment with the vocalizing males during 65±6% of the total number of 4-sec blocks with male ultrasounds and diestrous females were present during 71±6% of the total number of 4-sec blocks with male ultrasounds. No copulatory behaviors were exhibited by the males during any of the test sessions.<br><br>**<u>Exp. Ib</u>**<br>- Ovariectomized (OVX) females receiving EB+P spent a significantly greater percentage of the total test time visiting the vocalizing male than visiting the devocalized male.<br>- OVX females exhibited a longer average visit duration but not a significantly higher number of visits to the vocalizing male.<br>- Females receiving oil injections did not exhibit a preference for either stimulus male.<br>- OVX females receiving hormones elicited significantly more ultrasonic vocalizations from tethered males than vehicle-treated females. But this difference was absent during periods when females were ºresent in the vocalizing male’s compartment.<br>- All females elicited a high rate of ultrasonic calling while they were in proximity to the vocalizing male. Ul trasonic calls were detected in 81±% of the 4-sec blocks during which hormone-treated females were present in the vocalizing male's compartment and in 95±5% of the 4-sec blocks during which oil-treated females were present in the vocalizing male's compartment.

			Preference tests were conducted under dim red illumination, beginning two hours after lights off. Tests were 3 min m duration and were begun by introducing the experimental female into the middle of the neutral zone of the test chamber. Female behaviour was recorded without the experimenter's being aware of the hormonal condition of the animal. During preference tests, visits by the female to a tethered male and the time spent visiting a tethered male were recorded. Visits were defined by the female leaving the neutral zone and entering one of the compartments containing a tethered male. Also, ultrasonic vocalizations made by the tethered males were monitored by two experimenters. All behaviours were recorded on an Esterhne-Angus event recorder. The location of each type of stimulus and the type of detector			**<u>Exp. II</u>**<br>- A preference for the devocalized male that had synthetic ultrasounds being produced behind him was exhibited by both intact females and OVX females receiving EB+P, but not by oil-treated OVX females.<br>- This preference for the male paired with synthetic ultrasounds was observed for all behavioral measures (i.e, percentage of test time spent visiting, average visit duration, and number of visits) except for average visit duration of OVX females receiving EB+P which showed only a non-significant trend to be longer with the male paired with synthetic ultrasounds.<br>- Intact females and OVX females given EB+P did not differ significantly on any measure of female behaviour, but both of these groups of females spent significantly more time visiting the devocalized male paired with syn- thetic ultrasounds, and exhibited a longer average visit duration with him than oil treated OVX females.<br>- The number of visits made to the male paired with synthetic ultrasounds was similar across the 3 groups of females.

			used to monitor ultrasounds were varied randomly across preference tests.<br><br>Exp. Ia<br>During each preference test, a vocalizing stimulus male was tethered in one compartment of the test chamber and a devocalized stimulus male was tethered in the other compartment Females were given 2 preference tests in random order, one during oestrus and the other during dioestrus. Oestrus and dioestrus were determined by vaginal smears.<br><br>Exp. Ib<br>Females were ovariectomized and treated with hormones to induce oestrus. Females received either l0/ug of estradiol benzoate for 3 consecutive days followed by I mg of progesterone 6 hr be-fore the test (EB+P, n=8) or oil (0.05 cc sesame oil) injections each day (n=8)			

			Females were tested for preference behaviour using the same method employed in Exp. Ia.<br><br>Exp. II<br>The vaginal cytology of 7 females was moni tored for 10 days, and these females were given a preference test when in vaginal oestrus. Fourteen females were OVX and 10-14 days later received either 10 ug EB for 3 days followed by 1 mg of progesterone 6 hr before the test (EB+P, n=7) or oil injections each day (Oil, n=7). All OVX females were given a preference test 6 hr after the final injections. Both of the tethered males used in the preference test were devocalized Behind one of the males a QMC ultrasound generator, set to produce 70 kHz ultrasounds at a rate of 4/sec was placed near a small hole in the back of the testing chamber. The back of the chamber was opaque as to minimize the effect of possible visual cues produced by placing the ultrasound			

			generator behind each compartment. The ultrasound generator was turned on and off at 2-sec intervals each time an experimental female entered and remained on the side of the test chamber containing the generator			
**Author**	**Strain, Age, Number**	**Detection Method**	**Testing Condition**	**Housing conditions**	**Variables measured**	**Major findings**
(Ronald et al., 2020)	CBA/J<br>9 focal male subjects and 24 non-virgin females + 6 non-virgin males used for stimulus	All vocalizations were recorded via an Avisoft-UltrasoundGate 116H Recorder (#41163; Avisoft Bioacoustics, Berlin, Germany with a sampling rate of 250 kHz) attached to a Dell Optiplex 960 Computer running Avisoft Recorder Software and a 16-bit condenser microphone (CM16/CMPA; Avisoft Bioacoustics, Berlin, Germany; 200 kHz maxi- mum	<u>Female vocalization recordings</u><br>Female squeaks were recorded by placing a male and a female together into a standard mouse cage with clean bedding fitted within the behavioral setup arena for 20 minutes.<br>Female USVs were recorded by placing 60 µL of previously frozen male urine on a cotton ball in the center of a standard cage and allowing a single female to explore the arena for 20 minutes.<br><br><u>Behavioural experiments</u><br>Each male was randomly exposed to each of the five treat- ments twice (e.g., USVs only, squeaks only, urine only,	All focal animals were housed in same-sex social groups of 3 mice in standard plastic cages for laboratory mice (28.5 x 17.5 cm and 12.5 cm tall) with pine bedding and nesting material.<br>Females used for urine collection and vocal recordings were housed in pairs or groups of 3.<br>All animals were provided	<u>Non-vocal behaviours</u><br>- Investigation of the stimulus circle<br>- Investigation of non-stimulus circle<br>- Rearing<br>- Digging<br>- Grooming<br><br>USVs<br>- Total number of USVs<br>- Number of vocalizations with a	<u>Male non-vocal behaviours</u><br>- The non-vocal behavioral responses of males varied with the types of female signals that were presented.<br>- Males investigated more when female urine was present regardless of whether female urine was presented alone or paired with a female vocalization.<br>- Males spent significantly more time investigating when female urine was presented compared to when only USVs were presented or to when only squeaks were presented.<br>- Males did not spend more time investigating the multimodal stimuli (i.e., urine+USVs or urine+squeaks compared to female urine presented in isolation.<br>- Change in male behavior was in direct response to female signal presentation.<br>- Males did not change their investigative behavior with the presentation of just USV.<br>- No other non-vocal behaviors tested, including self-grooming, rearing, or digging showed a significant interaction between stimulus presentation (e.g., before and after stimulus) and stimulus treatment.<br>- No find a significant main effect of stimulus on any of these behaviors.<br>- Males decreased the proportion of time they spent rearing, and digging after the stimulus presentation.<br><br>**<u>Male vocal behaviours</u>**

		range) directly above the arena	urine+squeaks, urine+USVs).<br>A behavioral trial began when a randomly selected male was placed into the experimental arena for 10 mins of habituation time while video and audio recording occurred to serve as a baseline for vocal and non-vocal behaviors	with ad libitum food and water and housed on a 14:10 light:dark cycle.	harmoni c structur e with a fundame ntal frequenc y at 50 kHz and 'Oth- ers'	- Males varied considerably in their vocal production, from a range of 123 total vocalizations across all trials to 7,286 total vocalizations across all trials.<br>- In contrast to non-vocal behaviors, male vocal behaviors were differentially influenced by urine and vocalization presentation.<br>- No significant interaction between stimulus treat- ment and stimulus presentation time but there was a significant main effect of stimulus presentation time.<br>- Males produce a higher rate of total USVs after the presentation of the stimulus (0.21 ± .05) than before (0.13 ± 0.05).<br>- The majority of the USVs were produced within 5 minutes after the beginning of the stimulus presentation.<br>- Neither the presentation of squeaks nor USVs in isolation changed the total rate of USV production.<br>- After the presentation of USVs paired with urine, males responded with a great increase in their total USV rate.<br>- The addition of urine to squeaks did not change the proportion of USVs given before or after the stimulus.<br>- Males gave relatively few 50 kHz harmonic calls: of the 11, 954 total USVs given in the 5 minutes following playback, only 1, 425 (roughly 12% of these vocalizations) contained a 50 kHz harmonic.<br>- Males produced longer USVs in the 5 minutes following the playback (0.014 ± 0.001) compared to USVs given before the playback (0.012 ± 0.001)
**Author**	**Strain, Age, Number**	**Detection Method**	**Testing Condition**	**Housing conditions**	**Variables measured**	**Major findings**
(Sales, 1972)	3 strains:<br>● "impure albino strain (E.N.)"<br>● T. O. Swiss albino strain	BAT detector and a microphone and cathode-ray-oscilloscope were used during all behavioural observations and where	10 heterosexual encounters involving 4 males and 10 females were observed in daylight. Each male met either 2 or 3 females singly in his home age on different days. Each encounter lasted 30 minutes.	Kept individually in cages 14 x 12 x 30 cm, or in groups in cages 24 x 12 x 40 cm.<br><br>Feed: A standard diet	Categories of behaviour<br>- Male approach female<br>- Female approach male<br>- Male nose female	Ultrasound emission was highly correlated with "male sniff female" and "male mount" but less so with "male approach" and "male push past female" and with "male nose female".<br>The pulses emitted during mounting appeared to be related to the pelvic thrusts of the male.<br>"Audible cries" emitted by the female were often heard and recorded at the same time but were not synchronous with the ultrasounds.<br><br>Ultrasounds were recorded in 91/100 encounters.

Author	Strain, Age, Number	Detection Method	Testing Condition	Housing conditions	Variables measured	Major findings
	Grey C3H strain	possible the signals were recorded at high speed on a tape-recorder responding to at least 100 kHz.		of B 41 pellets and water was freely available to all animals and vegetable matter was given two to three times weekly	- Female nose female<br>- Male sniff female<br>- Male mount female<br>- Intromission<br>- Ejaculation<br>- Male groom female<br>- Male poke or bite female<br>- Male push past female<br>- Rejection by female<br>- Maintenance activities<br>- Maintenance activities by both<br><br>Number of behavioural categories performed<br><br>Number of times that ultrasounds were detected simultaneously<br><br>Frequency and duration of ultrasounds	In each strain, mounting was often accompanied by the emission of sequences of long pulses. Each sequence lasted 0,5-7.0 sec and consisted of between 3 and 25 or more pulses, each 50-300 msec in duration, emitted at intervals of up to 200 msec.<br>The calls of E. N. mice were at frequencies of 50-112 kHz, generally 60-85 kHz, whereas those of C3H mice were mainly between 40 and 70 kHz with a range of 30 to 90 kHz.<br>T.O. Swiss mice produced pulses at frequencies within the total range of 30 to 112 kHz.<br><br>These ultrasonic pulses appeared to be produced by the male mice, but ultrasound emission in adult mice is not confined to the males. Ultrasounds at frequencies between 60 and 80 kHz were detected when an albino female from a communal cage was introduced into the cage of an isolated female and also when a non-pregnant female was introduced into the cage of a pregnant female.

(Sugimoto et al., 2011)	- C57BL/6J<br>- BALB/cAnN,<br>- BFM/2,<br>- BLG2,<br>- CAST/Ei,<br>- CHD,<br>- HMI,<br>- JF1-s+,<br>- KJR,<br>- MSM,<br>- NJL,<br>- PGN2,<br>- SWN<br>- MCH<br><br>2 months old	Ultrasonic signals were recorded using an ultrasound micro- phone (CM16/CMPA Condenser ultrasound microphone, Avisoft-Bioacoustics) and recorder (UltraSoundGate 116H, Avisoft-Bio- acoustics). The microphone was positioned approximately 10 cm above the cage that contained the mice. At the same time, the mice were recorded with a digital video camera (Panasonic, Osaka).	Two-month-old, male mice were mated with female mice of their own strain for 1 month. After 1 month, the male mice were housed individually for 2 days. Female mice of MCH that were older than 10 weeks were injected with pregnant mare serum gonadotropin (PMSG) to control the sexual cycle. On the test day (after the males had been housed individually for 2 days), each male mouse was transferred to a small cage (12620 cm) with its wood chip bedding , and then an MCH female mouse was introduced into the small cage. Immediately after the female had been introduced, recording of sound and video was started. Sounds and movies were recorded for a maximum of 15 min. Recording was stopped 3 min after the male started vocalizing, but if vocalization was not present, then recording was terminated after 15 min. During this test, intromission and	All animals were maintained at the NIG under a 12 h light/dark cycle (light from 8:00 to 20:00) in a temperature-controlled room (23±°2C).	- Number of USVs emitted<br>- Call latency<br>- Duration<br>- Start frequency<br>- End frequency<br>- Minimum frequency<br>- Mid frequency<br>- Maximum frequency<br>- Duration until maximum frequency<br>Behaviours recorded:<br>- Grooming<br>- sniffing of genitals<br>- body sniffing in both sexes<br>- attacking (biting and kicking<br>- mounting by males<br>- avoidance behaviour<br>- clicks in females.	**<u>USVs in the male-female interaction test</u>**<br>- No USVs were detected from the females during interaction with devocalized male mice.<br>- When sham-operated male mice were introduced to the female, frequent USV was detected.<br><br>**<u>Characterization of USV patterns in 13 inbred mouse strains</u>**<br>- Most of the wild- derived mice did not emit USVs. In particular, for PGN2, CAST/ Ei, HMI, and NJL mice, USVs were emitted in fewer than 40 % of the pairs of mice we examined.<br>- The laboratory mouse strains (B6 and BALB/c) emitted USVs in all trials. The frequency of emission in the trials showed a strain effect. In addition, call latency, i.e. the time from an encounter with a female to emission of the first call, showed a significant effect of strain on one-way analysis of variance, and was generally longer in wild-derived mouse strains than in laboratory mouse strains.<br>- Among the nine inbred mouse strains, BALB/c mice displayed the lowest frequency and longest duration for the USVs, whereas BLG2 mice showed the shortest duration and highest frequency.<br><br>**<u>Strain differences in the waveform composition of USVs</u>**<br>- Waveforms were characterized into nine types (flat, short, upward, downward, a-type, u-type, jump).<br>- The waveform categories showed a significant effect of strain.<br>- Upward, Downward, Jump, Short, and A-type waveforms all showed a significant effect of strain.<br>- BALB/c mice showed a high percentage of A-type waveforms, whereas B6 and BLG2 mice showed a high percentage of Short-type calls.<br>- The main characteristics of the waveform compositions in CHD, JF1, KJR, and MSM mice appeared to be similar.<br>- The pattern of USVs was as different among closely related strains as among genetically remote strains.<br><br>**<u>Principal component analysis (OCA) of ultrasonic vocalization</u>**

			ejaculation were not observed, because the session was a maximum of 15 min long. If the male mouse did not emit USV, it was returned to its cage, together with a female of same strain. One or a few weeks later (minimum 1 week), this male mouse was tested again. Recording was performed during the late part of the light phase, 14:00–18:00 pm.<br><br><u>Devocalization</u><br>Male B6 mice were devocalized by surgical bilateral section of the inferior laryngeal nerve. Sham surgeries were performed in another group of mice.<br><br><u>Playback experiment</u><br>USVs were recorded from KJR mice.<br>10-week-old MCH females were used for the playback experiment. Two days before the test, female MCH mice were injected with PMSG to control the oestrous cycle. Subsequently, 24 hours before the test, the mice were transferred into			- Highly correlated variables were combined into one variable. More than 90% of the variance in the data was explained by principal components (PC) 1 to 5 (PC1– PC5).<br>- For PC1, the frequency at each point and duration of each waveform showed high factor loadings. Frequency and duration were negatively correlated; thus, a high score for PC1 indicated USV of high frequency with short duration.<br>- For PC2, call duration and the maximum frequency of the Flat waveform were positively correlated, but the minimum frequency was negatively correlated.<br>- For PC3, the percentage composition of Jump and the maximum frequency of U-type waveforms displayed high factor loadings.<br>- PC4 indicated duration until the maximum or minimum peak for waveforms of the Short, A-type, and Jump type.<br>- For PC5, the slope of the Downward waveform showed high factor loadings. In summary, major differences among the mouse strains occurred with respect to frequency and duration, as well as for Flat, U-type, and Downward waveforms.<br>- The BALB/c mice displayed a high score for PC1, which indicated calls of lower frequency and longer duration. The KJR mice displayed high scores for PC2–4 as compared with other strains. CHD and JF1 mice, and BLG2 and SWN mice, displayed similar USV patterns to each other, but the USV patterns of the BFM/2 and MSM mice were unique among the nine strains.<br><br>**<u>Analysis of behaviour during female-male interactions</u>**<br>- No significant effect of strain on any of the behavioral components including the positive behavior of females toward males (genital sniffing and grooming).<br>- Social behavioral components were not significantly different among strains.<br>- Significant strain effect only for clicks made by female mice.<br>- Positive correlation between clicks from females and kicking of males by females.<br>- KJR strain triggered the fewest female clicks.

			the test room. The test box (35x20 cm, and 20 cm high) consisted of three compartments, a neutral zone (15x20 cm) and sound zones 1 and 2 (20x10 cm each). At the end of the sound zone, there were holes in the wire mesh, and speakers were set behind the mesh. Ttwo nanocrystalline silicon thermoacoustic emitters were used. Each female mouse was placed in the neutral zone of the test box and habituated to the test box for 15 min in this zone. The dividers were then removed to allow the female to explore freely in the test box, including the sound zones. Once the female mouse had investigated both speaker meshes and returned to the neutral zone, USV playback was started simultaneously from both speakers. The loudness of HIGH2-4 and LOW2-4 was 264.5962.07 dB (mean dB at call start 6 sd) and 264.0162.44 dB, respectively. The playback test was			**<u>Response of female mice to the playback of USVs of selected waveforms</u>**<br>- In the first experiment, the number of entries did not show a significant difference between HIGH2-4 and LOW2-4. The duration of contact with the mesh of the speaker did display a significant difference, and females clearly preferred HIGH2-4<br>- In the second experiment, in the case of white noise and HIGH2-4, female mice showed a significantly longer duration of contact with the HIGH2-4 speaker than with the white noise speaker. A significant difference was not observed between LOW2-4 and white noise.<br>- female mice prefer HIGH2-4 to white noise, but not LOW2-4. The LOW2-4 USV had a similar effect to white noise and therefore might not have a strong aversive effect on females.

			conducted for 5 min. For the analysis, we measured the number of entries into the sound zones and the duration of investigation of the mesh. The USV file being tested was played repeatedly during the 5 min test period.			
**Author**	**Strain, Age, Number**	**Detection Method**	**Testing Condition**	**Housing conditions**	**Variables measured**	**Major findings**
Warburton et al., 1989)	Tucks Swiss T.O. mice<br><br>Exp. 1<br>16 animals of each sex<br><br>Exp. 2<br>16 young males (19-42 days old) and 16 adult females.<br><br>Exp.3<br>35 males (34-50 days old)<br><br>Exp. 4<br>16 animals of each sex<br><br>Exp. 5<br>16 animals of each sex	A condenser microphone [SMI: QMC Industrial Research Ltd. (QMC IRL), 229 Mile End Rd., London El] was suspended over the centre of the test cage, 15 cm above the wire top. Calls were detected using a S100 bat detector (QMC IRL) tuned to a centre frequency of 65 kHz with a +-5-kHz range	Surgery<br>Animals in Exp. 1 and 5 were subjected to bilateral transection of the inferior laryngeal nerves.<br><br>Behavioural tests<br>Animals designated as 'subjects' were placed in the test cage at least 15 min before placing the cage under the microphone. The isolated subject was monitored for calls for 1 rain prior to testing. If any vocalizations were detected, the test was postponed until 2 min passed without vocalization. The stimulus animal was then introduced. In Experiments 1 to 3, the stimulus females were allocated randomly to	Mice were housed in opaque plastic cages with food and water available ad lib, in a 12:12 light: dark cycle (lights out at 18:00 hr). Male and female subjects were housed 2 to 5 per cage (14x12x30 cm). Stimulus females were housed 10 to 16 per cage (24x 12×40 cm) (Experiments 1 to 3), or 2 to 5 per cage (14x 12x30		**Exp. 1 - Determination of the primary emitter of ultrasonic vocalizations in adult, mixed-sex pairs**<br>- All pairs produced high vocalization scores and there was no difference between the pairs containing males which were subsequently either nerve-transected or sham-operated.<br>- After the sham operation, ultrasound emission remained high in the control males paired with either nerve-transected or sham-operated females.<br>- No calls were detected from pairs containing a nerve-transected male.<br><br>**Exp. 2 -Ultrasonic vocalizations from prepubertal male/adult female pairs**<br>- In Experiment 2a, both the mean vocalization scores and the percentage of animals vocalizing increases steadily to reach near maximal levels when the males were about 30 days of age.<br>- Ultrasonic calls were detected from all of the pairs with naïve males first exposed to a female (Exp. 2b). But at 30 days, the mean score was significantly less than in the pairs subjected to the repeated testing procedure of Exp. 2a.<br><br>**Exp.3 - Gonadal status in relation to ultrasonic vocalizations**<br>- Exp. 3a: Ultrasonic vocalizations were detected from all groups at all ages, although there was a significant

	(42-46 days old)		subjects and used only once on each test day. The test period was divided into 36 sequential 5-see blocks and the number of 5-sec blocks containing vocalizations was expressed as a percent-age of the total.<br><br>Exp. 1<br>16 adult animals of each sex were monitored for ultrasonic vocalizations in mixed-sex pairs. Bilateral inferior laryngeal nerve transections were then performed on 8 animals of each sex, and 8 of each sex were sham-operated. Each male was paired with a nerve-transected female (Test 2) and with a sham-operated female (Test 3) in random order<br>Exp. 2<br>Young males were randomly paired with adult females and the vocalizations were monitored (exp.2a). Additional pairs were monitored during :the first exposure of young males (at either 30, 33, 36, 39 or 42 days of age;	cm) (Experiments 4 and 5). All females were virgins and stimulus females were at least 42 days old on the first day of testing. The oestrus state of females was not monitored.		increase with age (19 vs. 31 days). no significant differences in the vocalization scores either between the sexes or between the intact and neonatally gonadectomized subjects on any day. monitoring of vocalizations when the animals were returned to their home cages revealed a significant difference: from day 23, the vocalizations detected from intact males were far fewer than from the other groups<br>- Exp. 3b: The vocalization scores for pairs containing castrate males were significantly lower than for those containing intact controls. Within-subject analysis revealed a significant increase between the first and second tests for both castrate groups and for the control group tested at 34 and 42 days of age.<br>- Exp. 3c: Castration of males at 31 days of age significantly reduced ultrasonic vocalizations in tests performed 4 weeks and 8 weeks later. A subsequent test 11 weeks after castration showed no further significant change.<br><br>**Exp. 4 - The ultrasonic response of males and females to anesthetized stimulus animals**<br>- Using an anesthetized male stimulus, ultrasonic vocalizations were detected in only 2 of the 32 tests.<br>- With an anesthetized female stimulus, vocalizations occurred in 14 of the 32 tests.<br><br>**Exp. 5 - The effect of gonadal status on the elicitation and emission of ultrasonic vocalizations**<br>- Ultrasonic vocalizations were produced by all categories of subjects to all categories of stimulus.<br>- Intact male subjects were characterized by high vocalization scores in response to all stimulus categories except gonadally intact males. In contrast, the mean scores for males castrated at 31 days of age was lower than those of the intact males to all stimulus categories. For all subject categories, lowest scores were obtained when gonadally intact males were used as the stimulus.

			n=5-8 per group) to an adult female (Experiment 2b) to establish whether the repeated testing had any effect on the developmental pattern of vocalizations<br><br>Exp. 3a<br>Pups from 8 litters were allocated within 24 hr of birth to one of 4 treatment groups: intact males, intact females, neonatally gonadectomized males and neonatally gonadectomized females (8 nonlittermates per group; housed 4 per cage after weaning). At 2 day intervals from 19 to 31 days of age, ultrasonic vocalizations were monitored during 3-min test pairings of these subjects with intact, adult females. Ap-proximately 25 min after the end of each test, each subject was reintroduced into their home cage. Vocalizations were monitored during the first minute after the replacement of each animal.			

			Exp.3b<br>Males were either castrated (n=19) or sham-operated (n= 16) when weaned at 19 days of age. Ultrasonic vocalizations were monitored during test pairings of these males either at 34 and 42 days of age [Group I; n=20 (10 castrates)], or at 42 and 50 days of age [Group II; n= 15 (9 castrates)].<br><br>Exp. 3c<br>The males which previously had been repeatedly tested in Experiment 2a were either castrated (n=8) or sham-operated (n-7) at 31 days of age. Ultrasonic calls were monitored during 3-rain test pairings with an intact female on 2 consecutive days at 1, 4 and 8 weeks after castration. Animals from the castrate group were monitored again at 11 weeks.<br><br>Exp. 4<br>Half of the animals were placed in individual test cages and the other half			

			was anaesthetised and served as stimuli.<br>Half of the subjects were tested with an anesthetized male and then with an anesthetized female.<br><br>Exp. 5<br>The subjects were intact or neonatally gonadectomized males and females (from exp. 3a). And males castrated at 31 days old with no previous experience with the testing conditions. Seven categories of stimulus animals were used:<br>1) intact females (n=16);<br>2) gonadally intact females (n=16);<br>3) neonatally gonadectomized females (n=7);<br>4) neonatally gonadectomized males (n= 10);<br>5) gonadally intact females (n=8);<br>6) males castrated at 46 days of age (n=8);<br>7) gonadally intact males (n=9).			

			All but the first category had been subject to inferior laryngeal nerve transection.			
**Author**	**Strain, Age, Number**	**Detection Method**	**Testing Condition**	**Housing conditions**	**Variables measured**	**Major findings**
(Whitney et al., 1973)	Exp. I<br>F_1 cross between C57BL/6J and BALB/cJ<br>N= 52 animals (30 females and 22 males; over 70 days of age)<br><br>Exp.II<br>F_1 cross between C57BL/6J and BALB/c<br>N=24 animals (12 females + 12 males)<br><br>Exp. III<br>F_1 cross between C57BL/6J and BALB/cJ<br>N=34 (7 females and 7 males)<br>>69 days old<br><br>Exp. IV	Ultrasounds were detected with a Holgate Ultrasonic Mk. IV Receiver, set to a centre frequency of 70 kHz. The setting was selected on the basis of pilot studies with adult sub- jects of this genotype during which ultrasounds were detected only at about 70 kHz. The receiver had been calibrated against Bruel & Kjaer equip- ment consisting of a V4-in. condenser microphone (No. 4136), microphone amplifier (No. 2604), and bandpass filter (No. 1612). The	<u>Exp. I</u><br>An individual or pair of animals was placed in the experimental chamber.<br>When possible, each animal was tested in both a like-sex and an opposite-sex pairing. Thus, 11 male pairs, 22 male-female pairs, and 15 female pairs were formed.<br><br><u>Exp.II</u><br>Animals were divided in two groups:<br>- one group of 6 pairs was tested first with the female anesthetized and then tested again after a 2-day recovery period with the male anaesthetized;<br>- other group of 6 pairs was first tested with the male anesthetized	No information provided.	- Presence or absence of ultrasounds<br>- Latency to USV emission since introduction to the chamber<br>-	**<u>Exp. I</u>**<br>- Ultrasounds were detected in 2 of the 42 individual tests and during 36 of the 48 pair tests.<br>- The difference between paired and individual tests is significant, indicating that ultrasound production by adults is associated with social encounters.<br>- Virtually all of the animals appeared to be engaged in normal mouse exploration of the test chamber, yet only 2 produced detectable ultrasounds when alone in the test chamber.<br>- The 22 male-female pairs produced ultrasounds with a mean latency of 18.6 ± 6 sec. For all female pairs the value was 157.3 ± 32 sec.; male pairs had a mean latency of 207.0 ± 41 sec. Thus the same individuals exhibited a much shorter mean latency in male-female pairs than in like-sex pairs.<br>- All unlike-sex pairs produced ultrasounds during the 5-min test while only 10/15 female pairs and 4/11 male pairs produced any detectable ultrasounds.<br><br>**<u>Exp. II</u>**<br>- No ultrasounds were detected during the 12 pairings of normal females with anesthetized stimulus males.<br>- Ultrasounds were detected with a mean latency of 73 ± 35 sec. in 8 of the 10 pairings of normal males with anesthetized females.<br><br>**<u>Exp. III</u>**<br>- During Test 1, when all animals were in the normal awake condition, ultra- sounds were detected during the 5-min. test period from 15 of the 17 pairs with a mean latency of 36.6 ± 5 sec. When later tested with the male awake and the female under anesthesia, ultrasounds were detected

	F_1 cross between C57BL/6J and BALB/cJ<br><br>N=32 animals (16 females+16 males)<br>35-68 days old<br><br>Exp. V<br>N= 20 (10 males and 10 females)<br>Randomly bred heterogeneous stock descended from the intercrossing of 8 inbred strains	Holgate was sensitive to signals within about 10 kHz. of indicated frequency and was adjusted so that all scored signals were of an intensity greater than 60 db. (re 2 .0002 dynes/cm^2). The microphone was mounted 28cm above the floor of the test chamber.	and after the 2-day recovery period 4 of these pairs were retested with the female anesthetized. Two pairs were lost from the second test of the second group because the wrong pair member was anesthetized.<br><br><u>Exp. III</u><br>For Test 1, the members of a pair were simply placed together in the test chamber for 5 min. or until ultrasounds were detected. For Tests 2 and 3, 1 member of each pair was an anesthetized stimulus animal placed in the center of the test chamber and the other member of the pair was a normal awake animal.<br><br><u>Exp. IV</u><br>Each unlike-sex pair was tested once by being placed in the test chamber for 5min or until ultrasounds were detected.			from 13 of the 17 pairs with a mean latency of 68.9 ± 18 sec. However, when the female of each pair was awake and the male anesthetized, ultrasounds were detected from none of the 1 pairs.<br>- The increased incidence of ultrasonic emission when the male rather than the female member of a pair was awake is significant.<br>- The incidence of detectable ultra- sounds with only the male awake was not significantly different from the incidence of ultrasonic emission when both members of a pair were awake.<br><br>**<u>Exp. IV</u>**<br>- Eleven of the 16 pairs emitted detectable ultrasounds during the 5-min. test with a mean latency of 46 ± 14 sec. There was a difference in mean days of age between members of pairs that did emit ultrasounds and members of pairs that did not emit ultrasounds.<br><br>**<u>Exp.V</u>**<br>- On the first day of testing 8 of the 10 pairs produced detectable ultra- sounds during the 5-min. test with a mean latency of 34.4 ± 9.0 sec.<br>- Latency of ultrasound production of a pair containing a particular female on one day would have no relation to the latency of the same female tested with a different male on a different day.<br>- Latency of ultrasound production were quite consistent for individual males across days, regardless of the particular female with which they were paired.<br>- The correlation across pairs with 47 days intervening between tests was r = .68, which is similar to the value obtained when only males were considered across consecutive test occasions.

			Exp. V<br>The 10 males and 10 females were first tested in male-female pairs at about the same time each day for 10 consecutive days so that each animal was tested once in a pair with each of the 10 opposite-sex individuals. Each individual was tested once each day. The order of pairing across days and of pair testing within each day was randomized. Starting on Day 38 after completion of the first 10-day sequence, the pairs were retested in the same sequence for another 10 consecutive days.			
**Author**	**Strain, Age, Number**	**Detection Method**	**Testing Condition**	**Housing conditions**	**Variables measured**	**Major findings**
(Whitney et al., 1974)	Exp. I<br>• 10 male DBA/2J (84-95 days old)<br>• 10 male C57BL/6J (63-74 days old)<br>• 4 males geneticall	Ultrasounds were detected with a Holgate ultrasonic receiver mk IV set to a center frequency of 70 kHz. The setting was selected on the basis of prior results indicating that adult male	Exp. I<br>A test chamber was constructed of clear Plexiglas and measured 24.5 x 12.3 x 25.4 cm high. Opposite each other across the long dimension of the test chamber were startboxes 6.5 x 6.7 x 15.3 ern high. The startboxes were separated from the test	No information provided.	- Occurrence of ultrasonic calls<br>- Latency to ultrasound emission	**Exp. I**<br>- Urine from a female was less potent as a stimulus. to evoke detectable ultrasounds than was the female herself for all genotypes of male that were tested.<br>- On the first test day, all 10 DBA/2J males produced detectable ultrasounds during the 3-min test, with a mean latency of 23.95 ± 2.52 sec when tested with a female. However, only five males produced ultrasounds when tested with urine from the same female.<br>- On the second test day, only two DBA/2J males produced ultrasounds in the presence of the urine, although ultrasounds were detected from all 10 male-female pairs.

	y heterogenous (over 100 days old)<br>• 8 C57BL/6J x A7J F_1 females over 100 days old<br>• 1 I^s/Bi female over 100 days old<br>• 1 101 Bag/Rl female over 100 days old<br>Exp. II<br>• 8 DBA/2J males (103 ± 2 days of age)<br>• 8 C57BL/6J x A/J F_1 hybrids (107 days old).<br>Exp.III	ultrasounds were most intense at about 70 kHz. The receiver had been calibrated against Bruel and Kjaer equipment consisting of a %-in. condenser microphone (No. 4136), microphone amplifier (No. 2604), and bandpass filter (No. 1612). The Holgate was sensitive to signals within about 10 kHz of indicated frequency and was adjusted so that all scored signals were of an intensity greater than 60 dB (re .0002 dynes/cm^2).	chamber by metal guillotine doors. The floor of the apparatus was a table surface -covered by brown paper toweling.<br>The F_1 hybrid females were used with the C57BL/6J and DBA/2J males who were tested in alternate order on each of 2 test days.<br>For each male one of the tests was conducted in the presence of female urine and the other test was in the presence of the individual female who was the urine donor for that male on that day. Order of presentation was counterbalanced so that half the males were rust tested with a female and then tested with that female's urine; the other half of the males were tested with urine before the female. The second test day, which was 48 h later, was an exact replication of the rust test day, except that each male encountered a different female and her urine.			- The results for the C57BL/6J males were in the same direction but were not statistically significant due to low incidence of ultrasonic emission under both test conditions. C57BL/6J males produced ultrasounds on none of the 20 test occasions with urine, although ultrasounds were detected on 7 of the 20 test occasions with females.<br>**<u>Exp.II</u>**<br>- The group mean latency to ultrasound production was 8.60 ± 2.27 sec in the light condition and 8.68 ± 2.09 sec in the dark condition; there was no significant difference between conditions.<br>- Zero scores were obtained on only 1 of the 16 trials during the first 2 days of testing and on 5 of the 16 trials during the last 2 days.<br>- Zero latency scores did not occur frequently enough to invalidate the present comparison between light and dark test conditions. However, the increase in incidence of zero latency scores over the 8 days of testing was great enough to suggest that some learning may have occurred.<br>**<u>Exp. III</u>**<br>- None of the subjects emitted detectable ultrasounds during the 1-min pretest in the clean cage or during the 180-sec test in the male-soiled stimulus cage. owever, 13 of the 15 subjects produced detectable ultrasounds with a mean latency of 45.80 ± 12.63 sec when tested in the female-soiled cage.<br>- Two of the 15 subjects produced no detectable ultrasounds during the 180-sec tests in either the male or female stimulus cage. Both of these subjects were immediately tested in the presence of an adult female to see if they would produce ultrasounds to a female. Ultrasounds were detected after 110.7 sec from one of the resultant male-female pairs and the pair containing the other male produced no ultrasounds during the 180-sec test.

	• 15 male DBA/2J (130-146 days old)<br><br>Exp.IV<br>• 8 male DBA/2J		For testing with a female, the male was placed in one startbox and the female in the other startbox.<br>For testing with female urine, a cotton ball was saturated with urine from the collection receptacle of the metabolic cage and then inserted in the vial.<br><br><u>Exp. II</u><br>The apparatus was similar to the one used in Exp.I.<br>For each test, the male to be tested was carried into the prearranged (light or dark) experimental room in his home cage.<br>Each male was tested once at about the same time each day for 8 consecutive days.<br>The test trial was then initiated with introduction of the female into the male's cage. The trial was terminated upon detection of ultrasounds or after 180 sec had elapsed without ultrasounds. Upon termination of a trial, the female was returned			**<u>Exp. IV</u>**<br>- It is not necessary that males be in contact with the bedding material from female cages to produce detectable ultrasounds.<br>- Ultrasounds were never detected during the pretest with fresh bedding, although they were detected with a mean latency of 42.63 ± 19.82 sec from six of the tests when the experimental subject was in contact with soiled bedding, and ultrasounds were detected with a mean latency of 99.78 ± 27.68 sec from our of the tests when the S was not in physical contact with the soiled bedding.

			to her home cage and scores were recorded.<br><br><u>Exp.III</u><br>The apparatus was identical to that of Exp.I, except that the test chambers were 29 x 18 x 13 cm transparent plastic cages containing about 2 cm of wood chip bedding. Three types of test chamber were used. One type was a clean cage containing fresh bedding. The other two were soiled cages containing soiled bedding, in which either females or males other than the test subjects had been living for 5 days prior to the test. Eight female and three male soiled stimulus cages were used.<br>Two experimental groups:<br>- Male soiled cage before being tested in a female-soiled cage<br>- Female-soiled cage before being tested in a female-soiled cage.			

			<u>Exp. IV</u><br>The ultrasonic detection apparatus was identical to that described in Experiment I. The testing chamber consisted of a 10x 24 x 13 cm high stainless steel cage with a l-cm mesh hardware cloth bottom that was placed inside a 29 x 18 x 13 ern transparent plastic cage.<br><br>Each male was tested twice at about the same time each day on 2 consecutive days.<br>- On one test occasion each male was tested with the stainless steel chamber supported on small blocks, so that the hardware cloth floor was about 2 cm above 1em of bedding material. This distance was sufficient to preclude contact with the bedding.<br>- On the other test occasion, the chamber was positioned in the bedding material so that some of the wood chip bedding			

			protruded through the hardware cloth floor of the chamber.			

Copulation

Author	Strain, Age, Number	Detection Method	Testing Condition	Housing conditions	Variables measured	Major findings
(Sales, 1972)	3 strains:<br>• "impure albino strain (E.N.)"<br>• T. O. Swiss albino strain<br>Grey C3H strain	BAT detector and a microphone and cathode-ray-oscilloscope were used during all behavioural observations and where possible the signals were recorded at high speed on a tape-recorder responding to at least 100 kHz.	10 heterosexual encounters involving 4 males and 10 females were observed in daylight. Each male met either 2 or 3 females singly in his home age on different days. Each encounter lasted 30 minutes.	Kept individually in cages 14 x 12 x 30 cm, or in groups in cages 24 x 12 x 40 cm.<br><br>Feed: A standard diet of B 41 pellets and water was freely available to all animals and vegetable matter was given two to three times weekly	Categories of behaviour<br>- Male approach female<br>- Female approach male<br>- Male nose female<br>- Female nose female<br>- Male sniff female<br>- Male mount female<br>- Intromission<br>- Ejaculation<br>- Male groom female<br>- Male poke or bite female<br>- Male push past female	Ultrasound emission was highly correlated with "male sniff female" and "male mount" but less so with "male approach" and "male push past female" and with "male nose female".<br>The pulses emitted during mounting appeared to be related to the pelvic thrusts of the male.<br>"Audible cries" emitted by the female were often heard and recorded at the same time but were not synchronous with the ultrasounds.<br><br>Ultrasounds were recorded in 91/100 encounters.<br><br>In each strain, mounting was often accompanied by the emission of sequences of long pulses. Each sequence lasted 0,5-7.0 sec and consisted of between 3 and 25 or more pulses, each 50-300 msec in duration, emitted at intervals of up to 200 msec.<br>The calls of E. N. mice were at frequencies of 50-112 kHz, generally 60-85 kHz, whereas those of C3H mice were mainly between 40 and 70 kHz with a range of 30 to 90 kHz.<br>T.O. Swiss mice produced pulses at frequencies within the total range of 30 to 112 kHz.<br><br>These ultrasonic pulses appeared to be produced by the male mice, but ultrasound emission in adult mice is not confined to the males. Ultrasounds at frequencies between 60 and 80 kHz were

					- Rejection by female<br>- Maintenance activities<br>- Maintenance activities by both<br><br>Number of behavioural categories performed<br><br>Number of times that ultrasounds were detected simultaneously<br><br>Frequency and duration of ultrasounds	detected when an albino female from a communal cage was introduced into the cage of an isolated female and also when a non-pregnant female was introduced into the cage of a pregnant female.
**Author**	**Strain, Age, Number**	**Detection Method**	**Testing Condition**	**Housing conditions**	**Variables measured**	**Major findings**
(White et al., 1998)	Exp. I<br>5 male and 5 female B6AKF1/J<br><br>Exp.II<br>34 male and 34 female B6AKF1/J	Ultrasounds were recorded with a Bruel and Kjaer (Marlborough, MA) Model 4135 condenser microphone positioned 15 cm over the center of the test cage. It was coupled with a Bruel and Kjaer Model 2160 measuring	Exp. I<br>Ultrasonic vocalizations were recorded through one ejaculatory series. A female mouse was placed into each male's home cage and allowed to copulate.<br>Once a pair was observed to copulate, they were allowed to mate on three separate occasions, each a week apart.<br><br>Exp. II	Housed in a colony room on a 12 h:12 h normal light cycle with lights out at 1900 hours. Food and water were constantly available in their home cages, except during testing.	• Frequency (kHz)<br>• Bandwidth (kHz)<br>• Duration (ms)<br>• Relative intensity (dB)	**Exp. I**<br>- Mice emitted two distinctly different types of calls, 70- and 40-kHz.<br>- The higher frequency calls vary evenly from 10 to 150 ms in duration and the lower frequency calls vary from 10 to about 100 ms. There is little evidence for distinct clusters of calls based on duration.<br>- 40- and 70-kHz calls often occurred in the same burst of vocalizations, each call generally separated by a few milliseconds.<br>- 70-kHz calls were emitted at high rates prior to the first mount, before and during mounts and intromissions during the ejaculatory series, and before and during the ejaculatory mount. Once the intromission or ejaculation began, 70-kHz vocalization continued at a lower rate. In each set of time intervals measured, 40-kHz vocalizations

		amplifier and a Krone-Hite (Cambridge, MA) Model 3550 band- pass filter set to 20 and 100 kHz. A Lockheed Electronics (Plain- field, NJ) Store 4 tape recorder set to 30 in. (76 cm) per s was used to record vocalizations. All sound equipment used in this study had a flat response to at least 100 kHz.	Each pair was tested under one of the following four condi- tions: neither partner was devocalized (n=10), only the male was devocalized (n=9), only the female was devocalized (n=8), and both partners were devocalized (n=9). Testing took place in the male's home cage and continued until either an ejaculation occurred or the 20-min duration of the test had passed. Occurrences of both 70- and 40-kHz calls were mon- itored and recorded during the test with an event recorder, as were mounts, intromissions, and ejaculation. Mice that had not undergone devocalization were given screen- ing tests for at least 3 weeks; if they continued to copulate, they were randomly assigned to a partner and tested again.			were emitted at a lower rate than 70-kHz calls. The highest rates of 40-kHz calls were observed in the bursts occurring before and during mounts and ejaculations.<br><br>**<u>Exp. II</u>**<br><br>- Calling occurred at high levels when the male was able to call, regardless of the vocal status of the female.<br>- A small amount of 70-kHz calling occurred when the male was devocalized; however, the calling rate was not different from the condition in which both partners were devocalized.

Supplementary Table 1 – Literature reviewed

Neonatal Vocalizations

Author	Strain, Age, Number	Detection Method	Testing Condition	Housing conditions	Variables measured	Major findings
(Bell et al., 1972)	C57BL/6J<br>BALB/cJ<br>C3H/HeJ<br><br>3-18 days old<br><br>3 litters each strain<br><br>3 litters of each strain	Recordings were made inside a sound chamber (Acoustics, Inc. – Model 1200) using an Ampex (PR-500) instrumentation recorder, using a direct mode recording procedure with a tape speed of 76,8 cm/sec. The frequency response range is flat between 150 kHz and 150 kHz. The transducers employed were a Bruel and Kjaer (4135) 1/4 in. (0.64 cm) free-field condensor microphone (frequency response flat within --- 3 db, from 5 Hz to 100 kHz), a Bruel and Kjaer	All litters housed with their mothers in plastic cages (15.36 x 20.48 x 7.68 cm) with wood shavings for nesting materials. Each litter was culled to four pups within 24h after birth.<br><br>Vocalizations were recorded at D3,6,9,12,15 and 28.<br><br>Individual isolation in a glass jar (*Ambient temperature= 23.5+-1.5ºC). Recordings were made 0-10, 60-70 and 120-130 sec following placement of the pup. Individual pups were only returned to the cage until all pups in the litter had been recorded for that day.	All fitters were housed with their mothers in plastic cages measuring 6 × 8 × 3 in. high (15.36 × 20.48 × 7.68 cm) with wood shavings for nesting materials. Each litter was culled to four pups within 24 hr after birth.	Frequency<br>Mean number of signals<br>Mean peak frequency<br>Mean signal duration	Frequency of signal typically diminishes over time.<br><br>C57BL7/6J: "clicks" 10-12kHz typically followed by more sustained signals of somewhat higher frequency, many of which show the abrupt frequency shift midway during the signal of ~8kHz.<br>Rate of calling is lower at D3 compared to the other strains and the age decline in signalling is much more abrupt. Increase in signalling during the first 3min of isolation (opposite to the other strains that show a decrease).<br>Emission of briefer signals than other strains.<br><br>BALB/cJ: D3 signalling shows a rapid fluctuation in frequency across a 12kHz range. D9 consists of a signal of approx.. 0,04 sec duration in a frequency range of 50-56 kHz followed by a signal of 78 kHz peak frequency. Signalling frequency higher at D3 with a progressive decline with increasing age.<br>Generally signalling of BALB/cJ showed a lower frequency.<br><br>C3H/HeJ: Peak frequency of 76 kHz and total signal duration is appr 0.06 sec. Top signal shows a slight increase in frequency following the signal onset. Low rate of signalling at D3 and D6, an increase in signalling at D9 and a reduction in signalling afterwards.

		(2615) 1/2 in. (1.28 cm) cathode follower with a UA (0035) adaptor, and a Bruel and Kjaer (2604) microphone amplifier with a linear frequency characteristic from 10 Hz to 200kHz. Two Krohn-Hite (3550 R) filters were cascaded to provide a 48-db roll off per octave with high band pass set at 25 kHz. The microphone was placed at 0° incidence and 4 in. (10.24 cm) above the floor of the recording chamber.				
**Author**	**Strain, Age, Number**	**Detection Method**	**Testing Condition**	**Housing conditions**	**Variables measured**	**Major findings**
(Branchi et al., 1998)	CD-1<br>N=40 (4 pups of each litter – 10 litters)<br>8 days old	Ultrasonic calls were recorded using a Bruel & Kjael (B&K, Denmark) microphone Model 4135 (preamplifier	At postnatal Day 8, pups were individually removed from the litter and placed in a transparent glass beaker for testing. At the end of the test, the pup was transferred to a clean	Males and females were housed separately in groups of 8–10 in 42 x 27 x 14 cm Plexiglas	Spectrographic analysis:<br>1. Constant frequency: no change in frequency, either upwards or downwards, of	**- Mouse pups emitted a wide repertoire of US calls, both in frequency range and in signal structure.**<br>**- No difference was observed in the number of US calls emitted in the three frequency intervals. However, the five sound categories were differentially produced. Frequency steps and modulated frequency sounds were most commonly produced.**<br>**- In the low interval (42.4-58.4 kHz), frequency steps USs were mainly emitted.**

		B&K, 2633), suspended 1 cm above the beaker. Vocalizations were filtered (tunable band-pass Khron- Hite filter 3500 set at 35 to 95 kHz), amplified (B&K measuring amplifier 2610), and recorded on a Racal Store 4DS tape recorder (tape Ampex 797-15DW11) by a direct-mode recording procedure (tape speed 76.2 cm/s).	cage. After all pups had been tested, they were moved back to the cage of the mother. The experimental glass beaker (diameter 4.8 cm, height 7 cm) was inserted in a double-wall glass container (diameter 5 cm, height 9 cm). Two openings in the container walls allowed water to be pumped continuously from a water bath (+-1° C; Termomix 1420) into the double-wall container and back to the pump. This system maintained the container and the internal surface of the beaker at a constant temperature (degree of variation: +- 1°C). The temperature of the internal surface of the beaker was checked before and after every test with a Physitemp temperature-probe, model BAT-12 (accuracy level: +- 0.1^{o}C).<br><br>Four experimental conditions:<br>1. Maternal separation (isolation plus nest odor conditions): Subjects	boxes (home cages). Pellet food (enriched standard diet purchased from Mucedola, 21605 Settimo Milanese, Italy) and tap water were available ad libitum. Breeding pairs were formed and housed in 33 x 13 x 14 cm boxes.	more than 8 kHz (the choice of 16 kHz bandwidth is due to resolution).<br>2. Modulated frequency: change in frequency, ei- ther upwards or downwards, of more than 8 kHz.<br>3. Frequency steps: instantaneous frequency changes appearing as a vertically discontinuous “step” on a spectrogram, but with no interruption in time.<br>4. Composite: formed by two or more harmonically independent components, emitted simultaneously.<br>5. Short: duration less than 10 ms.	**- In the medium interval (58.4-74.4 kHz), modulated frequency and frequency steps signals were emitted more often.**<br>**- In the high interval (74.4-90.4 kHz), the USVs most commonly produced were modulated frequency signals.**<br>**- Low interval, frequency steps acoustic signals were emitted differently, depending on the condition. Subjects exposed to low temperature-isolation produced significantly more calls than tactile-stimulated pups.**<br>**- Pups exposed to male odor vocalized more than tactile-stimulated pups.**<br>**- Medium interval, isolated pups emit- ted more frequency steps signals than tactile-stimulated pups.**<br>**- High-frequency interval, the low temperature-isolation group produced more modulated frequency signals than tactile-stimulated pups.**

			were exposed to two experimental conditions. First, the pups were left undisturbed (isolation) and, immediately afterward, the subject was moved, without hand contact, to a second beaker (nest odor), the bottom of which was covered with a 0.5- cm layer of sawdust taken from the nest cage.<br>2. Low temperature-isolation: The subjects were exposed to a temperature of 22 +-2° C, about 10 – 12°C less than the temperature normally recorded in the nest.<br>3. Tactile stimulation: The pups were exposed to a tactile stimulation provided with a sable-hair brush (Sullivan & Leon, 1986) by the experimenter.<br>4. Male odor: The subjects were placed in a beaker with the bottom covered with a 0.5-cm layer of saw- dust coming from the cage of an adult conspecific male, CD-1 strain, different from the natural parent.			

Supplementary Table 1 – Literature reviewed

Author	Strain, Age, Number	Detection Method	Testing Condition	Housing conditions	Variables measured	Major findings
(D'Amato and Populin, 1987)	Multiparous females of the GFF strain	Ultrasounds (70 ±5 kHz) were recorded by the use of a bat detector (QMC Instruments) in isolation and at room temperature for a 5-min period (15 s after having removed the pup from its home cage). During this test the pup was placed on a selective activity meter (ANIMEX) to record locomotor activity simultaneously.	Coss-fostering procedure, between deaf mice and mice with normal hearing, was applied within 24 h from birth. Four groups of mice constituted the subjects of this study: (1) deaf mothers with their own pups (D-D; N = 6), (2) deaf mothers with normal-hearing pups (D-NH; N = 6), (3) normal-hearing mothers with their own pups (NH-NH; N = 6), and (4) normal-hearing mothers with deaf pups (NH-D; N = 5).<br><br><u>Pup developmental indices</u><br>Each day (from day 2 to 15) pups were weighed and tested for their sensory-motor development, according to the following measures: righting, visual cliff, placing, vibrissae, grasping, and bar holding. The age at which a given response was present in its adult form in, at least, 50% of all pups was used as the	housed in a 33.2 • 15 x 13-cm-high plexiglass cage, the floor of which was covered with wood chips. Food and water were available ad libitum. Throughout the experiment the cages were maintained in a soundproof cabin at a constant temperature (23 _+ 2~ Animals were kept on a 12-h light/12-h dark, partially inverted, cycle, the light being switched on at 1300 h and off at 0100 hr. For a 15- day period a male belonging to the same line	<u>Pup developmental indices</u><br>- Weight<br>- Righting<br>- visual cliff<br>- placing,<br>- vbrissa<br>- graspin<br>- bar holding.<br><br><u>Behaviour of the mothers</u><br>- in nest<br>- nursing posture<br>- groomin g pup<br>- nest building<br>- feeding activity<br>- self-groomin g, locomoti on<br>- explorati on of the physical environ ment<br>USVs<br>- Number of USVs	**<u>Pup Behaviour</u>**<br>- The amount of ultrasonic vocalizations emitted by the pups was strongly reduced by deafness, was affected by the cross-fostering procedure and changed with the age of the pups.<br>- ups with normal hearing showed a strong reduction in the number of ultrasounds emitted when reared by a deaf mother.<br>- Ultrasounds emitted by deaf pups seemed to be unaffected by the presence of a normal mother.<br>- Individual between- groups comparisons revealed that pups with normal hearing reared by their mother emitted, from day 3 to day 7 of their life, a significantly greater number of ultrasounds than all other groups.<br>- Deaf pups displayed a greater amount of locomotor activity than pups with normal hearing.<br>- Deaf pups, contrary to normal pups, showed an increase with age in locomotion scores.<br><br>**<u>Pup Development Indices</u>**<br>- No apparent differences were found in the time of reflex appearance between deaf and pups with normal hearing.<br>- Only righting and the vibrissae performances seemed to be delayed by the cross-fostering procedure, but no interaction effects were discovered.<br>- Deaf pups were heavier and their weight gain was greater in comparison with pups characterized by normal hearing.<br>- Cross-fostering pups showed a different body weight increase, according to the genotype of the mother, with deaf mothers facilitating and normal hearing mothers reducing the amount of weight gained by pups. Individual between-groups comparisons shows that each unfostered group significantly differed from all three groups, from the seventh day (D-D) and from the third day of pup life onward (NH-NH)<br><br>**<u>Behaviour of the mothers</u>**

			representative score for each litter.<br><br><u>Behaviour of the mothers</u><br>Behaviour of the Mothers. From day 2 to day 15, females were observed for a 30-min period, 6 days a week, between 0900 and 1100 h. Female behaviour was recorded once a minute, for a total of 30 sampling points a day, according to the following behavioural categories: in nest, nursing posture, grooming pup, nest building, feeding activity, serf-grooming, locomotion, and exploration of the physical environment. In the analysis of results, data were grouped into two blocks, according to the age of pups (first week, from day 2 to day 8; and second week of data col- lection, from day 9 to day 15).	(dn/dn or + / +) was housed with the female subject. On the 15th day the male was removed and the female was provided with a fixed amount of paper to build her nest.		- Deaf mothers showed an increased locomotor activity.<br>- The cross-fostering procedure decreased the number of intervals spent in the nest and in the nursing posture, while it increased feeding activity and self-grooming.<br>- Feeding increased during the 2nd week of the pups' life, while all other parameters of maternal behaviour diminished.<br>- Pup age had no apparent effect on locomotor activity, self-grooming and exploration.
**Author**	**Strain, Age, Number**	**Detection Method**	**Testing Condition**	**Housing conditions**	**Variables measured**	**Major findings**
(D'Amato et al., 2005)	Exp. I<br>NMRI<br>N=60<br>60-70 days old	Bat detector (MINI-3) set on the 70 ± 5 kHz placed about 10 cm over the	<u>Exp.I – Maternal Responsiveness</u><br>The experimental apparatus consisted of a Plexiglas cage (40 [illegible] 23 [illegible]	Animals (22–24 gr.) were housed in groups of 10–12 in Plexiglas	Exp. I<br>Behavioural measures:<br>- Latency to leave	E**<u>xp. I – Maternal responsiveness</u>**<br>- The presence of alien, in comparison with own pups, increased the time the mother needed to reach the litter due to an increase in the latency to leave the starting

	Exp. II<br>24 females and 12 males C57BL/6<br>20 females and 10 males BALB/c<br><br>30 females and 14 males NMRI	pup. The bat detector was connected to a PC equipped with the ''Ultr- avox'' software (Noldus, The Netherlands).	15 cm) divided in three equal compartments (A, B, and C) by two transpar- ent Plexiglas partitions. Each partition was provided with 16–18 holes (0.3 cm diameter) allowing visual, auditory, olfactory information to be gained from the compartments. It was possible to open a larger hole in each partition (door: 2.5 cm diameter), located 2 cm from the lateral border, and 5 cm from the bottom; this allowed the mouse to move from one compartment to another. Doors in the experimental apparatus were located the first on the left (in the first partition), the second on the right (in the second partition).<br>On postnatal day 8 the entire litter of a female was transferred into the experimental cage, in com- partment C, far from the door. The doors were closed. A naï̈ve male mouse was, concomitantly introduced into the compartment B (the one in the middle). The	cages (40 x 23 x 15 cm) in a colony room with constant temperature (21 ± 1 °C) and maintained in a 12/12-h light/dark cycle (light on at 7:00 hours). Pellet food and water were available ad libitum.		the A compartment (A-B)<br>- Latency to reach pups (total time)<br>- Time spent in the different compartments (Time in A and time in B)	compartment and in the overall time spent by mothers in it.<br>- Morphine administration had similar effects on the total time needed by the dams to reach their pups in comparison to Saline injected mothers. No effects were found in either parameter when naltrexone was administered.<br><br>**<u>Exp. II – Maternal responsiveness and pups' ultrasonic calls</u>**<br>- BALB/c mothers showed a higher latency to pass in the compartment B in comparison to C57BL/6 mothers and on the whole spent more time in the starting compartment in comparison with C57BL/6 mothers.<br>- C57BL/6 pups uttered fewer calls than BALB/c pups both at postnatal day 4.<br>- C57BL/6 pups spent less time vocalizing than BALB/c pups both at postnatal day 4.<br>- Maternal responsiveness was also higher in mother whose pups underwent the handling proce- dure during the first days of life as suggested by the decrease in the time spent to reach pups by the mothers in the handled group.<br>- Handled pups uttered fewer calls than their controls at day 8 but not at day 4.<br>- No differences on UVS duration were found between the two treatment groups.

			experimental cage was placed on a hot plate apparatus set at a temperature of 35 °C to prevent pups' cooling, for 30 minutes. The mother was left in its home cage during this period. After 30 minutes, the male mouse was removed, the doors were opened, and the mother was introduced into the compartment A (time 0). The test ended when the female entered compartment C. Experimental groups: In the first experiment the effect of the presence of own versus alien pups was evaluated exposing females to their offspring (OWN, n=7) or same- age offspring of an unfamiliar lactating NMRI dam (ALIEN, n=9). In the second, the effect of morphine (MO 2.5 mg/kg i.p, n=7) and naltrexone (NTX 1.0 mg/ kg, i.p, n= 8) were evaluated, injecting the dams 30 and 15 minutes, respectively, before the test.			

			Exp. II – Maternal responsiveness and pups' ultrasonic calls<br>NMRI litters were randomly assigned to one of the two experimental conditions on day 1: postnatal manipulation (handled), and unhandled. Once a day, from day 1 to 14, each whole handled litter was transferred to a new cage, the floor of which was covered with clean bedding and left for 15 minutes. During the entire 15 minutes of the procedure, the cage was placed on a hot plate set at a temperature of 35 °C, to prevent cooling of pups. During this procedure the mothers were left in their home cages.<br>Pups USVs: Only one pup per litter was tested for ultrasonic emission on day 4 and on day 8. The procedure was as follow: the mother was firstly removed from the cage and, after 5 minutes one randomly chosen pup was put in an empty glass at room temperature (22 ± 2 °C) and its ultrasonic			

			emission was measured for 5 minutes.<br>On day 8, one randomly chosen pup was tested during the isolation of the mother, preceding the maternal responsiveness test.			
**Author**	**Strain, Age, Number**	**Detection Method**	**Testing Condition**	**Housing conditions**	**Variables measured**	**Major findings**
(Ehret and Bernecker, 1986)	NMRI<br><br>0-18 days old<br><br>8 females of each exp group each with litters of 7 pups (standardized at delivery – adding or culling)	An ultrasound detector (improved version of Andersen & Miller 1977) was used to detect any sounds made by the pups in a frequency band between 30 and 90 kHz. The microphone was mounted 12 cm above the litter.	Observations were made in the home cage (pups + dam) inside a soundproof anechoic room between 0700-1300h.<br>4 experimental groups:<br>- Bilaterally deafened females_BLD (n=8)<br>- Unilaterally deafened females_ULD (n= 8)<br>- Normal hearing females (n=8)<br>- Normal hearing females with paralyzed pups_NPB (n=8)<br>Controls for the occurrence of ultrasounds and pain calls:<br>3 normal mothers with standardized normal litters	Standard cages (26,5x20x14cm)<br><br>12h light-dark photoperiod<br><br>Av. Temperature of ~ 18C in January, February and March and ~25C in June, July and August	- Litter weight<br>Wriggling calls (WC) produced by pups: total number; n followed by a change of behavior from the dam (licking of pups, changing suckling position, nest building, “attention”); n that were not followed by response from the dam, n of activities without preceding WC (spontaneous maternal behavior)	Litter weight<br>● At birth, no differences between normal hearing and ULD mothers or between normal hearing and BLD mothers. Litters of ULD mothers significantly heavier than BLD mothers.<br>● Slower increase in weight in experimental (ULD+BLD) litters than normal hearing.<br>● From day 2, ULD litters significantly heavier than BLD litters. Normal hearing litters significantly heavier than BLD litters.<br>● No sig differences between litters of normal hearing females and ULD 1 day after birth.<br>● Average day-to-day weight gain larger for litters of normal hearing and ULD litters than BLD litters.<br><br>Control measures<br>● No single pure ultrasound recorded while suckling at least part of the litter.<br>● Pain calls w/ high ultrasonic components were recorded only 3 times.<br><br>General behavioural observations<br>● First 5 days after birth, females often remained w/ litters during 30-min observation period.<br>● From D6, breaks in feeding the pups occurred more often (increasing w/ age of the pups).<br>● Pups emitted wriggling calls when the mother was in a suckling position on the litter.

						● Wriggling calls were always associated with movement of the pups<br>● Pups older than 5 days old also emitted calls when they pressed and crawled over each other in the nest in the absence of the mother.<br><br>Wriggling call production and responses of the mothers<br>● No differences in the rate of wriggling calls between groups<br>● Call production rate increased significantly from birth to a broad plateau from D4 to D13. Decreasing between D13-16, reaching values similar to D0.<br>● Spontaneous maternal behaviours were similar in all three groups except a few days after birth BLD mothers showed significantly more spontaneous maternal behaviour than normal (D2,6,8) and ULD (D3).<br>● Rate of spontaneous maternal behaviour decrease significantly in the three groups from birth to D5 and reached a plateau afterwards.<br>● Rate of maternal activities in response to wriggling calls – increased between birth and day 5 in normal mothers and ULD mothers.<br>● Average n of maternal responses to wriggling calls : Normal mothers>ULD>BLD<br>● Rate of apparent “responses” after wriggling calls was almost equal to the predicted n of coincidences between maternal activity and preceding wriggling call production in BLD females. This was smaller in ULD and normal females.<br><br>Specificity of responses to wriggling calls<br>● Significantly more licking (D0-5) and nest building (D0-7) in normal and ULD females.<br>● Normal and ULD females showed more attention to wriggling calls between D2-15.<br><br>Responses of normal females to playbacks of wriggling calls<br>● NPB mothers (females w/ paralysed pups) responded significantly more often with maternal behaviour (licking

						and changing position) and with attention compared with BLD mothers; (b) NPB mothers showed significantly less call-induced licking and nest building compared with normal females; and (c) NPB mothers showed significantly more spontaneous maternal behaviour than normal and ULD mothers but there was no difference when compared with BLD females.
**Author**	**Strain, Age, Number**	**Detection Method**	**Testing Condition**	**Housing conditions**	**Variables measured**	**Major findings**
(Elwood and Keeling, 1982)	CSl mice<br><br>D1-13<br><br>3 litters of 4 pups each (surplus culled at the day of birth)	Microphone of a QMC SlOO ultrasonic detector was suspended 7 cm above the pup and connected, via the high-frequency output of the detector, to a Racal Store 4D high-speed tape recorder. This was set at 30 in./sec, enabling frequencies of up to 150 kHz to be recorded.	Recordings initiated on D1 and continues in alternate days until D13. Isolation in a glass dish maintained at 20ºC	No information provided	Call rate	No significant difference between litters.<br>Peak ultrasound production at D7<br>The majority of US calls occur in bouts of 2 or more calls.<br>D3_ only 2% of the calls occurred singly. On D13 it rose to 38%.<br>The mean bout length was maximal on D3 and minimal on D13.<br>During the 1st week, bouts of 20 or more calls were common, with the longest bout occurring on D3.<br>The mean number of bouts rose from D1 to D5 remained stable on D11 and dropped on D13.
**Author**	**Strain, Age, Number**	**Detection Method**	**Testing Condition**	**Housing conditions**	**Variables measured**	**Major findings**
(Hahn and Schanz, 2002)	169 mouse pups from 29 litters<br><br>B19DBAF1/J (87 pups from 15 litters)	Bruel and Kjaer (B & K) Type 4135, 1/4 inch (6.4 mm) microphone, a B & K Type 2619T preamplifier, a B & K Type 2606	**Cool Testing**<br>Each pup was placed individually on a cotton pad inside an aluminum weighing dish that sat atop about 100 ml of ice in a 250-ml beaker. The surface of the cotton	Mice were maintained in transparent colony cages with stainless steel tops. The cages were 30 □ 20 □ 15 cm	- Number of calls	- Rotation appears to be a powerful stimulus in eliciting ultrasonic calls in comparison with a cool temperature, especially in the SJL F1 hybrid mice, in which it more than doubled the number of calls per second. Within situation, DBA F1 mice appear to produce more ultrasounds than SJL F1 animals. It also appears that on some days (e.g., days 5 and 6), situation and genotype interacted. Finally, by day 8 the rate of calling was dropping rapidly for all

	B10SJLF1/J (82 pups from 14 litters)<br><br>2-8 days old	measuring amplifier, and a Teac instrumentation tape recorder. Using high-quality video cassette tapes and a recording speed of 76 cm/s, we obtained a frequency response on taping of 150 Hz to 150 kHz.	pad was maintained at between 10° and 11°C. The beaker was placed in a dark, sound-attenuated chamber where the air temperature was about 21°C, and the B & K microphone was located above the beaker, about 5 cm away. Recording began immediately and continued for 20 seconds (about 1500 cm of tape). Each pup was recorded individually in that cool, isolated environment on days of age 2 through 8. The cotton pad and aluminum dish were changed between litters.<br><br>**<u>Rotation Testing</u>**<br>Each pup was placed individually into a 250-ml plastic beaker that was set on a 45-degree angle and rotated at 10 rpm. As the beaker rotated, the pup would roll over on to its back and have short falls. The slowly rotating beaker was housed in a dark, sound-attenuated chamber where the air temperature was about 21°C, and the B & K	in dimension. All mice were fed a diet of Agway RMH 3000 animal chow. Food and tap water were available at all times. The colony was maintained on a 12:12 hr, light/dark cycle with lights on at 0800.		mice. Mice tested in the cool situation dropped to fewer than one call per second on average, while those mice in the rotation situation were still calling at about two calls per second.<br>- SJL F1 hybrid mice producing more calls than might have been expected in the rotation situation on days 2 and 3 and producing fewer calls than might have been expected in the cold situation on days 5 and 6. Those interactions, while statistically re- liable, accounted for little of the total sums of squares, an average of 2.7% over the 7 days.

Author	Strain, Age, Number	Detection Method	Testing Condition	Housing conditions	Variables measured	Major findings
			microphone was located above the beaker, about 5 cm away. Recording began immediately when the beaker began to rotate and continued for 20 seconds (about 1500 cm of tape). The rotating cup was changed between litters.			
(Hahn et al., 1997)	C57BL/10J<br>DBA/2J<br>BALB/cJ<br>SJL/J<br><br>N=246<br>3 days old<br>Male and female	Ultrasonic vocalizations were recorded with a Bruel and Kjaer 4135 0.635-cm microphone, a B-K 2619T preamplifier, a B-K measuring amplifier, and a Precision Data PI 6204 instrumentation tape recorder. Using Maxell UDXL 50-60 recording tape and a recording speed of 95.25 cm/s (37.5 in./s), we recorded with a frequency response of 150 Hz to 150 kHz.	At the time of testing, the entire litter was removed and placed into a 250-ml plastic beaker. Each pup to be tested (about three per litter) was chosen at random from the beaker and placed onto a cotton Nestlet (30 X 30 X 5-mm piece of cotton) inside an aluminum weighing dish. The dish was placed in a 250-ml beaker on top of about 200 ml of small ice cubes.	Breeding animals were housed in a standard polycarbonate breeding cage with pine shavings and a single cotton Nestlet. Each cage had food (Agway RMH 3000) and tap water freely available. Each breeding pair remained together during mating, gestation, and the rearing of off- spring. Each cage was checked once a day (at about 0900), 7 days a week	- Rate of calling (calls/second for 18s)<br>- Beginning frequency<br>- Ending frequency<br>- Highest frequency<br>- Lowest frequency<br>- Length of call	- No significant effect of sex or a sex x strain interaction on any of the call characteristics that were measured.<br>- Strain was a significant factor on the rate of calling.<br>- For calling rate, Three categories or groupings were obtained, namely, BALB > DBA > C57 = SJL.<br>- In the DBA and BALB strains, 25 of 26 animals produced calls. In the C57 and SJL strains, 8 of 27 animals produced calls.<br>- Strain was a significant factor for the 4 frequency parameters measured.<br>- BALB strain exhibited lower pitched calls than the other three strains.<br>- Strain has a significant effect on call length.<br>- BALB and DBA strains produced linger calls than C57 and SJL strains.<br>- BALB strain produced the highest rate of calling with calls of longer duration and the lowest pitch. DBA strain mice produced fewer calls of a similar duration and higher pitch. Mice of the C57 and SJL strains produced the fewest calls and they were shorter in duration and higher in pitch than those of the BALB strain.<br>- Hybrids produced calls at a significantly higher rate than did inbreds. The calls of hybrids were also significantly longer than those of inbreds. And, exhibited a wider frequency range in their calls; their calls began lower and ended lower while having a highest frequency that was not different from the calls of inbreds.

		The microphone was placed vertically above the pup, about 50 mm away. For recording, the micro-phone and cup containing the pup were inside a sound isolating chamber measuring about 50 cm in each dimension		for births (day the litter was found = 0 days of age). Colony and testing rooms were maintained at 21 ± 1°C on a 12/12 light/dark cycle, with lights on at 0800.		- F, hybrids between two inbred groups had rates of calling that approached or exceeded the value of the higher scoring inbred parent group.<br>- Directional dominance and additive variation together account for 35.7% of the total variance in rate of calling.<br>- The beginning frequencies of the calls of hybrids were intermediate to their inbred parent strain values but more closely resembled the parent strain with the lower beginning frequency.<br>- Both additive and directional dominance components are significant for beginning frequency.<br>- Hybrid pups produced calls that had a pitch inter-mediate between their inbred parent values but that more closely resembled the inbred parent strain with the lower pitch.<br>- Neither maternal effects nor other reciprocal effects reach statistical significance for lowest frequency and ending frequency. In both cases, additive and directional dominance components together, explain just over 18% of the total phenotypic variance.<br>- Regarding call length, hybrid values fall between their inbred parent strain values but resemble the parent strain exhibiting longer calls. either maternal nor other reciprocal components reach statistical significance. Additive and directional dominance components explain just over 18% of the total phenotypic variiance in this character.
**Author**	**Strain, Age, Number**	**Detection Method**	**Testing Condition**	**Housing conditions**	**Variables measured**	**Major findings**
(Hahn et al., 1998)	486 male and female mice (equal numbers) 2-12 days old<br><br>complete intercrossing of four	USVs were recorded using a Bruel and Kjaer (B&K) 4135 0.25h-in. (6.4-mm) microphone, a B&K 2619T pre-amplifier, a B&K Type 2606	At the time of testing, the entire litter was re-moved from the nest and placed into a 250-ml plastic beaker at room temperature (about 21°C). One at a time, the pups were removed from the beaker without regard to order,	All animals were maintained in transparent colony cages with stainless steel tops. The cage dimensions were 30 X 20	-Rate of calling (calls/second for 18 s)<br>- Length of call,<br>- Beginning frequency of call<br>- ending frequency of call,	**<u>Call eliciting procedures</u>**<br>- In most cases the change in procedure resulted in a smooth transition from day 7 (the last day of cold-induced calls) to day 8 (the first day of handling-induced calls).<br><br>**<u>Developmental analysis</u>**<br>- A greater percentage of hybrid animals emitted calls on days range.

	inbred strains (a 4 X 4 diallel cross) using the C57BL/10J, DBA/2J, BALB/cJ, and SJL/J inbred strains.	measuring amplifier, and a Precision Data PI 6204 instrumentation tape recorder. Using Maxell UDXL 50-60 recording tape and a recording speed of 37.5 ips (0.938 m/s), a frequency response of 150 Hz to 150 kHz was obtained. characteristics of ultrasonic vocalizations were measured using a Kay Elemetrics 5500 Digital Sonagraph sound spectrum analyzer. Playback of tapes at 3.75 ips (93.8 mm/s), 1/10 recording speed.	identified, and placed into the recording chamber.<br>Procedure for pups aged 2-7 days: the pups were placed on a cotton pad inside an aluminum weighing dish atop about 100 ml of ice in a 250-ml beaker. The surface of the cotton was maintained at between 10 and 11°C. The beaker was placed in a dark, sound-attenuated chamber where the air temperature was about 21 °C, and the B&K microphone was located above the beaker about 5 cm away. Recording startedimmediately and continued for 18s (about 1500 cm of tape). Each pup was recorded individually in that cool, isolated environment each day, from 2 to 7 days of age.<br>Procedure for pups aged 8-12 days: USVs were elicited by a mildly stimulating handling procedure that was similar to the "falling" procedure employed by Okon (1970b) and that likely simulates mild	X 15-cm. Breeding pairs were fed a diet of Agway RMH 3000 animal chow. Food and water were available at all times and the colony room had a 12:12-h light:dark cycle, with lights on at 0800h.	- highest frequency,<br>- lowest frequency, and<br>- frequency range (highest-lowest frequency).	- Rate of calling shows an increase to about day 5 and a decline over the remaining days. There is a gradual decrease in the length of calls as the animal ages.<br>- Beginning, ending, and lowest frequency of the calls all show an increase with age.<br>- Call length and beginning frequency, were influenced by the sex of the subject.<br>- The inbred—hybrid effect persisted through 9 days of age.<br>- Relative to inbred animals, hybrids had a higher rate of calling, longer calls, and lower call frequencies with a greater range.<br>- There was a significant sex effects on rate of calling, on days 4 and 5, when inbred and hybrid females emitted fewer calls. On days 3, 6, and 8 males produced calls with a greater frequency range than females.<br>- Males produced longer calls from days 3 to 7.<br>- Males had calls of a lower frequency than females on days 4 and 5.<br>- Inbred and hybrid males exhibited a greater frequency range in their calls.

			handling during parental brood care. We placed each pup in a 250- ml beaker held at 45° and rotated the cup by hand for 18 s at 0.5 rev/s. This procedure elicited pure ultrasonic calls only.			
**Author**	**Strain, Age, Number**	**Detection Method**	**Testing Condition**	**Housing conditions**	**Variables measured**	**Major findings**
(Henessy et al., 1980)	Experiment I: C57BL/6J (C) and A/J (A)<br><br>Cross-fostering<br><br>3-9 days old<br><br>Number?	USVs: Ultrasonic detector (Mark V; Holgates of Totton, England) equipped with preamplified microphone and adjustable frequency tuner (bandwidth = *5* **kHz).** Signals processment: solid-state conditioner whose output was recorded by a counter. The conditioner determined the presence or absence of ultrasound during discrete successive intervals of about 75 msec each. The	Experiment I – Post-handling mother-pup interaction:<br>On the day of birth (D1), pups were redistributed among females. 4 experimental groups were formed:<br>1) C mothers given C foster pups (C/C)<br>2) C mothers given A foster pups (C/A)<br>3) A mothers given C foster pups (A/C)<br>4) A mothers given A foster pups (A/A)<br>Observations of maternal behaviour D3, ,5 , 7, 9 and 11 post-partum. Removal of pups from the cage and isolation for 2 min in a 500mL glass jar with wooden chips. Followed	Single housed females (4 days before parturition) in individual clear plastic cages (28.4 X 17.8 X 13.3 cm) lined with wood chip.	EXP. I<br>- Pre-handling behaviour observation s (D3,5,7,9 and 11 postpartum) : out of nest, nursing, licking pups, nest-building, self-grooming, rearing feeding and drinking, number of pups out of the nest.<br>- Post-handling behaviour observation s: latency to retrieve 1st pup, nursing, pup-licking,	Experiment I:<br>Pre-handling observation<br>- Dams were observed to nurse more often and to be out of the nest and self-grooming less often when caring for their own strain<br>- Nursing observed more often on the earlier days of lactation<br>Post-handling observations<br>- C dams retrieved faster and were observed to be nest-building more often (C/C + C/A)<br>- C dams were observed to be nursing, pup carrying and sel-grooming (C/C + C/A)<br>- Dams of both strains initiated retrieval faster if they had A foster pups and licked C pups more often.<br><br>Experiment II:<br>- First 4 min following handling, C pups only signalled occasionally<br>- In half of the sessions (20/40), C litters did not produce detectable USVs<br>- USVs were consistently detected in AC pups on all test days<br>- Significative differences between USVs emission between A and C pups<br>- Strain of the mother did not have an effect on USVs production<br>- Following return of the dam to the cage, all A litters showed a reduction in signalling. C pups emitted slightly less USVs than A pups.

		frequency tuner was set at 68 kHz.<br>Microphone positioned 12 cm above the pups.<br>Axillary body temperature: Microprobe thermometer (BAT-4; Bailey Instruments, New Jersey)	by this, a pup retrieval test was performed.<br><br>Experiment II:<br>Animals were tested on D3, 5, 7 and 9 . Pups were handled as done on Exp I. Axilliary temperature was measured just prior to and immediately after being handled.		in nest with all pups, nest building, self-grooming, pup carrying.<br><br>EXP II<br>- Pup axillary temperature;<br>Number of ultrasounds during 4min (?)	Body temperature: C pups showed a progressive resistance to temperature loss while out of the nest and clear increase in temperature during the 15min following replacement in the nest and the return of the mother to the cage, across test days. No such effect was observed in A pups. Re-exposure to A dams resulted in higher pup temperature than re-exposure to C dams. Prior to re-exposure, maternal strain had no effect.
**Author**	**Strain, Age, Number**	**Detection Method**	**Testing Condition**	**Housing conditions**	**Variables measured**	**Major findings**
(Marchlewska-Koj et al., 1999)	Strain: CBA & C57BL<br><br>On D0, litters were reduced to 5 or 6 animals (3 females and 3 males)<br><br>CBA pups: N=45<br>D1, 3, 5 or 7<br>To investigate the influence of the presence of different beddings, D3	Ultrasound receiver suspended 5 cm above the tested animal. The microphone was connected to a QMC ultrasound detector, type S25, coupled to a cassette recorder (Sony, HX PRO).<br><br>Sound analysis: Canary program (Cornell Bioacoustics Workstation, Version 1.2).	Each pup was tested only once.<br><br>**Isolation procedure (common to both exp. conditions)**<br>- An individual pup was removed from the tome cage, placed on a plastic dish, and kept warm for 10min. Then it was either observed with no bedding or with approximately 2cm3 of the selected bedding material placed on the dish next to the pup. The tested	Info only provided pre-pairing.<br><br>The animals were maintained in unisexual groups in polyethylene cages (28 ´ 23 ´ 10 cm) and fed a standard pellet diet (Motycz, Poland) and water. The ground wood shavings used for	Number of calls<br>Duration of calls<br>Frequency (kHz)	Pups of CBA-strain mice produced ultrasound calls when they were taken out of the home cage and cooled.<br>The fundamental frequency was higher in 7 day old pups than in 5 days old pups.<br><br>**Type of bedding**<br>Number and duration of calls were higher in pups exposed to C57BL bedding than in pups exposed to clean bedding, home bedding or bedding from CBA unrelated nests.<br><br>**Postnatal experience**<br>The number of ultrasounds was modified in the presence of bedding. CBA mice by their own moher or fostered by C57BL females produced significantly more calls in the presence of C57BL bedding than when exposed to bedding of CBA lactating females.<br>The duration of calls was longer in pups kept with their own mothers or fostered by C57BL lactating females and exposed to C57BL bedding than in those tested in the presence of CBA bedding.<br>The frequency of calls emitted in the presence of CBA bedding was modified by nursing conditions. Pups nursed by their own mothers

	pups were used	The number of calls per min was estimated for each sample.	animal was then introduced to an acoustically isolated chamber with an ambient temperature of 18-20C. Recording started 1min after the bedding was presented.<br><br>**Influence of different beddings**<br>- 45 3-day-old CBA pups nursed by their own mother were assigned<br>- 4 groups:<br>- Clean bedding<br>- Home bedding<br>- Bedding from an unrelated CBA nest<br>- Bedding from a nest with a different genotype.<br>**Influence of postnatal experience**<br>- 21 CBA pups fostered by C57BL females for two days were tested at 3 days of age.<br>Exposed to the bedding of their own CBA mother or bedding of their foster mother.	bedding were changed weekly. The animal room was kept in a 14:10 light/dark cycle initiated at 7:00 a.m., at 21-25° C and 20-60% relative humidity. Two weeks after arrival the mice were paired, one male per female.		vocalized at higher frequencies than did newborns fostered by C57BL females. The effect was similar when pups were exposed to C57BL bedding.<br>Frequency of calls of pups kept with their mothers were higher than those from mice nursed by C57BL females, but the difference was not statistically significant.

Supplementary Table 1 – Literature reviewed

Author	Strain, Age, Number	Detection Method	Testing Condition	Housing conditions	Variables measured	Major findings
(Mogi et al., 2017)	ICR<br>Exp. I<br>17-21 days old<br>24 males<br>23 females<br><br>Exp. II<br>20 females<br><br>Exp. III<br>23 females<br>5 day old pups<br><br>Exp. IV<br>12 females<br>5 day old pups	Ultrasound recording was performed using a condenser micro- phone (UltraSoundGate CM16/CMPA, Avisoft Bioacoustics, Berlin, Germany) as previously described.	Exp. I<br>A two-choice test was employed in which mice selected two directions in parallel rows.<br>A subject 17-21 days old pup was introduced into the neutral area of the empty testing apparatus with the mesh doors left open and the narrow gates between middle and dam areas closed. The pup was shut in the neutral area by closing the mesh door, and the biological mother and alien mother, the latter of which had likewise reared pups for 17–21 postnatal days, were introduced into the dam areas, separately. Next, in order for the pup to smell the odour of the mothers, narrow gates were opened and airflow was produced by the fan for 10 min.<br><br>Exp. II<br>a mother was introduced into the neutral area of the testing apparatus with the mesh doors open and allowed to acclimate to the neutral	Mice of each sex were housed in groups of 3–5 individuals per cage. The environment was maintained at a constant temperature (24 ± 1 ◦ C) and humidity (50 ± 5%) under a 12-h light-dark cycle (lights were switched on at 0800). Food and water were supplied ad libitum. Pregnant females were individually housed in breeding cages (24.5-cm long × 17.5-cm wide × 12.5-cm tall), and checked daily in the morning until parturition.	Exp. I<br>- Latency of entry into each dam area<br>- Contact with each mother<br>- Duration of time spent in each dam<br>- Time in contact with mother<br><br>Exp. II<br>- Latency of entry into each pup area<br>- Contac with each bottle<br>- Duration of time spent in each pup area<br>- Time in contact with each bottle.<br><br>Exp. III<br>- Latency of entry into a tube<br>- Duration of tube stay<br>- Duration of mesh search<br><br>Exp. IV	**Experiment I**<br>- The time spent in the biological dam area was significantly longer than time in the alien dam area, between 10–20 min after the start of the experiment. There was a significant effect for mother group but not for sex or time course.<br>- Time in contact with the biological mother was significantly longer than for the alien mother, between 10–30 min after the start of the experiment. There was a significant main effect of mother group but not for sex or time course.<br>- Mouse pups distinguish between different mothers and prefer their biological mothers over alien mothers.<br>- Average latencies of both entries into biological dam area by pups and contact with their mother were more than 8min and there were no effects for either sex or mother group on the latencies of entries into each dam area and contact with each mother.<br><br>**Experiment II**<br>- The time spent in the alien pup area was significantly longer than the time spent in the biological pup area, between 10–30 min after the start of the experiment.<br>- The time spent in contact with bottles containing an alien pup was significantly longer than that with bottles containing a biological pup, between 10 and 20 min after the start of the experiment.<br>- The findings suggest that mouse mothers can distinguish their biological pups; however, it cannot be inferred that mothers prefer their biological pups from this test.<br><br>**Experiment III**<br>- The latency of entries into tubes was significantly shorter on the biological pup side compared to the alien pup side.<br>- Mothers search for their own pups faster than they search for alien pups at the beginning of the test.

			and pup areas containing empty bottles by freely exploring for 10 min. Thereafter, the mother was shut in the neutral area by closing the mesh door; a biological pup and an alien pup, both male and postnatal day 5–6, were introduced into separate bottles. To enable the mother to smell the odour of these pups, airflow was produced<br>by the fan for 10 min. The test began once the fan was stopped and the mesh doors opened.<br><br>Exp. III<br>Two-choice tests were conducted in which mothers selected two tubes placed in opposite directions.<br>The subject mother and her pups of postnatal day 5 were moved into the testing apparatus from the breeding cage 24h before the tests, and recordings of pup USVs from both biological pups and alien pups of postnatal day 5 were conducted (see above). In the centre of the apparatus, nesting		- Latency of pup retrieval<br>- Duration of mesh search	**<u>Experiment IV</u>**<br>- There was no interaction between pup group and pup order on retrieval.<br>- The duration of mesh search and tube stay were longer and the number of entries into tubes was higher on the bio- logical pup USVs side, compared to those on the alien pup USVs side.<br>- Mouse mothers prefer the pup USVs of their biological pups over those emitted by alien pups.

			materials were placed inside a square acrylic frame to allow the mother to build a nest. All pups were removed from the apparatus 20 min before the pup-choice test. Immediately before the test, a mesh divider was inserted into each tube, and the pup-choice test began by removing the mesh of the external end and placing three biological pups and three alien pups (sexes were randomly mixed in each group) into separate tubes.<br>Then, a retrieving test was begun and the subject's response of retrieving pups into the nest from tubes was recorded for 30 min. After the retrieving test, alien pups were removed from the test cage, and the remaining mother and her biological pups were housed in the apparatus for another 24-h period until the reproduction of pup USVs. The nc-Si emitter was attached directly outside the mesh onto the end of			

			each tube 30 min before starting the pup USVs choice test, and biological pups were again removed from the apparatus. The recorded pup USVs from biological and alien pups were played back simultaneously from the separate speakers for 5 min.<br><br>Exp. IV<br>the selective-retrieving test was conducted in the breeding cage.<br>Two biological and two alien pups, of postnatal day 5 and with a mix of both sexes in both groups, were separately placed in the corner of the cage at an equal distance from the nest.<br>After the test, the subject and all her biological pups were moved into the testing apparatus as in Experiment 3, and allowed to acclimate for 24 h. Then, all pups were removed from the apparatus 24 h before the pup USVs playback, and the recordings of pup USVs were conducted			

			simultaneously. The nc-Si emitters were attached 30 min before the playback and the experiment was conducted following the procedure of Experiment III.			
**Author**	**Strain, Age, Number**	**Detection Method**	**Testing Condition**	**Housing conditions**	**Variables measured**	**Major findings**
(Noirot, 1964)	200 white female 1-2 months old mice from an outbred strain	NA	An experimental period began when the experimenter placed a young mouse about 10 cm. from the nest. He observed the adult female during the 5 min. following its contact with the baby. It was considered that contact had occurred when female's snout touched the baby.	Standard cages were made entirely of "plastic Plexiglas," 35 X 20 X 20 cm. in size. The femaleshad an unlimited diet of food pellets and drinking water, supplemented with some grain and vegetables; milk was given every 2 days. Cages were provided with nest material in the form of wood shavings.	Maternal behaviour:<br>- Retrieving: Female picked up the infant and brought or tried to bringing it into the nest,<br>- Licking: Female licked the infant for at least 20 consecutive sec.<br>- Nest building: for at least 20 consecutive sec. Female brought material into the nest, nibbled the material, and/or assembled (pushed with the snout or	- Retrieving and nest building, a sudden fall was observed atthe thirteenth day, while licking and lactation position seemed to decrease more gradually with increasing age of the young. The decrement seemed also to be less marked for licking than for the three other activities.

					scratched) the material,<br>- Lactation position: female covered the infant with its body and did not show any other activity for at least 20 consecutive sec.	
**Author**	**Strain, Age, Number**	**Detection Method**	**Testing Condition**	**Housing conditions**	**Variables measured**	**Major findings**
(Noirot, 1966)	Outbred albino mice<br><br>1-20 days old<br><br>15 litters	Capacitance microphone and Marconi 'B 29' radio receiver.<br><br>Isolation test: The receiver was tuned to a different frequency either 60, 65, 70, 75 or 80 k-cycles/sec.<br><br>Pup Retrieval test: all sounds during the test were recorded. The receiver was tuned at 70 k-cycles/sec	1) Individual isolation in an empty cage (5min): D1,2,3,4,7,9,10,12,13 ,16,18 and 18 days old; For each follow-up or independent observation 5 pups were chosen at random from the litter.<br>2) Pup Retrieval test: D1-10 for litter 1 and from D1-8 for litter 2. Pups were placed 10 cm from the nest.	No information provided	Calling rate<br>Frequency	Large increase on call detection on day 4, coincident with the opening of the ears, and a decrease, almost to zero, on the day during which the opening of the eyes was recorded.<br>More calls detected while ears were closed (D1-3) than while ears were opened (D4-12).<br>More calls detected before eyes opened (D1-12/13) than after (D12/13-20).<br>Number of calls detected during retrieving was higher than in isolation.<br>Calling rates were higher during the first minutes of isolation.<br>In follow-up litters, the rate of calling tended to be higher during the first than during the later minutes of isolation.<br>Proportion of detections was higher at the intermediate frequency ranges (65+70+75 k-cycles/sec) than at 60 and 80 k-cycles/se and varied between days.<br>The 60 k-cycles/sec curve seems bimodal in all three conditions, reaching peaks with the oldest and youngest pups.<br>The 70 k-cycles/sec curve shows different fluctuations with the different litters.<br>The 80 k-cycles/sec curve tends to decrease on successive days in all litters.<br>The percentage of clicks showed day-to-day fluctuations, decreasing from D1-4.

Supplementary Table 1 – Literature reviewed

Author	Strain, Age, Number	Detection Method	Testing Condition	Housing conditions	Variables measured	Major findings
(Noirot, 1966)	Outbred albino mice<br><br>1-12 days old<br><br>6+12 litters	Solid dielectric capacitance microphone, a broad-band amplifier and a Precision Instruments PS207 tape-recorder running at a tape-speed of 152 .4 cm per sec	Whole litter was taken out of the nest and put in a metallic dish	No information provided.	Frequency<br>Sound pressure Level	Frequency analysis:<br>Duration varies from about 10 msec to about 140 msec, but is most commonly 40 to 100 msec;<br>Length, initial and maximum frequency and total band- width changed greatly with age in all the litters.<br>Calls from one-day old pups start at a relatively high sound-frequency which then decreases, slightly at first and abruptly at the very end of the call.<br>D4 – all sonograms are more flat and present less variation.<br><br>Frequency variation range 50-90kHz<br><br>Sound Pressure Level Analysis:<br>Little variation between individual pups of the same age. Higher level pulses when handled.<br>Decrease with increasing age<br>D7 – drop of 10dB<br><br>**Length, total bandwidth and sound pressure decrease with successive days of age**
**Author**	**Strain, Age, Number**	**Detection Method**	**Testing Condition**	**Housing conditions**	**Variables measured**	**Major findings**
(Okabe et al., 2010)	ICR<br>33 males<br>36 females	USVs used in the experiments were previously recorded by Uematsu et al., 2007.	Some mice of both sexes, housed 3–5 individuals per cage, with no experience in mating and pup caring, were used as "virgin intact mice" (intact male, n = 9; intact female, n = 9), and other virgin mice were used in other groups. To produce gonadectomized mice, virgin mice of both sexes	Animals of each sex were housed in groups of 3–5 individuals per cage under a standard 12 h light/dark cycle (lights on from 6 a.m. to 6 p.m.). The environment was	Retrieving test<br>- Retrieve all pups<br>- Retrieve part of the pups<br>- Attack pups<br>- Ignore<br><br>Responsiveness to pup USVs:	**<u>Retrieving behaviour of females</u>**<br>- All mothers and 50–60% of ovx and sexually expe- rienced females retrieved pups, while less than 20% of intact and sham females retrieved pups.<br>- Attacking behavior was not observed in any group.<br>- The retrieving score was signif- icantly higher in mothers than in other females.<br><br>**<u>Retrieving behavior of males</u>**<br>- All cast and 50–60% of sexually experienced males and fathers retrieved pups, while less than 20% of intact and sham males retrieved pups.<br>- Attacking behavior was not observed in any group.

			were anesthetized with ether and either castrated (cast) or ovariectomized (ovx) at 7–16 weeks of age (cast, n = 7; ovx, n = 8). The controls underwent a sham operation (sham male, n = 9; sham female, n = 11). After surgery, the mice were again housed 3–5 individuals per cage and used in experiments two weeks later. To produce sexually experienced male mice, virgin male mice were paired with 2–3 virgin female mice, and the presence of a copulatory plug was checked every morning. After confirming the presence of the copulatory plug, the male mice were housed individually in cages for two weeks (sexually male, n = 9). To produce sexually experienced female mice, virgin female mice were anesthetized with ether and subjected to tubal ligation at 7–16 weeks of age to prevent pregnancy, following which they were housed with the virgin males.	maintained at a constant temperature (24 ± 1°C) and humidity (45 ± 5%). Food and water were provided ad libitum.	- duration of time during which a subject stayed in each tube and searched the mesh,<br>- the number of times the subject entered a tube	- The retrieving score was significantly higher in cast males than in intact and sham males.<br><br>**<u>Responsiveness of females to reproduced pup USVs in females</u>**<br>- The mean number of times all females entered a tube was significantly higher on the speaker side than on the non- speaker side.<br>- The mean durations of tube stay and mesh search were significantly longer on the speaker side than on the non-speaker side in ovx, sexually experienced females, and mothers.<br>- The mean durations of both tube stay and of mesh search of sexually experienced females were significantly longer than those of intact, ovx, and sham females as well as those of mothers.<br><br>**<u>Responsiveness of males to reproduced pup USVs</u>**<br>- The mean number of males entering a tube was significantly higher on the speaker side than on the non-speaker side in cast, sexually experienced males, and fathers.<br>- The durations of both tube stay and mesh search were significantly longer on the speaker side than on the non-speaker side in cast, sexually experienced males, and fathers.<br>- The number of times fathers entered a tube as well as their durations of tube stay and mesh search were significantly higher and longer than those of intact and sham males.

			After confirming the presence of a copulatory plug, the female mice were housed individually in cages for two weeks (sexually female, n = 11). The group of male parent mice comprised virgin male mice that had mated with virgin female mice, and had been allowed to care for their own litter for 6–8 days (father, n = 8). To produce female parent mice, virgin female mice were paired with virgin male mice only for two weeks before partu-rition and allowed to care for their own litter for 6–8 days after par-turition (mother, n = 10).<br><br>Retrieving test<br>The test cage was identical to the breeding cage, but it was divided by partitions into four compartments (height, 30 mm) to prevent pups from moving to the next compartment on their own.<br>6-day-old pups with no relation to the subjects were placed in the three remaining compartments (i.e., all			

			compartments except the compartment containing the nest), and the subject's behaviour was observed for 30 min.<br><br>Responsiveness to pup USVs<br>Recorded pup USVs were played back repeatedly using the nc-Si emitter for a 5-min testing period, and the subject's behaviour was observed during this period.			
**Author**	**Strain, Age, Number**	**Detection Method**	**Testing Condition**	**Housing conditions**	**Variables measured**	**Major findings**
(Okabe et al., 2013)	B6 mice<br><br>Two-choice test<br>N= 56 females<br><br>Neural responsiveness<br>N=38 females	Ultrasonic sounds were detected using a condenser microphone (Type 7016, Aco, Tokyo, Japan) designed for SPL measurements between 20 Hz and 90,000 Hz. The microphone was connected to an amplifier (UMA-2, Muromachi Kikai) and acoustic signals were trans-	<u>Preparation of Pup Odour</u><br>6 day old B6 pups were placed together in a glass beaker for 3h under a light bulb.<br><br><u>Ultrasound recording</u><br>Ultrasonic sounds were recorded when a 6-day-old B6 pup was isolated from its mother and littermates. The pup was not related to the subjects used in the study.<br><br><u>Ultrasound Reproduction</u><br>A nc-Si emitter was used.	Animals of each sex were housed in groups of 3–5 individuals per cage under a standard 12 h light/dark cycle (lights on from 6 a.m. to 6 p.m.). The environment was maintained at a constant temperature (24 ± 1°C) and humidity (45 ± 5%). Food and water were	Behavioural measures:<br>- Duration of mesh search<br><br>Immunohistochemistry:<br>- Number of c-fos positive cells in each section, and the mean number of cells in each nucleus	<u>**Two-choice tests**</u><br>- Mean mesh search durations were significantly longer for tubes where HP was presented compared to the tubes where AP was presented.<br>- There were no significant differences in the mean durations of the mesh searches between the tubes in which the following were presented: USVs only versus NS Odor only versus NS, and Cotton only versus NS.<br>- The simultaneous presentation of USVs and Odor made the mothers search the mesh for a significantly longer time compared to that with the presentation of Odor only.<br>- The responses by the mothers disappeared when USVs were exchanged for dUSs.<br><br><u>**Neural responsiveness to various stimuli**</u><br>- There was a significant effect of stimuli on the number of c-fos-positive neurons in several of the nuclei examined.

		mitted to a vocalization analyzer system (MK-1500, Muroma- chi Kikai) with functions such as an analog-to-digital converter (192 kHz), frequency filters, a digital fast-Fourier-transform analyzer, and signal input–output terminals. Input signals were visualized on SpectraLAB (Sound Technology Inc., U.S.A.) in the analyzer system.	The output from the nc-Sci emitter was monitored with a condenser microphone.<br><br>Apparatus Used for the Two-Choice Test<br>A plexiglass test cage of the same size as the breeding cage was used. A hole (4 cm in diameter, the centre of which was 6 cm from the left corner and 3.5 cm from the bottom) and 12 slits (8 x 12 mm, 4 mm between slits, 2 cm from the right corner and 1 cm from the bottom) were cut in each long wall to reduce sound echo. Tubes (40 mm outer diameter, 150 mm long, covered with mesh on the external end) were attached to the holes of the test cage.<br><br>Protocol for the Two-Choice Test<br>The subject mouse was exposed to a combination of stimuli for 5min:<br>• 6-day-old hypothermic pups (HP) versus 6-day-old	provided ad libitum.		- Compared to NS, the numbers of c-fos-positive cells following exposure to HP were significantly higher in those brain areas.<br>- However, compared to NS, the number of c-fos- positive cells that was observed following the presentation of USVs was only significantly higher in Au, and there were no significant differences in the other brain areas.<br>- compared to NS, the number of c-fos-positive cells following Odor presentation was only significantly higher in MoB.<br>- Compared to NS, the numbers of c-fos-positive cells following the simultaneous presentations of USVs and Odor were significantly higher in Au, MoB, BLA, CeA, BNST, and MPOA.<br>- In the BNST, the numbers of c-fos-positive cells that were observed following the simultaneous presentations of USVs and Odor were significantly lower than the numbers found following HP.<br>- There were no significant differences in the numbers of c-fos-positive neurons in MeA, BMA, or PFC among the study groups.

			anesthetized pups (AP),<br>• reproduced pup USVs (USVs) alone versus no stimuli (NS),<br>• pup odor cotton (Odor) alone versus NS,<br>• USVs with Odor versus Odor alone,<br>• dUSs with Odor versus Odor alone,<br>• cotton alone (Cotton) versus NS.<br><br><u>Test Protocol for Investigating Neural Responsiveness to Various Stimuli</u><br>A subject and its own pups, which had not been used for behavioral testing, were moved to a test cage.<br>After it was confirmed that the nest was built, all pups were removed from the test cage. After the subject was further allowed to habituate for 24 h, a partition was inserted around the nest in order to prevent the mother from leaving this enclosure.			

			A stimulus was presented immediately outside the mesh on a hole near the nest for 5 min. The numbers of stimulus exposures were as follows: HP; USVs with Odour; USVs alone; Odour alone; and NS.<br><br><u>Immunohistochemistry</u><br>Subjects were perfused and the brains were collected.<br>In each subject, the location of neuroanatomical areas, including In the BNST, the numbers of c-fos-positive cells that were observed following the simultaneous presentations of USVs and Odor were significantly lower than the numbers found following HP. There were no significant differences in the numbers of c-fos-positive neurons in MeA, BMA, or PFC among the study groups., were determined with a brain atlas. In each nucleus, a fixed number of sections, which were anatomically matched among subjects, were analysed.			

Supplementary Table 1 – Literature reviewed

**Author**	**Strain, Age, Number**	**Detection Method**	**Testing Condition**	**Housing conditions**	**Variables measured**	**Major findings**
(Okon, 1970a)	Outbred albino mice<br><br>1-20 days old<br><br>4 litters (2+2)	Bruek and Kjoer type 4135 ¼ inch microphone, a type 2604 microphone amplifier and an oscilloscope. The amplifier was connected to a filter which attenuated frequencies below 10kHz. The microphone was inserted into the observation chamber through a hole in the Perspex front wall and was supported in a stand 10 cm above the floor of the chamber.	Exposure to different environmental temperatures :<br>- 33, 22, 12ºC<br>- Above 33ºC and below 2-12ºC<br><br>Pups were placed in a shallow plastic dish 12 cm in diameter and 3,5cm deep inside a constant temperature cabinet (modified refrigerator).	No information provided	Sound pressure and call rate<br>Body temperature	Almost no USVs produced on D1-2 at the different temperatures.<br>Loud pulses produced when pups were handled or fell over and rolled on their backs even on D1.<br>Increase in the intensity D6-7 with peak occurring between D8 and D12-13<br>At 22ºC, calling ceased at D14<br>12ºC – calling ceased at D16<br>2-3ºC – Calling ceased on D18-19<br><br>None of the pups produced any call after D20.<br><br>33ºC – minimal emission. Call ceased on D9-11
**Author**	**Strain, Age, Number**	**Detection Method**	**Testing Condition**	**Housing conditions**	**Variables measured**	**Major findings**
(Okon, 1970b)	Outbred albino mice<br><br>Age – D2-D16	Bruek and Kjoer type 4135 ¼ inch microphone, a type 2604 microphone	Handling experiment: Individual isolation in a shallow plastic dish. 3 different handling methods used – **holding and lifting** pups between two fingers (normal	No information provided.	Pulse intensity (Sound pressure)<br>Frequency (dam)	Normal handling: Loudest pulses during the first 4 days, ceased until D14-15.<br>Falling – Similar pattern. Higher intensity that decreased ~D5-6. Ceased at D8.<br>Retrieving – More gradual decline from D6 and ceased on D12<br>Tapping – Loud pulses produced until D8 and ceased D16.<br>Accompanied by audible squeaks (not produced simultaneously).

	6 litters (4+2)	amplifier and an oscilloscope. The amplifier was connected to a filter which attenuated frequencies below 10kHz. Microphone was placed in a stand 10 cm above the dish	handling), **tapping or pressing the tail** against dish surface, **pinching scruff** of the neck, **falling** by tipping the dish and retrieving by the dam.			Production of USVs by the mother: Erratic. Often soon after the last pup was removed.<br>Some detected between D1-15 after parturition. Low intensity and no changes were detected between days. The dam produced more pulses if presented with the babies and then deprived of them again. 60-80kHz 40-50mseconds. Freq pattern very similar to those of infants.

Author	Strain, Age, Number	Detection Method	Testing Condition	Housing conditions	Variables measured	Major findings
(Robinson and D'Udine, 1982)	BALB/c<br>SEC<br>C57<br>0 – 13 days old<br><br>20 BALB/c pups<br>20 SEC pups<br>24 C57 pups	Routine observations were made with an ultrasound detector constructed by one of the authors using a transducer from a navigation aid for the blind. This detector has a frequency range of30-110 kHz. The output from an external oscillator is mixed with the incoming signal and the difference signal is amplified before being fed to an	The first pup was taken from the litter and placed in a small cage (8 x 8x 8 cm) on a heated mat in front of an ultrasound detector. The number of calls produced was recorded over a 5-min period. The period started at the end of any calls produced as the pup was placed on the mat, since these calls would have been produced in response to handling. At the end of the 5-min period a second pup was removed from the litter and placed on the mat while the first pup was replaced in the litter. During the observations the heated mat maintained a	Animals were maintained on a 12 h light/12 h dark regime with the light period starting at 20.00 h G.M.T. The room temperature was zooc. All litters were culled to four at birth (Day 0) and the male parents were removed either at the same time, or before the birth if possible.	- Number of calls	- The level of ultrasound activity is different in the different strains. However, the period over which ultrasound is produced is markedly different in C57, peaking between Days 2 and 4. In BALB/c activity peaks between Days 3 and 7, and in SEC between Days 4 and 8.<br>- On some days, particularly shortly after birth and towards the end of the 14-day period, some pups produced no calls during the 5-min test period.<br>- A comparison of the mean number of calls for each strain showed that the BALB/c strain differed significantly from SEC, and that SEC differed significantly from C57. Consequently, C57 and BALB/c differed significantly.<br>- All three strains use a fundamental frequency of 60-70kHz predominantly.

		earpiece via an audio-amplifier. The effective bandwidth is ±15 kHz of the centre frequency. The ultrasound detector used was set to a centre frequency of 65 kHz, giving a bandwidth of 50-80 kHz by adjusting the external oscillator to 65 kHz using the frequency counter (Fig. I). A QMC Instruments Mini Bat Detector was used to check the frequency of ultrasound from the litters. This instrument is a tuned superheterodyne type (Sales & Pye, 1974) which gives an audible response to calls within 5kHz of the	temperature of 34-38°C at the surface on which the pup lay, so the effect of isolation could be measured independently from the effect of the temperature drop usually associated with removal from the litter.			

		frequency to which it is tuned.				
**Author**	**Strain, Age, Number**	**Detection Method**	**Testing Condition**	**Housing conditions**	**Variables measured**	**Major findings**
(Santucci et al., 1994)	Pups:<br>CS1 mice<br>2 – 10 days old<br>13 litters with 4 males and 4 females each<br><br>Adult males:<br>CS1 mice<br>N=26<br>3-4 months old	A QMC microphone was suspended 5 cm above the dish and connected to a S-25 Bat Detector (Ultrasound Advice, London, UK) tuned to 70 kHz. The number of ultrasonic calls was monitored using the audible output of the detector and recorded on a hand-held counter for a 5 min period during one of two sequences of odour presentation.	<u>Categorization of adult males (infanticidal vs non-infanticidal)</u><br>Single newborn pup was placed in the centre of the cage, and the behavioural response of the adult was evaluated for 10 min. If the pup was harmed, experimenter intervention ensured that it was humanely killed within 10 s of an attack, and the adult male was assigned to the 'infanticidal' group. The male was classed as 'non-infanticidal' if it either ignored the pup or showed parental behaviour towards it.<br><br><u>Urine collection</u><br>Urine was provided by 26 adult males, 13 infanticidal and 13 non-infanticidal. Donor males were held at the back of the neck and tail, a procedure that normally causes urination in mice, and sufficient urine was	Mice were housed in single-sex stock cages(40 × 24 × 12 cm) containing 8-12 mice in each cage. Food (Morton's rat, mouse, monkey mix) and water were provided ad libitum; wood shavings were used as cage litter. The experimental room was maintained at 19-22°C and lighting was on a 12 : 12 h light:dark regime, all observations being made in the light period.	- Number of ultrasonic vocalizations/min	- For minute 1 (no odour) there was a clear age effect on the number of vocalizations, a trend for females to vocalize more than males, but no effect of the type of urine to be offered in the next minute.<br>- For minute 2, when the urine odours were present, there was an age effect, a sex effect with more calling by females, and a urine effect with significantly more calls produced in the presence of urine from infanticidal males than non-infanticidal males.<br>- For minute 3 (no odour) there was an age effect but no sex effect and no effect due to the type of urine present in the preceding minute.<br>- For minute 4 there was an age effect, a sex effect with more calling by females, and a urine effect, again with more calls when infanticidal male urine was present.<br>- On minute 5 (no odour) there was an age effect, a trend towards a sex effect, but no effect of the type of urine present in the preceding minute. There were no significant interaction effects on any of the analyses above.<br>- Pups exposed to urine of infanticidal males on minute 2 and then to urine of non-infanticidal males on minute 4 generally showed a substantial decline in the rate of calling, whereas those exposed to urine in the reverse order generally showed a slight increase.<br>- Positive correlations were found between the number of vocalizations and the body weight of the pups on day 2 and 6, and a negative correlation was found on day 10. However, there were no significant differences in the body weight of males and females.

			collected to wet the end of a 'cotton-bud'.<br><br><u>Odour presentation</u><br>Two sequences of odour presentation were performed.<br>In sequence A, the odour of urine from an infanticidal male was presented during the second minute and odour of urine from a non-infanticidal male was presented during the fourth minute. In sequence B, the order of odour presentation during minutes 2 and 4 was reversed. No odour was presented in the first, third or fifth minutes. The experimental sequence was different for the two pups of each sex in each litter.			
**Author**	**Strain, Age, Number**	**Detection Method**	**Testing Condition**	**Housing conditions**	**Variables measured**	**Major findings**
(Smotherman et al., 1974)	14 female C57BL/10 75-88 days of age	Vocalizations were recorded on an Ampex (PR 500) instrumentation recorder. To record the ultrasounds, a 6-day-old pup isolated at 24°C	The testing chamber was constructed of heavy cardboard; the home cage served as the start box; the choice arms were made of Plexiglas painted black with grid floors. A Masonite ledge prevented the target animals from being	The pregnant females were housed in cages that measured (24.1 × 13.9 X 7.6 cm). The home cages were equipped with	- Choice of stimuli	- The lactating females chose the handled pup (olfactory/auditory cues) when it was pitted against the chilled pup (olfactory cue alone).<br>- The handled pup (olfactory/ auditory cues) was preferred over the tape loop (auditory cue alone) in the condition where they opposed each other.<br>- In the chilled pup (olfactory cue alone) vs tape loop (auditory cue alone) condition the females entered the arm that held the chilled pup (olfactory cue alone) on significantly more trials.

Author	Strain, Age, Number	Detection Method	Testing Condition	Housing conditions	Variables measured	Major findings
		was recorded in an Industrial Acoustics, Inc. (Model 1200) sound chamber. A tape loop of these vocalizations was recorded on 1-mil Mylar instrumentation tape and played back on an Ionovac (Model DVK5) ultrasonic speaker. Signal characteristics were analysed on a Kay Sonagraph (Model 6061 B).	visible and wire mesh restrained the females from retrieving the target animals.<br><br>Neonatal ultrasounds were recorded from a 3-day-old C57BL/10 pup isolated at a temperature of 24°C.<br><br>Testing took place on days 3-7 postparturition.<br><br>5 experimental conditions:<br>- handled pup vs chilled pup,<br>- handled pup vs tape loop,<br>- chilled pup vs tape loop,<br>- tape loop vs empty arm, and<br>- chilled pup vs empty arm.	tops that were removed when the cage was placed in the testing chamber.		- No differential choice behavior was shown in the tape loop (auditory cue alone) vs empty arm (no cue) condition.<br>- The chilled pup (olfactory cue alone) elicited more responses than the empty maze arm (no cue) in the condition where they were paired.
**Author**	**Strain, Age, Number**	**Detection Method**	**Testing Condition**	**Housing conditions**	**Variables measured**	**Major findings**
(Thornton et al., 2005)	4 x 4 diallel or complete intercrossing of the C57BL/10J, DBA/2J, BALB/cJ and SJL/J strains. 16 genetic groups:	Vocalizations were recorded with a Bruel and Kjaer 0.635 cm microphone placed about 5cm above the cup, a B-K 2619T preamplifier, and a Precision	Longitudinal testing from 2 to 12 days of age.<br><br>2 experimental conditions:<br>1) Cold plus isolation (as described by Okon,1970a): Pups aged 2-7days. A single	The cages were standard, transparent, colony cages (30 x 20 x 15 cm) with stainless steel tops. Breeding pairs were	Frequency<br>Call length<br>Rate of calling	• SJL/J strain had a lower rate of calling and higher ending frequency than the other parental strains.<br>• BALB/cJ tended to have longer calls and greater range in frequency of calls than the other inbred strains.<br>• Rate of calling: Additive variance accounted for approximately 18% of the total variance for calling rate. However, its influence decreased as the animals aged so that by 9 days of age, the effects accounted for approximately 7% of the variance. Directional dominance was also significant for rate of calling from days 3–7, and contributed from 4% on day 7 to 11% of the variance on

	- 4 inbred strains<br>- 6 F1 hybrids<br>- 6 reciprocal F1 hybrids<br><br>n=486<br><br>Litters from 4-8 pups<br><br>about equal numbers of males and females<br><br>2-12 days of age	Data PI 6204 instrumentation tape recorder. A frequency response of 150 Hz to 150 kHz was obtained using Maxell UDXL 50–60 recording tapes and a recording speed of 0.938 m/s. The ultrasound characteristics listed above were measured using a Kay Elemetrics 5500 Digital Sonagraph sound spectrum analyzer. The tapes were played back at 1/10 of the recording speed (93.8 mm/s).	pup was randomly chosen and placed on a cotton pad inside an aluminum-weighing dish placed on 100mL of ice in a 250mL beaker. The surface temperature of the cotton pad was maintained at 10-11C. The beaker was placed in the dark, sound attenuated chamber with the air temperature at 21C. Recording began immediately and continued for 18 seconds.<br>“Falling” procedure: Pups aged between 8 and 12 days, The pup was placed in a 250mL beaker held at 45°C and the cup was rotated by hand at a rate of half a revolution per second. As the cup rotated, the pup experienced short	allowed food (Agway RMH 3000 animal chow) and water ad libitum. Lighting was maintained on a 12 hour light/12 hour dark cycle with lights on at 0800h. The colony and testing rooms were maintained at 21 ± 1 C.		days 4 and 5. The F_1 values drop closer to the parental means as the animals age, demonstrating the decreasing role of dominance in this behavior. Maternal effects were present on days 4, 5, and 6 but comprised less than 5% of the phenotypic variance. However, maternal influences accounted for 12% of the variance on day 8.<br>• Length of calls: Additive variance was significant for all 7 days for length of calls. Its contribution to the total variance was estimated to be 34% at 3 days of age, and, similar to rate of calling, its influence decreased as the animals age. Directional dominance also contributed significantly to length of calls on days 3–8 and asymmetrical dominance was significant at days 3, 4, 5, and 9. Maternal effects were notably absent, except at day 9, where they accounted for 6% of the variance. Other reciprocal effects were also present on several days, but contributed relatively little to the phenotypic variance.<br>• Beginning and ending frequency: Additive genetic variance and directional dominance made significant contributions to both call characteristics. In fact, the genetic structure appeared to be similar for the two traits, with additive genetic variance contributing significantly to all ages, directional dominance accounting for a substantial proportion of the of variance for ages 3–8. In addition, asymmetrical dominance was present on days 5–7 and specific combining effects were present on day 9 for both beginning and ending frequency.<br>• High frequency: Additive genetic variance accounted for 16–22% of the total variance for high frequency on most days. Dominance effects were also substantial for days 3–6. Maternal effects, while present, accounted for only a small proportion of the variance. Day 7 was notably different from the other days as only 7% of the variance was accounted for by the sources we examined, indicating consistent measurement error.<br>• Low frequency: Additive and dominance sources contributed significantly to all days of measurement and accounted for up to 61% (day 5) of the observed variance.

			(34cm) falls inside the cup. Recording as described on 1).			Additive effects decreased in importance with age. Dominance effects showed a marked decrease on days 8 and 9 as compared to the other ages measured. Maternal sources of variance were significant on days 8 and 9.<br>• Range in frequency: In contrast to the other measures, maternal effects were significant sources of variance across most ages, with day 7 being the exception. Similar to the other measures, additive and dominance effects made the greatest contributions to the variance, except at day 9.
**Author**	**Strain, Age, Number**	**Detection Method**	**Testing Condition**	**Housing conditions**	**Variables measured**	**Major findings**
(Uematsu et al., 2007)	ICR<br>7 weeks old<br><br>7 day old pups<br><br>Females<br>N=8	Ultrasonic sounds were detected using a condenser microphone (Type 7016, Aco, Tokyo, Japan) designed for SPL measurements between 20 Hz and 90,000 Hz. The microphone was connected to an amplifier (UMA-2, Muromachi Kikai) and acoustic signals were trans- mitted to a vocalization analyzer system (MK-1500, Muroma- chi Kikai) with	Experimental apparatus<br>The test cage consisted of a clear Plexiglas cage (175×245×125 mm), the same size as a breeding cage. A hole and 12 slits were cut in the each long wall to reduce echo sounds. Tubes were mesh- covered at one end and were attached to the holes of the testing apparatus. Partitions could be inserted into the tube 1 cm from the cage.<br><br>Pup USVs recording<br>Individual 7 day old pups were placed in a glass beaker in the chamber.<br><br>Maternal response to pup USVs	Animals were housed at 3–5 per cage in a room with a 12:12 h light/dark cycle (lights on 8 a.m. to 8 p.m.), with the environment maintained at a constant temperature (24± 1 °C) and humidity (45 ± 5%). Food and water were provided ad libitum.	Behavioural measures:<br>- Duration which a mother stayed in each tube<br>- Duration of mesh search<br>- Number of times of tube entry<br>- Latency to enter a tube<br><br>USVS:<br>- Calling rate<br>- Frequency (kHz)<br>- Voltage	**Maternal response to pup USVs**<br>- The mean durations of stay in a tube and of search the mesh were significantly longer on the hypothermic pup side than on the anesthetized pup side.<br>- The mean number of times the mother entered a tube was significantly higher on the hypothermic pup side than on the anesthetized pup side.<br>- No significant difference in the latency to enter a tube<br>- The mean calling rate of pup USVs was 558.5±78.8 USVs over 5min.<br><br>**Maternal response to reproduced ultrasounds**<br>- When pup USVs were reproduced, the mean durations of stay in a tube and of search the mesh were significantly longer on the speaker side than on the non-speaker side.<br>- The mean number of times a mother entered a tube was significantly higher on the speaker side than on the non-speaker side.<br>- The latency to enter a tube was significantly shorter on the speaker side than on the non- speaker side.<br>- When synthesized double-duration ultrasounds, silence domain-double ultrasounds, or ultrasounds domain-double ultrasounds were used, there were no differences in the duration of stay in a tube or search the mesh, the number of times a mother entered a tube or the latency to enter a tube.

		functions such as an analog-to-digital converter (192 kHz), frequency filters, a digital fast-Fourier-transform analyzer, and signal input–output terminals. Input signals were visualized on SpectraLAB (Sound Technology Inc., U.S.A.) in the analyzer system.<br><br>For pup USV recording, the microphone was placed 50 mm from the bottom of the beaker and aligned with its centre. The microphone noise floor was around 28 dB. High-pass filter processing was used with a corner frequency of 20 kHz. The	Experiments were performed on postnatal day 6-8.<br>On the experimental day, tubes were attached to the apparatus and it was placed in a soundproof chamber.<br>After the habituation period, the mother was removed from the testing apparatus and placed in a breeding cage in an experimental room while all pups were moved to a different room out of auditory and olfactory range of the mother and kept warm under a light for 15 min. Then a partition was inserted into each tube, and the mother was brought back to the testing apparatus. Five minutes later, two pups were exposed to the mother simultaneously by placing each one outside a mesh covered tube. One male pup was placed in a Plexiglas vessel (5×8×5 cm) that was maintained at 14 °C on an ice bag. A second male pup was anesthetized. After		- Sound pressure level	

		sampling frequency rate was 192 kHz	confirming that the hypothermic pup emitted USVs, the partitions were removed and the behaviour of the mother was observed for 5 min.<br><br><u>Maternal response to reproduced pup USVs and synthetic ultrasounds</u><br>After the mother was An nc-Si emitter was placed outside one of the mesh-covered tubes.<br>The stimuli were:<br>- Reproduced original pup USVs (n = 8),<br>- double-duration ultrasounds (n = 6),<br>- silence domain-double ultrasounds (n = 6), or ultrasounds domain-double ultrasounds (n = 5)<br>Three types of reproduced pup USVs were edited and were 10 s long and contained two whistles			
**Author**	**Strain, Age, Number**	**Detection Method**	**Testing Condition**	**Housing conditions**	**Variables measured**	**Major findings**

(Wohr et al., 2008)	C57BL/6NCrl (B6N)<br><br>C57BL/6JOla Hsd (B6JOla)<br><br>Exp I:<br>7 days old pups in four developmental conditions (Donor strain > Recipient strain): B6JOla > B6JOla (n: males = 17, females = 16; 6 litters), B6Jola > B6N (n: males = 7, females = 9; 4 litters), B6N > B6N (n: males = 27, females = 18; 7 litters), B6N>B6JOla (n: males = 12, females = 8; 3 litters).<br><br>Exp. II<br>10 B6N females	Ultrasonic vocalization was recorded using two UltraSoundGate Condenser Micro- phones (CM 16; Avisoft Bioacoustics) suspended 12.5 cm from the testing surface. They were connected via an Avisoft UltraSoundGate 416 USB Audio device (Avisoft Bioacoustics) to a personal computer, and were recorded with a sampling rate of 214,285 Hz in 16 bit format. Thereafter, ultrasonic vocalization was analyzed using Avisoft SASLab Pro	<u>Experiment I – embryo transfer</u><br>Four developmental conditions were created (Donor strain > Recipient strain):<br>- B6JOla > B6JOla (n: males = 17, females = 16; 6 l),<br>- B6Jola>B6N (n: males = 7, females = 9; 4 l),<br>- B6N>B6N (n: males = 27, females = 18; 7 l),<br>- B6N > B6JOla (n: males = 12, females = 8; 3 l).<br>Surgically embryo transfer was performed.<br><br>*Maternal Retrieval Behaviour*<br>On postnatal day 7, maternal retrieval behavior was induced by removing all pups of a given litter from the nest and placing them in the edge most distal from the nest.<br><br>*Isolation*	All mice were housed in Makrolon type II long cages (36 x 21 x 12 cm) in the specified pathogen free mouse facility of the Gene Centre in Munich. Water and food (Ssniff, Germany) were freely available. Room temperature was 25°C with 40% humidity and a 12-h light/12-h dark cycle (lights on at 7 am).	USVs:<br>- Peak frequency (kHz)<br>- Number of ultrasonic vocalizations<br>- Total calling time (s)<br>- Call duration (s)<br>- Frequency modulation (kHz)<br>- Peak amplitude (dB)<br><br>Behavioural parameters:<br>- Time in petri dish (s)<br>- Time in proximal area (s)	**<u>Experiment I – embryo transfer: ultrasonic vocalization</u>**<br>*Within strain embryo transfer*<br>- B6JOla pups emitted more calls than B6N pups irrespective of gender.<br>- Total calling time and call duration did not differ between sub-strains, whereas females generally spent more time calling than males.<br>- Female calls were longer than male calls.<br>- Calls emitted by B6JOla pups were higher in frequency and amplitude whereas gender had no effect.<br>- Frequency modulation was higher in females but did not differ between sub-strains.<br><br>*Between-strain embryo-transfer*<br>- The finding that B6JOla emitted more calls than B6N was based on early environmental factors whereas pup genotype did not directly contribute to the observed difference.<br>- Pups born and raised by females of the same sub-strain emitted higher rates of ultrasonic calls in comparison to pups born and raised by females of the other sub-strain. This was especially evident for B6JOla pups.<br>- Gender did not directly or indirectly influence call number.<br>- The genotype of the mother affected the time spent calling whereas the genotype of the pup did not directly affect total calling time.<br>- Pups born and raised by mothers of the same sub-strain spent a longer time calling than pups born and raised by the other sub-strain.<br>- Gender had no effect on total calling time,<br>- Call duration was independent from genetic background, early environmental factors and gender.<br>- Peak frequency was dependent on pup genotype only, since B6JOla pups emitted calls with a higher peak frequency than B6N irrespective of the genotype of the mother or pup gender.

	10 B6JOla females		Pups were isolated from the mother and nest on postnatal day 7.<br>Pups were individually removed from the nest in random order and gently placed into a dish (8 x 8 x 3 cm) on a warming plate at 27°C.<br><br><u>Experiment II – Maternal Search Behaviour</u><br>*Playback task*<br>Testing of maternal responses to playback of ultrasonic calling was performed on an elevated white platform when pups were 7-10 days old.<br>In the center of the platform, a petri dish was situated, which was filled with soiled bedding from the home cage, i.e. from the nest.<br>For playback, two ultrasonic speakers were used. They were placed opposite to each other and 20cm away from the platform.<br>One speaker was pseudo- randomly chosen for playback, i.e. counter-balanced for strain of the mother and test order.			- peak amplitude was fully dependent on maternal effects. Pups born and raised by B6JOla emitted calls with a higher peak amplitude than pups born and raised by B6N.<br>- Frequency modulation was not dependent on the genotype of the mother but on the genotype of the pup and its gender.<br>- Calls emitted by females were more modulated than those of males and calls emitted by B6N were more modulated than those of B6Jola.<br>- Call amplitude is solely dependent on maternal effects, whereas call frequency and frequency modulation are solely dependent on the pup, i.e. its genotype and gender.<br>**<u>Experiment I – embryo transfer: maternal retrieval behaviour</u>**<br>*Retrieval task*<br>- No evidence for a difference in retrieval behavior between B6N and B6JOla mothers was obtained.<br>- Pup genotype affected the latency to pick up the first pup, since B6JOla were picked up sooner than B6N.<br>- Pup genotype did not affect the actual latency to retrieve the first pup and no significant interactions were obtained for the latency to pick up or retrieve the first pup.<br><br>**<u>Experiment II – Maternal Search Behaviour</u>**<br>*Playback task*<br>- During the first playback of ultrasonic vocalizations, moth-ers spent more time in the petri dish than before and after playback, irrespective of strain whereas behavior was unchanged during playback of white noise and the second playback of ultrasonic vocalizations.<br><br>*Pup discrimination task*<br>- B6JOla mothers spent more time in contact with pups, which emitted calls with high peak amplitudes in comparison to pups, which emitted calls with low peak amplitudes.<br>- The preferences shown by B6N mothers were not related to peak amplitude.

			The following three acoustic stimuli were presented:<br>(1) white noise,<br>(2) B6JOla ultrasonic vocalizations, and<br>(3) B6N ultrasonic vocalizations.<br>All stimuli were presented with a sampling rate of 192 kHz in 16 bit format with 65 dB.<br>A given animal was placed into the petri dish with bedding from the nest. Behavioral recording started as soon as the mouse had left the Petri dish for the first time (all four paws on the platform). After an initial habituation phase (3 min), the mouse was exposed to 3 presentations of acoustic stimuli for 1 min, each followed by an inter-stimulus-interval of 3 min. The first stimulus presented was white noise. The second and third stimuli were ultrasonic vocalizations of the own strain, i.e. B6JOla mothers were exposed to B6JOla calls and B6N mothers to B6N calls.			- Call number did not differ between pups attracting the mother for a short or long time in either strain.

			Pup discrimination task<br>In the same set-up as the playback test, in the forward middle of the platform, a petri dish was situated, which was filled with soiled bedding from the home cage, i.e. from the nest. Additionally, two smaller petri dishes without bedding material were situated in the two most distal corners. In each of them, a stimulus pup from a foreign litter was placed. To avoid differences in age, stimulus pups were littermates.<br>A given mother was placed into the petri dish with home cage bedding. The experiment was started when the mother left the Petri dish for the first time (all four paws on the platform), and stopped when the first pup was removed from the small petri dish.			

Supplementary Table 1 – Literature reviewed

Parental cooperation

Author	Strain, Age, Number	Detection Method	Testing Condition	Housing conditions	Variables measured	Major findings
(Liu et al., 2013)	Male and females<br><br>Slc:ICR outbred mice<br><br>45-55 days of age	Recordings took place in a soundproof chamber.<br>**USVS**<br>The microphone was placed 50 cm above the cage in a soundproof chamber and connected to an amplifier (model UMA-2; Muromachi Kikai, Tokyo,	**Behavioural testing**<br>Virgin males and females were paired and continuously housed together from mating period until 3-5 after delivery of the pups.<br><br>In the first experiment, one parent was left in the original cage alone or with his or her mate for 10 min, and the removed sire or dam and all of the pups were placed in holding cages.	"The mice were housed together continuously in standard mouse maternity cages."	**USVs**<br>Number of calls<br>Frequency<br>Wave width<br><br>Parental behaviours:<br>- latency to retrieve the first pup<br>- total time required to retrieve five pups	**Maternal behaviour in home cages**<br>The sire cared for the pups in the nest in the absence of the dam or together with her. Paternal care involved mainly licking behaviour and huddling over or near the pups.<br>Paternal retrieval behaviours were very infrequent.<br><br>**Parental behaviour after parent-pup separation**<br>After separation, when dams were reunited with the pups, they rapidly gathered the pups and resumed nursing.<br>The sire assumed a similar parental behaviour irrespective of whether he remained in the home cage or had been removed with the dam.<br><br>**Paternal care after separation into new clean cages**<br>Female parental care was unchanged. Male parental care also remained unchanged when sires were housed with dams but was

		Japan). Acoustic signals were transmitted to a vocalization analyser system (model MK-1500; Muromachi Kikai) equipped with an analogue-to-digital converter (192 kHz), frequency filters, a digital fast-Fourier-transform analyser and signal input–output terminals. Input signals were visualized using a SpectraLAB (Sound Technology Inc., State College, PA, USA) analyser system on a personal computer (Fig. 5). USVs were also recorded with a microphone (SF-12DC) equipped with an amplifier (model	In the second experiment, the sire and/or dam was placed in a clean cage with new woodchip bedding, but the pups were left in the nest in the original cage. The parents remained in the test environment for 10 min or for 3–30 min. Five pups were then selected from the litter and placed individually at a site remote from the nest in the original cage. The parents were returned separately to the original home cage with the test pups to assess parental behaviour, Parental retrieval behaviour (latency to retrieve the first pup, total time required to retrieve five pups and percentage of sires or dams exhibiting retrieval) was examined for 10 min following the reunion.<br><br>In some of the experiments, the parent was kept individually in a separated cage. Parental males were rendered deaf or anosmic.<br><br>**<u>Tests of sociability</u>**		Tests of sociability:<br>- amount of time spent in each chamber<br>- the number of entries into each chamber<br>- the number of transitions between chambers of the apparatus.	significantly reduced when sires were housed separately from the dams.<br><br>**<u>Social interaction of paternal males in a three-chambered box</u>**<br>Sires that had cohabited with their mates for 3, 5 and 10 min showed a relatively constant preference among the three chambers. On the contrary, 20 sires isolated alone gradually exhibited a preference to the left (pups) side and, finally after 10 min, a significant preference for spending time in the empty chamber. The immobilized time was significantly increased in the left interacting zone with pups, but not in the right zone without pups, by sires isolated with mates for 10 min, while this tendency was not observed in sires isolated alone.<br><br>**<u>Cues for paternal care induction</u>**<br>When the dams were placed in a transparent plastic box with a lid that blocked transmission of auditory and olfactory stimulation, paternal care was significantly reduced, suggesting that paternal behaviour is evoked by exogenous stimuli.<br><br>**<u>USV measurements</u>**<br>USVs consisting of short bursts of a complex, upward, downward, harmonic frequency-modulated tone as previously were detected. The unique USVs emitted by the dams had a fundamental frequency between 30 and 40 kHz, a mean peak frequency of 38.7±0.5 and a mean duration of 121±26 ms. The average number was 5.1±0.8 calls per min during isolation with mates. 12/13 females vocalized. The characteristics of the 38-kHz USVs emitted by the pup-deprived dams were quite different from those of the USVs emitted by virgin female mice of the same age during female–female encounters, they occurred at a frequency of 81±7 calls per min and consisted of two main components of 47.8±5.2 and 68.4±4.9 kHz. Confirmation of USV emission by the female was performed by anaesthetizing the male and recording USV.<br><br>**<u>Induction of paternal behaviour by USV</u>**<br>Nine sires retrieved pups out of 15 subjects tested by playback of female's USVs. A replay of a 38-kHz sinusoidal (control) noise failed to initiate male parental care. Interestingly, as expected, playback of

		DAF1010; DiaMedical System, Tokyo, Japan).	The tests were performed in a rectangular three-chambered box. Each new sire that was separated from his pups for the first time was placed in the middle chamber alone or together with his mate and was allowed to explore for 3, 5 or 10 min. Five pups taken from a family cage were then placed in the left chamber and enclosed in a small round wire cage, which allowed nose contact between the bars. At the end of the first separation, each parental male was tested in a second 10-min session to quantify social preference for the empty chamber or the chamber containing the pups (in the cohabitation case, the mate was removed during this second test). The amount of time spent in each chamber, the number of entries into each chamber and the number of transitions between chambers of the apparatus were			virgin female-to-female USVs did not induce paternal behaviour in the sires.<br><br>**<u>Interaction of auditory and olfactory communication</u>**<br>This exposure to maternal pheromonal stimulation resulted in paternal behaviour in five sires retrieved out of nine. We then combined the olfactory stimulus with the pre-recorded USVs, which slightly increased male parental behaviour to 67%.<br>Deafness and anosmia did not inhibit subsequent male pup retrieval, but sires subjected to both procedures failed to retrieve their pups. These results suggest that the USVs and olfactory pheromonal stimuli emitted by females provide essentially equivalent and independent social signals that stimulate neural circuitry24,26 and induce parental behaviour in sires.

			determined during the second 10-min session.<br><br>**USV Playback**<br>USVs produced by females were recorded in a soundproof chamber. On the following day, the sire was placed in a new cage in the soundproof chamber for 10 min (1–4 calls per min). During this time, USVs recorded from its mate were played back; the sire was then tested for retrieval behaviour in the original home cage with five pups.			
**Author**	**Strain, Age, Number**	**Detection Method**	**Testing Condition**	**Housing conditions**	**Variables measured**	**Major findings**
(Liang et al., 2014)	Male and female Slc:ICR (Swiss CD1), C57BL/6 and BALB/c	The microphone (Type 7016; Aco, Tokyo, Japan) was placed 50 cm above the cage in a soundproof chamber and connected to an amplifier (model UMA-2; Muromachi Kikai). Acoustic signals were transmitted to a vocalization analyzer sys-	Experiments were carried out in a soundproof chamber measuring 600 x 500 x 500 mm (model MC-050/VA; Muromachi Kikai, Tokyo, Japan).<br><br>One male parent was placed for 10min in the original cage or new cage alone or with his pairmate (separation environment). 5 pups were randomly selected from the litter and placed individually at a	Housed together in standard mouse maternity cages.<br>Temp.: 24C<br>12-h light/dark cycle	Parental behavior<br><br>Dam:<br>- Retrieval<br>- Crouching<br>- Grooming<br>- Nest building<br><br>Sire:<br>- Retrieval by separation<br>- After co-housing pairmates<br>- Crouching	Maternal nurturing behavior was observed in dams of all three strains, in a strain-nonspecific fashion, except for the low rate of retrieval by the BALB/c dams. In contrast, paternal behavior was variable between the strains. No retrieval behavior was observed by BALB/c sires.<br>40% of C57BL/6 sires displayed retrieval during reunion after single-separation in new cages. However, isolation together with the partner in new cages did not potentiate but rather decreased this rate to 13.3%. This parental behavior suggests that C57BL/6 males display mate-independent paternal behavior. Interestingly, 38-kHz USVs were not recorded from any dam–sire pairs of C57BL/6 and BALB/c strains separated in new cages for 10 min. These results indicated that pairmate-dependent care is specific to the ICR strain. Therefore, in the following experiments, we examined various critical conditions under which ICR strain males did or did not show paternal behavior.

		tem (model MK-1500; Muromachi Kikai) with functions such as an analog-to-digital converter (192 kHz), frequency filters, a digital fast-Fourier-transform analyzer, and signal input—output terminals.	site remote from the nest in the original cage. The sires were returned to the original home cage or a new cage in the presence of their five biological or foster pups to assess parental behavior. Parental retrieval behavior (percentage of sires exhibiting retrieval) was examined for 10 min following reunion.		- Grooming<br>- Nest building<br><br>Pups<br>- Number of pups per litter<br>- Survival ratio<br>- USVs<br><br>USVs<br>- Number of calls	- Among the mouse strains tested, the mate-dependent paternal retrieval behavior was observed only in the ICR strain, and acquisition of such paternal behavior increased slowly following parturition of the dam;<br>- ICR sires displayed parental retrieval behavior only for their own biological pups<br>- Interaction between the sires and unrelated non-mating dams is not effective and does not involve 38-kHz USVs<br>- It is important for the sire to establish its home cage (territory) by continuous housing to display parental retrieval behavior.

References

Asaba, A., Okabe, S., Nagasawa, M., Kato, M., Koshida, N., Osakada, T., Mogi, K., and Kikusui, T. (2014). Developmental social environment imprints female preference for male song in mice. *PLoS One* 9**,** e87186. doi:10.1371/journal.pone.0087186

Barthelemy, M., Gourbal, B.E., Gabrion, C., and Petit, G. (2004). Influence of the female sexual cycle on BALB/c mouse calling behaviour during mating. *Naturwissenschaften* 91**,** 135-138. doi:10.1007/s00114-004-0501-4

Bell, R.W., Nitschke, W., and Zachman, T.A. (1972). Ultra-sounds in three inbred strains of young mice. *Behav Biol* 7**,** 805-814. doi:10.1016/s0091-6773(72)80172-x

Branchi, I., Santucci, D., Vitale, A., and Alleva, E. (1998). Ultrasonic Vocalizations by Infant Laboratory Mice: A Preliminary Spectographic Characterization under Different Conditions. *Developmental Psychobiology* 33**,** 249-256.

Byatt, S., and Nyby, J. (1986). Hormonal regulation of chemosignals of female mice that elicit ultrasonic vocalizations from males. *Hormones and Behavior* 20**,** 60-72. doi:10.1016/0018-506x(86)90029-2

Chabout, J., Sarkar, A., Dunson, D.B., and Jarvis, E.D. (2015). Male mice song syntax depends on social contexts and influences female preferences. *Front Behav Neurosci* 9**,** 76. doi:10.3389/fnbeh.2015.00076

D'amato, F.R., and Populin, R. (1987). Mother-offspring interaction and pup development in genetically deaf mice. *Behav Genet* 17**,** 465-475. doi:10.1007/BF01073113

D'amato, F., Scalera, E., Sarli, C., and Moles, A. (2005). Pups Call, Mothers Rush: Does Maternal Responsiveness Affect the Amount of Ultrassonic Vocalizations in Mouse Pups? *Behavior Genetics* 35**,** 103-112.

Dizinno, G., and Whitney, G. (1977). Androgen influence on male mouse ultrasounds during courtship. *Hormones and Behavior* 8**,** 188-192. doi:10.1016/0018-506x(77)90035-6

Ehret, G., and Bernecker, C. (1986). Low-frequency sound communication by mouse pups (Mus musculus): wriggling calls release maternal behaviour. *Animal Behaviour* 34**,** 821-830. doi:10.1016/s0003-3472(86)80067-7

Elwood, R.W., and Keeling, F. (1982). Temporal organization of ultrasonic vocalizations in infant mice. *Dev Psychobiol* 15**,** 221-227. doi:10.1002/dev.420150306

Finton, C.J., Keesom, S.M., Hood, K.E., and Hurley, L.M. (2017). What's in a squeak? Female vocal signals predict the sexual behaviour of male house mice during courtship. *Animal Behaviour* 126**,** 163-175. doi:10.1016/j.anbehav.2017.01.021

Hahn, M.E., Hewitt, J.K., Schanz, N., Weinreb, L., and Henry, A. (1997). Genetic and developmental influences on infant mouse ultrasonic calling. I. A diallel analysis of the calls of 3-day olds. *Behav Genet* 27**,** 133-143. doi:10.1023/a:1025637408900

Hahn, M.E., Karkowski, L., Weinreb, L., Henry, A., Schanz, N., and Hahn, E.M. (1998). Genetic and developmental influences on infant mouse ultrasonic calling. II. Developmental patterns in the calls of mice 2-12 days of age. *Behav Genet* 28**,** 315-325. doi:10.1023/a:1021679615792

Hahn, M.E., and Schanz, N. (2002). The effects of cold, rotation, and genotype on the production of ultrasonic calls in infant mice. *Behav Genet* 32**,** 267-273. doi:10.1023/a:1019728813891

Hammerschmidt, K., Radyushkin, K., Ehrenreich, H., and Fischer, J. (2009). Female mice respond to male ultrasonic 'songs' with approach behaviour. *Biol Lett* 5**,** 589-592. doi:10.1098/rsbl.2009.0317

Hanson, J.L., and Hurley, L.M. (2012). Female presence and estrous state influence mouse ultrasonic courtship vocalizations. *PLoS One* 7**,** e40782. doi:10.1371/journal.pone.0040782

Henessy, M.B., Li, J., Lowe, E.L., and Levine, S. (1980). Maternal Behavior, Pup Vocalizations, and Temperature Changes Following Handling in Mice of 2 Inbred Strains. *Developmental Psychobiology* 13**,** 573-584.

Hoffmann, F., Musolf, K., and Penn, D.J. (2009). Freezing urine reduces its efficacy for eliciting ultrasonic vocalizations from male mice. *Physiol Behav* 96**,** 602-605. doi:10.1016/j.physbeh.2008.12.014

Kanno, K., and Kikusui, T. (2018). Effect of Sociosexual Experience and Aging on Number of Courtship Ultrasonic Vocalizations in Male Mice. *Zoolog Sci* 35**,** 208-214. doi:10.2108/zs170175

Kikusui, T., Nakanishi, K., Nakagawa, R., Nagasawa, M., Mogi, K., and Okanoya, K. (2011). Cross fostering experiments suggest that mice songs are innate. *PLoS One* 6**,** e17721. doi:10.1371/journal.pone.0017721

Liang, M., Zhong, J., Liu, H.X., Lopatina, O., Nakada, R., Yamauchi, A.M., and Higashida, H. (2014). Pairmate-dependent pup retrieval as parental behavior in male mice. *Front Neurosci* 8**,** 186. doi:10.3389/fnins.2014.00186

Liu, H.X., Lopatina, O., Higashida, C., Fujimoto, H., Akther, S., Inzhutova, A., Liang, M., Zhong, J., Tsuji, T., Yoshihara, T., Sumi, K., Ishiyama, M., Ma, W.J., Ozaki, M., Yagitani, S., Yokoyama, S., Mukaida, N., Sakurai, T., Hori, O., Yoshioka, K., Hirao, A., Kato, Y., Ishihara, K., Kato, I., Okamoto, H., Cherepanov, S.M., Salmina, A.B., Hirai, H., Asano, M., Brown, D.A., Nagano, I., and Higashida, H. (2013). Displays of paternal mouse pup retrieval following communicative interaction with maternal mates. *Nat Commun* 4**,** 1346. doi:10.1038/ncomms2336

Lupanova, A.S., and Egorova, M.A. (2015). Vocalization of sex partners in the house mouse (Mus Musculus). *Journal of Evolutionary Biochemistry and Physiology* 51**,** 324-331. doi:10.1134/s0022093015040080

Maggio, J.C., and Whitney, G. (1986). Heterosis of adult mouse (Mus musculus) ultrasonic vocalizing. *Behav Genet* 16**,** 493-506. doi:10.1007/BF01074267

Marchlewska-Koj, A., Kapusta, J., and Olejniczak, P. (1999). Ultrasonic Response of CBA Newborn Mice to Bedding Odour. *Behaviour* 136**,** 269-278.

Marconi, M.A., Nicolakis, D., Abbasi, R., Penn, D.J., and Zala, S.M. (2020). Ultrasonic courtship vocalizations of male house mice contain distinct individual signatures. *Animal Behaviour* 169**,** 169-197. doi:10.1016/j.anbehav.2020.09.006

Mogi, K., Takakuda, A., Tsukamoto, C., Ooyama, R., Okabe, S., Koshida, N., Nagasawa, M., and Kikusui, T. (2017). Mutual mother-infant recognition in mice: The role of pup ultrasonic vocalizations. *Behav Brain Res* 325**,** 138-146. doi:10.1016/j.bbr.2016.08.044

Musolf, K., Hoffmann, F., and Penn, D.J. (2010). Ultrasonic courtship vocalizations in wild house mice, Mus musculus musculus. *Animal Behaviour* 79**,** 757-764. doi:10.1016/j.anbehav.20

Nicolakis, D., Marconi, M.A., Zala, S.M., and Penn, D.J. (2020). Ultrasonic vocalizations in house mice depend upon genetic relatedness of mating partners and correlate with subsequent reproductive success. *Front Zool* 17**,** 10. doi:10.1186/s12983-020-00353-1

Noirot, E. (1964). Changes in Responsiveness to Young in the Adult Mouse: The Effect of External Stimuli. *J Comp Physiol Psychol* 57**,** 97-99. doi:10.1037/h0042864

Noirot, E. (1966). Ultra-sounds in young rodents. I. Changes with age in albino mice. *Anim Behav* 14**,** 459-462. doi:10.1016/s0003-3472(66)80045-3

Nyby, J., Wysocki, C.J., Whitney, G., and Dizinno, G. (1977). Pheromonal regulation of male mouse ultrasonic courtship (Mus musculus). *Animal Behaviour* 25**,** 333-341. doi:10.1016/0003-3472(77)90009-4

Okabe, S., Nagasawa, M., Kihara, T., Kato, M., Harada, T., Koshida, N., Mogi, K., and Kikusui, T. (2010). The effects of social experience and gonadal hormones on retrieving behavior of mice and their responses to pup ultrasonic vocalizations. *Zoolog Sci* 27**,** 790-795. doi:10.2108/zsj.27.790

Okabe, S., Nagasawa, M., Kihara, T., Kato, M., Harada, T., Koshida, N., Mogi, K., and Kikusui, T. (2013). Pup odor and ultrasonic vocalizations synergistically stimulate maternal attention in mice. *Behav Neurosci* 127**,** 432-438. doi:10.1037/a0032395

Okon, E.E. (1970a). The effect of envrionmental temperature on the production of ultrasounds by isolated non-handled albino mouse pups. *J Zool* 162**,** 71-83.

Okon, E.E. (1970b). The ultrasonic responses of albino mouse pups to tactile stimuli. *J. Zool., Lond.* 162**,** 485-492.

Pomerantz, S.M., Nunez, A.A., and Bean, N.J. (1983). Female Behavior is Affected by Male Ultrasonic Vocalizations in House Mice. *Physiol and Behavior* 31**,** 91-96.

Robinson, D.J., and D'udine, B. (1982). Ultrasonic calls produced by three laboratory strains of *Mus musculus*. *J Zool* 197**,** 383-389.

Ronald, K.L., Zhang, X., Morrison, M.V., Miller, R., and Hurley, L.M. (2020). Male mice adjust courtship behavior in response to female multimodal signals. *PLoS One* 15**,** e0229302. doi:10.1371/journal.pone.0229302

Supplementary Table 1 – Literature reviewed

Sales, G.D. (1972). Ultrasound and mating behaviour in rodents with some observations on other behavioural situations. *Journal of Zoology* 168**,** 149-164. doi:10.1111/j.1469-7998.1972.tb01345.x

Santucci, D., Masterson, D., and Elwood, R.W. (1994). Effects of age, sex, and odour from conspecific adult males on ultrasonic vocalizations of infant CS1 mice. *Behavioural Processes* 32**,** 285-296.

Smotherman, W.P., Bell, R.W., Starzec, J., Elias, J., and Zachman, T.A. (1974). Maternal responses to infant vocalizations and olfactory cues in rats and mice. *Behav Biol* 12**,** 55-66. doi:10.1016/s0091-6773(74)91026-8

Sugimoto, H., Okabe, S., Kato, M., Koshida, N., Shiroishi, T., Mogi, K., Kikusui, T., and Koide, T. (2011). A role for strain differences in waveforms of ultrasonic vocalizations during male-female interaction. *PLoS One* 6**,** e22093. doi:10.1371/journal.pone.0022093

Thornton, L.M., Hahn, M.E., and Schanz, N. (2005). Genetic and Developmental Influences on Infant Mouse Ultrasonic Calling. III. Patterns of Inheritance in the Calls of Mice 3-9 Days of Age. *Behavior Genetics* 35**,** 73-83.

Uematsu, A., Kikusui, T., Kihara, T., Harada, T., Kato, M., Nakano, K., Murakami, O., Koshida, N., Takeuchi, Y., and Mori, Y. (2007). Maternal approaches to pup ultrasonic vocalizations produced by a nanocrystalline silicon thermo-acoustic emitter. *Brain Res* 1163**,** 91-99. doi:10.1016/j.brainres.2007.05.056

White, N.R., Prasad, M., Barfield, R.J., and Nyby, J.G. (1998). 40- and 70-kHz Vocalizations of Mice (Mus musculus) during Copulation. *Physiology & Behavior* 63**,** 467-473. doi:10.1016/s0031-9384(97)00484-8

Whitney, G., Alpern, M., Dizinno, G., and Horowitz, G. (1974). Female odors evoke ultrasounds from male mice. *Anim Learn Behav* 2**,** 13-18. doi:10.3758/bf03199109

Whitney, G., Coble, J.R., Stockton, M.D., and Tilson, E.F. (1973). Ultrasonic emissions: do they facilitate courtship of mice. *J Comp Physiol Psychol* 84**,** 445-452. doi:10.1037/h0034899

Wohr, M., Dahlhoff, M., Wolf, E., Holsboer, F., Schwarting, R.K., and Wotjak, C.T. (2008). Effects of genetic background, gender, and early environmental factors on isolation-induced ultrasonic calling in mouse pups: an embryo-transfer study. *Behav Genet* 38**,** 579-595. doi:10.1007/s10519-008-9221-4
